# Identifying disease-associated pathways in one-phenotype data based on reversal gene expression orderings

**DOI:** 10.1038/s41598-017-01536-3

**Published:** 2017-05-02

**Authors:** Guini Hong, Hongdong Li, Jiahui Zhang, Qingzhou Guan, Rou Chen, Zheng Guo

**Affiliations:** 1 Department of Bioinformatics, Key Laboratory of Ministry of Education for Gastrointestinal Cancer, Fujian Medical University, Fuzhou, 350108 China; 2 Fujian Key Laboratory of Tumor Microbiology, Fujian Medical University, Fuzhou, 350108 China

## Abstract

Due to the invasiveness nature of tissue biopsy, it is common that investigators cannot collect sufficient normal controls for comparison with diseased samples. We developed a pathway enrichment tool, *DRFunc*, to detect significantly disease-disrupted pathways by incorporating normal controls from other experiments. The method was validated using both microarray and RNA-seq expression data for different cancers. The high concordant differentially ranked (DR) gene pairs were identified between cases and controls from different independent datasets. The DR gene pairs were used in the *DRFunc* algorithm to detect significantly disrupted pathways in one-phenotype expression data by combing controls from other studies. The *DRFunc* algorithm was exemplified by the detection of significant pathways in glioblastoma samples. The algorithm can also be used to detect altered pathways in the datasets with weak expression signals, as shown by the analysis on the expression data of chemotherapy-treated breast cancer samples.

## Introduction

High-throughput biotechnologies such as microarrays and RNA sequencing (RNA_seq) are generating a large volume of genetic data. Such massive data have promoted the development of various pathway enrichment tools^1^, which can be divided into three categories: singular enrichment analysis (SEA), gene set enrichment analysis (GSEA) and modular enrichment analysis (MEA)^2, 3^. SEA usually calculates the enrichment *p*-value for a pathway based on a list of preselected differentially expressed genes (DEGs) using statistical methods such as Student’s *t*-test^4, 5^. GSEA identifies a significant pathway by determining whether the genes of the pathway are ranked at the top or the bottom among all the genes according to their expression differences between two phenotypes^6^. The enrichment calculation in MEA is similar to that in SEA, but the network topology information is integrated^7^. These pathway enrichment tools are effective in identifying disease-associated genes with important pathophysiologic roles.

Tissue biopsy is a conventional method to collect samples for cancer diagnosis, monitoring and pathologic analysis^8^. However, biopsy is frequently very difficult for patients with brain cancer or metastatic cancers^9, 10^, and more challenging for healthy controls. As a consequence, studies for such diseases typically include very few or even no normal controls^11^. This situation poses a serious challenge to the common pathway enrichment tools discussed above, as they all compare quantitative expression levels of pathway genes between two phenotypes^2, 3^. Hereafter we refer to a dataset consisting of samples with only one phenotype (disease) as a one-sided dataset. The control samples for the same disease available in other datasets cannot be incorporated into a one-sided dataset because the quantitative expression values are sensitive to the so-called batch effects between different experiments^12, 13^. Datasets from the Cancer Genome Atlas database (TCGA) database^14^ should also be considered as one-sided, since TCGA samples were derived from different institutions and processed in different batches. Therefore, the DEGs detected directly between tumor samples and normal controls from TCGA are questionable without appropriate batch adjustment^15^. However, batch adjustments may be biased if study groups are not evenly distributed across batches^15^.

To tackle the above problem, some studies have used the within-sample relative expression orderings (REOs) instead of the quantitative expression values for disease screening^16, 17^ and gene signaling network analysis^18^. We previously developed a tool, *individPath*, to identify patient-specific dysregulated pathways based on reversal REOs in an individual sample compared with the highly stable REOs identified from a large cohort of normal samples which were accumulated previously from various sources^19^. Compared with the algorithms based on the quantitative expression values, the REO-based algorithms have some unique advantages, including insensitive to batch effects, free of between-sample data normalization, reproducible across independent data^17, 20^ and reuse of accumulated data^21, 22^. Therefore, for a one-sided disease dataset, it is intuitive to compare the differences between the REOs in diseased samples and the REOs in control samples which may come from an independent dataset, to identify whether a pathway is altered by the disease or not.

We developed a tool, *DRFunc*, to identify the pathways which are significantly enriched with differential REOs of the pathway member genes. Using two independent microarray datasets for gastric cancer, lung cancer and breast cancer, respectively, we demonstrated that differential REOs between diseased samples and control samples were reproducible for independent datasets. These differential REOs were preserved even after the control or case samples were changed with the corresponding control or case samples from the other dataset for the same cancer. Using two RNA-seq datasets from TCGA, we showed that differential REOs identified from the sequence-based data are also highly reproducible in the array-based data. The usage of this tool was further exemplified by applying to a one-sided glioblastoma dataset to detect significantly altered pathways. For two expression datasets collected for patients with breast cancer receiving chemotherapy, *DRFunc* could detect significant pathways which were elusive for the traditional tools which depend on the pre-selected DEGs, in particular when few DEGs could be identified.

## Materials and Methods

### Data source and data preprocessing

We collected 11 microarray datasets from the Gene Expression Omnibus (GEO) database (http://www.ncbi.nlm.nih.gov/geo/), as shown in Table 1. All of the datasets were measured by the Affymetrix platforms. The raw data were preprocessed by the Robust Multi-array Analysis algorithm^23^. The SOURCE database^24^ was used for mapping CloneIDs to GeneIDs. From the Cancer Genome Atlas database (TCGA), two RNA-seq datasets were downloaded (see Table 1). The RNA-seq datasets were measured by the Illumina HiSeq platform. The raw data were normalized^25^ using the edgeR BioConductor package^26^.

**Table 1:** Datasets used in this study.

Dataset^a^	Case	Control	Data source	Platform
GC_38-31_	38	31	GSE13911	GPL570
GC_12-15_	12	15	GSE19826	GPL570
LC_91-65_	91	65	GSE19188	GPL570
LC_60-60_	60	60	GSE19804	GPL570
BC_12-27_^ER^	12	27	GSE10810	GPL570
BC_34-17_^ER^	34	17	GSE42568	GPL570
GBM_34-13_	34	13	GSE50161	GPL570
GBM_70-0_	70	0	GSE53733	GPL570
BC_68-46_^Response^	68	46	GSE20194	GPL96
BC_61-19_^Response^	61	19	GSE20271	GPL96
LUAD_125-37_	125	37	TCGA	HiSeq2000
CRC_32-32_	32	32	GSE8671	GPL570
COAD_285-41_	285	41	TCGA	HiSeq2000

Denotes: ^a^GC denotes gastric cancer, LC denotes lung cancer, BC denotes breast cancer, ER denotes estrogen receptor, GBM denotes glioblastoma, LUAD denotes lung adenocarcinoma, CRC denotes colorectal cancer, and COAD denotes colon adenocarcinoma. We referred to each dataset using the following nomenclature: cancer type followed by the number of case and control samples separated by a hyphen sign.

### Pathway databases

The gene ontology (GO), Kyoto encyclopedia of genes and genomes (KEGG) and the Molecular Signatures Database (MSigDB) were used for enrichment analysis in *DRFunc*. Taking the C2 gene sets of MSigDB as an example, 1330 canonical pathways (as of 16 February 2016) were download from the GSEA website. For a given dataset, all of the measured genes which were annotated in the 1330 pathways were considered as the background genes. In total, there were 8039, 6825 and 8548 genes for the GPL570, GPL96 and Illumina HiSeq2000 platforms, respectively.

### Identification of differential REOs between two phenotypes

Given that the expression values of a gene pair (*i*, *j*) are denoted as (*G*_*i*_, *G*_*j*_), *R*_*ij*_, which is 1 if *G*_*i*_ > *G*_*j*_ and 0 if *G*_*i*_ < *G*_*j*_ within one sample, is defined as the REO of the gene pair. If two genes have the same expression value, the pair is excluded from analysis. For a dataset with *n* cases and *m* controls, differential REOs are identified through the following steps. (1) Calculate the values of *R*_*ij*_ (0 or 1) for all pairs in each sample. (2) Count the frequencies of the binary values (1 or 0) of *R*_*ij*_ for each pair (*i*, *j*) in each phenotype. For example, there are *n*_1_ samples with *R*_*ij*_ = 1 and *n*_2_ samples with *R*_*ij*_ = 0 in the case group (*n*_1_ + *n*_2_ = *n*), and *m*_1_ samples with *R*_*ij*_ = 1 and *m*_2_ samples with *R*_*ij*_ = 0 in the control group (*m*_1_ + *m*_2_ = *m*). (3) Test the null hypothesis that the frequencies have no association with phenotype (case or control) using the Fisher’s exact test. (4) Select differentially ranked (DR) gene pairs. After the Fisher’s exact test is done for all the pairs, the *p*-values are corrected to control the false discovery rate (FDR)^27^. A gene pair is considered as a DR gene pair if the adjusted *p*-value is less than 5%. Furthermore, for a DR gene pair, there are two possible patterns. If *n*_1_/*n*_2_ > *m*_1_/*m*_2_, the pair is called as Pattern 1, otherwise it is called as Pattern 2.

### Reproducibility of DR gene pairs

The binomial test is employed to evaluate the reproducibility between the two lists of DR gene pairs. If a gene pair has the same pattern of reversal REO in the two lists, this gene pair is considered as a concordant gene pair. If two lists of DR gene pairs have *M* common pairs, the probability of observing at least *M*_1_ concordant gene pairs by chance is calculated by the following cumulative binomial distribution model,

1 $$P=\sum _{i={M}_{1}}^{M}(\begin{array}{c}M\\ i\end{array}){p}_{0}^{i}{(1-{p}_{0})}^{M-i}$$

where *p*_0_ is the probability for a random gene pair to be a concordant gene pair by chance between two lists (here *p*_0* = *_0.5 since there are only two mutual-exclusive outcomes, Pattern 1 or Pattern 2, of a DR gene pair). The concordant ratio of these two lists of DR gene pairs is defined as *M*_1_/*M*. The two lists of DR gene pairs are considered significantly reproducible if *P* < 0.05.

### Pathway enrichment analysis based on DR gene pairs

If *k* gene pairs are DR gene pairs from *n* background gene pairs, the probability of observing at least *x* DR gene pairs in a pathway with a total of *m* background gene pairs by chance is given by the cumulative hypergeometric distribution function as follows,

2 $${\rm{P}}=1-\sum _{{\rm{i}}=0}^{{\rm{x}}-1}\frac{(\begin{array}{c}m\\ i\end{array})(\begin{array}{c}n-m\\ k-i\end{array})}{(\begin{array}{c}n\\ k\end{array})}$$

The number of the background gene pairs (*n*) is equal to *N*(*N* − 1)/2, where *N* represents the number of the background genes. The pathways significantly enriched with DR gene pairs were identified after multiple testing adjustments with FDR < 5%^27^.

**Figure 1:**
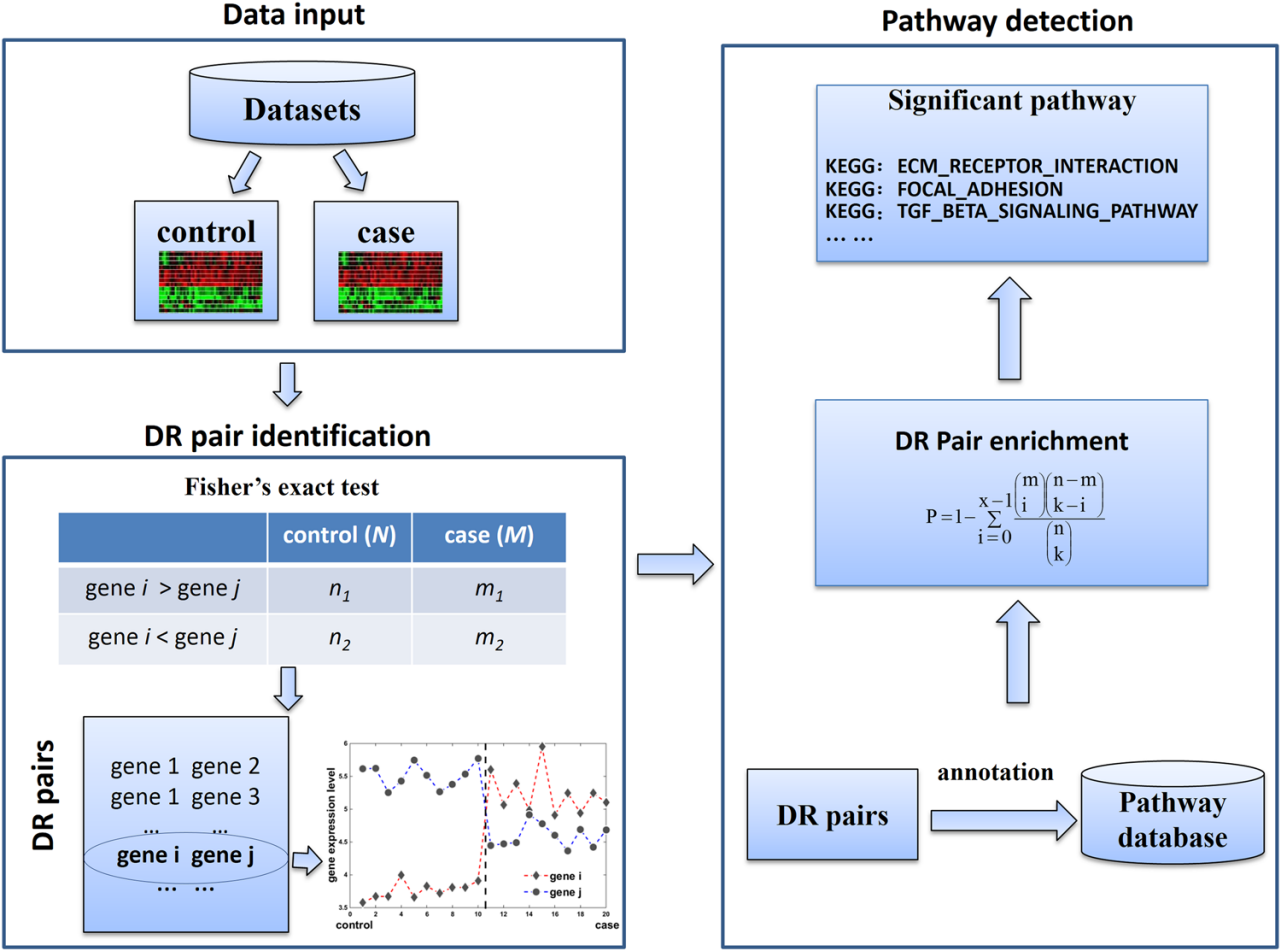
Flowchart of *DRFunc*. The *DRFunc* algorithm includes three steps: input of expression profiles for case and control samples (from the same or different experiments), DR gene pair identification, annotation and detection of significant pathways.

Figure 1 shows the flowchart of *DRFunc*. The identification of DR gene pairs and detection of significant pathways were implemented in an open-source R package which is available at https://github.com/keyougu/DRFunc.git.

## Results

### Reproducible DR gene pairs identified between tumor and normal samples

The datasets of gastric cancer, lung cancer and ER^−^ breast cancer which have large sample size were first used to test whether DR gene pairs could be reproducibly identified in different subsets of the same parent dataset. For each dataset, the tumor samples and control samples were randomly divided into two subsets with approximately equal sample size respectively. For example, the 38 tumor samples in GC_38-31_ were divided into two groups with 19 samples each, while the 31 normal samples were divided into two groups with 15 samples and 16 samples respectively. They formed two subsets, one with 19 tumor samples and 15 normal samples and the other with 19 tumor samples and 16 normal samples. From these two subsets, DR gene pairs were identified and compared. This procedure was repeated 100 times. The result showed that the identified DR gene pairs were highly reproducible, with an average concordant ratio of 99.99% for the dataset of GC_38-31_ (see Table 2). Similar results were observed for LC_91-65_ and BC_34-17_^ER^ (see Table 2). These results show that the identified DR gene pairs are highly reproducibly within one dataset.

**Table 2:** Mean and standard deviation of the number of DR gene pairs identified from random subsets.

Dataset	#DR pair	#Overlapped pair	#Concordant pair	Concordant ratio
GC_38-31_	1054900 ± 237429			
	1169868 ± 271089	586201 ± 36373	586198 ± 36373	0.9999 ± 8.42 × 10^−6^
LC_91-65_	5211347 ± 236859			
	4983364 ± 256758	4078924 ± 69845	4078880 ± 69861	0.9999 ± 6.74 × 10^−6^
BC_34-17_^ER^	1199844 ± 328353			
	1046124 ± 308752	595768 ± 86284	595768 ± 86284	0.9999 ± 2.2 × 10^−16^

Next, the reproducibility was analyzed for the DR gene pairs identified from different experimental datasets for the same cancer. As shown in Table 3, in the dataset GC_12-15_, 249,379 DR gene pairs were identified between gastric tumor samples and normal controls, among which 75.67% were also detected as DR gene pairs in dataset GC_38-31_. Among the overlapped DR gene pairs, 99.97% showed the concordant REOs in the two gastric datasets, which could not happen by random chance (*p* < 2.2 × 10^−16^, binomial test). Similar result was observed in the two datasets for lung cancer. In the dataset LC_60-60_, the one with smaller sample size of the two datasets, 75.18% of the detected DR gene pairs were also identified in the dataset LC_91-45_ which has larger sample size than LC_60-60_, and 98.39% of the overlapped DR gene pairs had the concordant REOs in the two datasets, which could not happen by random chance (*p < *2.2 × 10^−16^, binomial test). In the two datasets for ER^−^ breast cancer, the concordant ratio was 99.84%. These results indicate that extensive disruptions of gene REOs existed in tumor samples and such disrupted REOs were reproducible in different datasets. The number of genes in each DR gene pair list were provided in Supplementary file, Table S1.

**Table 3:** Concordance of DR gene pairs identified for each cancer dataset.

Dataset	#DR pair	#Overlapped pair	#Concordant pair	Concordant ratio
GC_12-15_	249379	188706	186655	0.9997
GC_38-31_	3060133			
LC_60-60_	5035285	3785548	3724663	0.9839
LC_91-65_	7977878			
BC_12-27_^ER^	2527003	1406505	1404282	0.9984
BC_34-17_^ER^	3087813			

A further test on reproducibility was carried out to exchange the case and/or control samples between two datasets for the same cancer type. The DR gene pairs identified from the newly exchanged datasets were compared with the DR gene pairs identified from the original datasets. As shown in Table 4, 3,870,438 DR gene pairs were identified in the merged dataset GC_12-31_ by integrating the normal samples from GC_38-31_ and the tumor samples from GC_12-15_, among which 163,670 were included in the DR gene pairs identified from the original dataset GC_12-15_. Similarly, 4,523,783 DR gene pairs were identified in the merged dataset GC_38-15_, among which 1,560,772 were found in the original dataset GC_38-31_. With only the control samples exchanged, the concordant ratios of DR gene pairs between the new datasets and their respective original datasets were 99.19% and 92.41% (Table 4), which were comparable to the concordant ratio between the two original datasets (99.97%) and could not happen by random chance (*p* < 2.2 × 10^−16^, binomial test). For lung cancer, the concordant ratios between the control-exchanged datasets and the original datasets were 95.41% and 95.19% respectively, which were also comparable to the concordant ratio between the two original datasets (98.39%). For the two control-exchanged datasets for ER^−^ breast cancer, the concordant ratios were 98.88% and 97.47% respectively, also comparable to the concordant ratio between the two original datasets (99.84%). The detected DR gene pairs were also highly reproducible in the case-exchanged datasets: the minimum concordant ratio was as high as 97.06% (see Supplementary file, Table S2). These analyses further indicate that differential REOs for a specific tumor type could be reproducibly detected from independent datasets of different sources. Therefore, when focusing on the REOs of genes, tumor samples and normal samples measured by different studies can be directly compared.

**Table 4:** Concordance of DR gene pairs identified from datasets with the same case samples but different control samples.

Dataset	#DR pair	#Overlapped pair	#Concordant pair	Concordant ratio
GC_12-31_	3870438	163670	162305	0.9919
GC_12-15_	249379			
GC_38-15_	4523783	1560772	1442242	0.9241
GC_38-31_	3060133			
LC_60-65_	7387229	3982182	3799350	0.9541
LC_60-60_	5035285			
LC_91-60_	8935664	6335001	6030374	0.9519
LC_91-65_	7977878			
BC_12-17_^ER^	2649823	1130216	1117603	0.9888
BC_12-27_^ER^	2527003			
BC_34-27_^ER^	6630077	2393323	2332764	0.9747
BC_34-17_^ER^	3087813			

### Performance of *DRFunc* in detecting significant pathways

**Figure 2:**
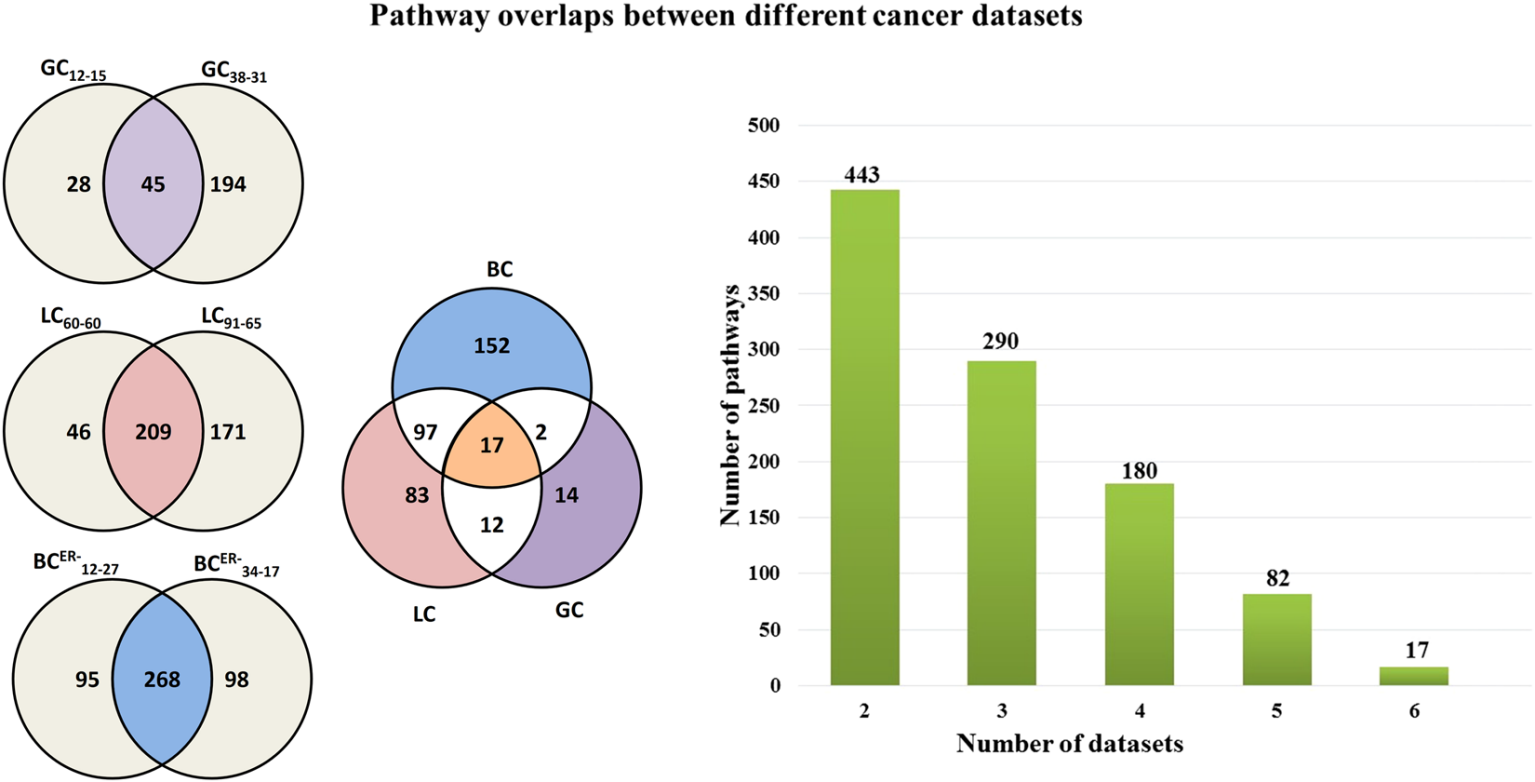
Overlaps of significant pathways detected for the three cancer types. The bar plot shows the number of significant pathways (*y*-axis) shared by at least two, three, four, five and six datasets (*x*-axis) for BC, LC and GC.

Significant pathways were detected from the 1330 MSigDB C2 collection by employing the cumulative hypergeometric distribution test implemented in *DRFunc*. With FDR < 5%, 73 and 239 pathways were detected, respectively, to be significantly enriched with the DR gene pairs identified from GC_12-15_ and GC_38-31_. For lung cancer, 255 and 380 pathways were detected in LC_60-60_ and LC_91-65_, respectively. For ER^−^ breast cancer, 363 and 366 pathways were detected for BC_12-27_^ER^ and BC_34-17_^ER^, respectively. The overlapped pathways were shown in Fig. 2. The pathway names were listed in Supplementary file. Notably, there were 17 pathways commonly detected in the six datasets for the three caner types, including the ECM receptor interaction, focal adhesion pathways in KEGG, the RB1 and integrin related pathways in PID. There were 290 pathways commonly detected for at least two cancer types, indicating that different cancers may have many similar enriched pathways. These results suggest that the REOs of genes in many pathways were significantly disrupted under cancerous conditions, and *DRFunc* could capture such disruptions.

**Figure 3:**
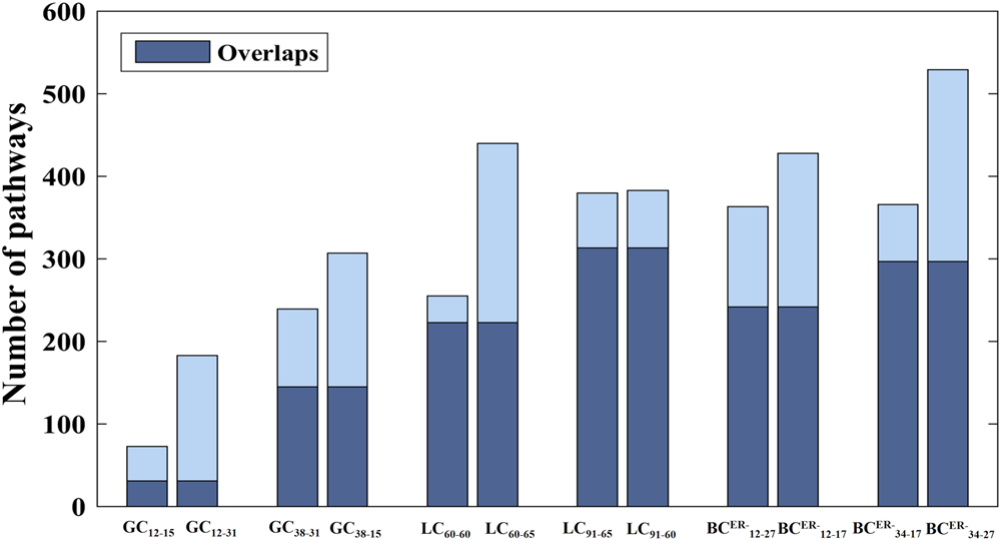
Numbers of significant MSigDB pathways detected for GC, LC, and BC.

With FDR < 5%, 183 and 307 significant pathways were detected, respectively, in the control-exchanged datasets GC_12-31_ and GC_38-15_ for gastric cancer. In the two control-exchanged datasets for lung cancer, 440 and 383 pathways were detected, respectively, and in the two control-exchanged datasets for ER^−^ breast cancer, 428 and 529 pathways were detected, respectively. Figure 3 show the number of overlapped significant pathways detected for the original datasets and for the control-exchanged datasets. This indicate that integration of cancer samples and control samples from different datasets is feasible using the DR gene pairs in order to detect significant pathways.

One RNA-seq dataset with 125 adenocarcinoma samples and 37 normal controls from 20 batches, denoted as LUAD_125-37_ were downloaded from TCGA (see Table 1). By applying *DRFunc*, 7,661,296 DR gene pairs were identified and they were significantly enriched in 530 pathways. These results were compared with those obtained from the microarray datasets. There were 2,656,494 overlapped gene pairs between the 5,035,285 DR gene pairs identified in LC_60-60_ and the 7,661,296 DR gene pairs identified in LUAD_125-37_. Among these overlapped DR gene pairs, 97.48% showed the concordant REOs between the microarray-based and sequence-based results, which could not happen by random chance (*p* < 2.2 × 10^−16^, binomial test). Among the 255 pathways detected for LC_60-60_, 215 pathways were also detected for LUAD_125-37_. There were 3,611,571 pairs overlapped between the DR gene pairs identified in LC_91-65_ and in LUAD_125-37_ with a concordant ratio of 95.58% (*p* < 2.2 × 10^−16^, binomial test). Of the 380 pathways detected in LC_91-65_ 291 were also detected in the sequence-based dataset LUAD_125-37_. Similar results were observed for colorectal cancer, for which one microarray dataset and one RNA-seq dataset were collected (see Table 1). The concordant ratio of the DR gene pairs between CRC_32-32_ and COAD_285-41_ was as high as 97.81%. Of the 283 significant pathways detected in CRC_32-32_, 205 were also detected in COAD_285-41_. These results suggest that differential REOs between case and control samples identified by *DRFunc* had cross-platform reproducibility.

To address whether pathways detected by using *DRFunc* is robust, random experiments were performed by adding different proportions of arbitrarily chosen gene pairs from the background into the real DR gene pairs identified between cases and controls in each dataset. The result showed that the pathways detected by *DRFunc* were robust (see Supplementary file). This conclusion is consistent with the viewpoint that functional categories are robust to different levels of noises^28^.

### Application of *DRFunc* to one-sided GBM data

**Figure 4:**
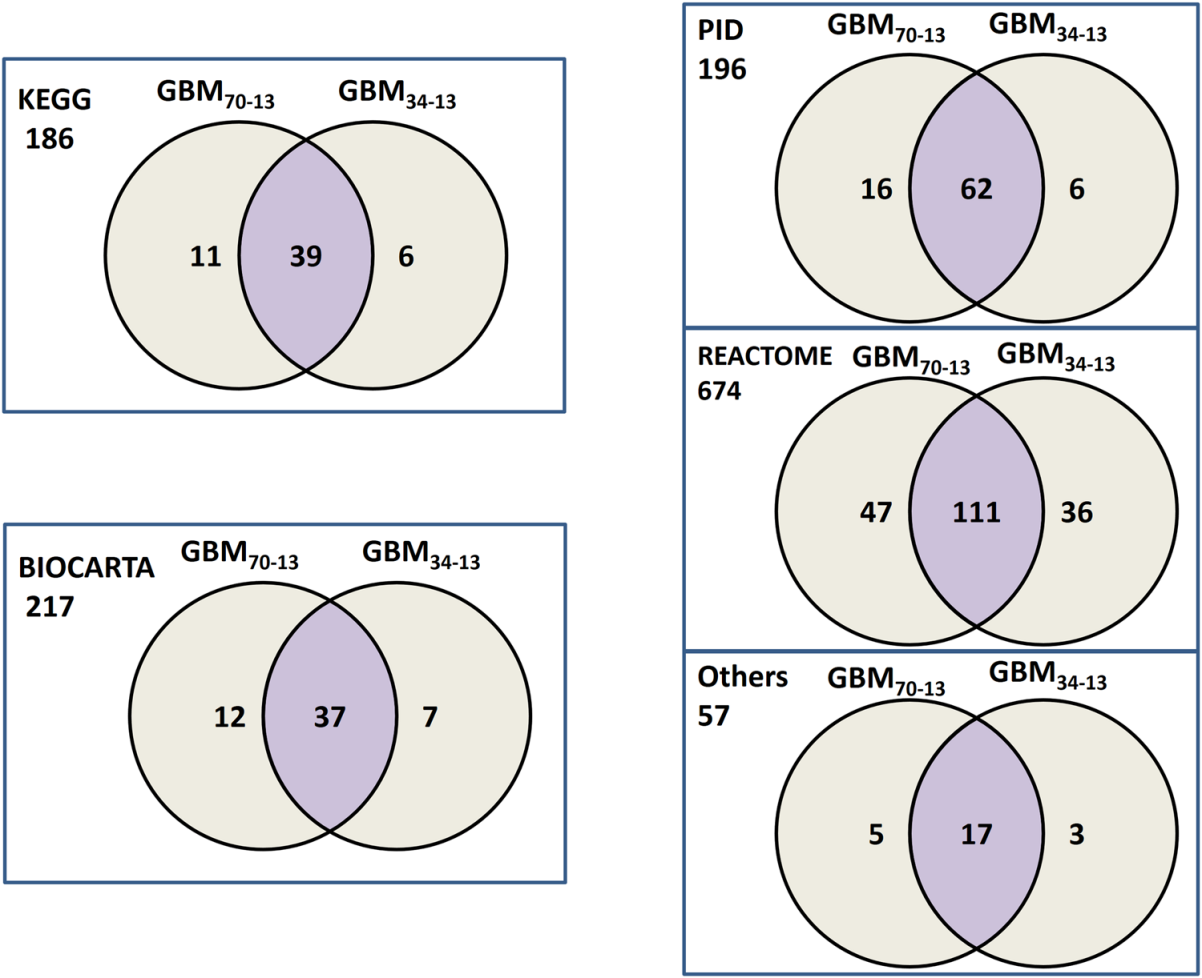
Venn diagrams for the number of significant MSigDB pathways detected for GBM. The 1330 significant MSigDB pathways were divided into five groups according to the source databases, including Biocarta, KEGG, PID, Reactom and the others.

To demonstrate the applicability of *DRFunc* in one-sided data, two datasets, GBM_70-0_ with 70 samples of primary GBM samples^10^ and GBM_34-13_ with 34 primary GBM samples and 13 normal brain tissue samples^29^, were collected. By integrating the GBM samples in GBM_70-0_ and the normal samples in GBM_34-13_ (denoted as integrated GBM_70-13_ dataset) 5,756,553 DR gene pairs were identified. In the dataset GBM_34-13_ itself, 3,659,102 DR gene pairs were identified, among which 80.84% overlapped with the former DR gene pairs. In particular, 99.85% of the overlapped gene pairs had the concordant REOs in the two groups of GBM patients. With FDR < 5%, 363 pathways were detected to be significantly enriched with the DR gene pairs identified in the integrated GBM_70-13_ dataset. Meanwhile, 324 pathways were identified in GBM34-13, among which 266 were also detected in the integrated GBM_70-13_ dataset. They were listed in the supplementary material. As the 1330 MSigDB C2 pathways integrated several online pathway databases with redundancy, the number of the detected pathways was also showed in Fig. 4, grouped by the pathway database source. Many of the pathways were found to be associated with GBM in literature, including the BioCarta EGF pathway and MTOR pathway^30^, the KEGG P53 signaling pathway and the PID TGF-β and Ras signaling pathway^31^.

Since the number of GBM samples in GBM_70-0_ was approximately twice of that in GBM_34-13_, we performed resampling experiments to evaluate the effect of sample size. A subset of 34 GBM samples were randomly extracted from GBM_70-0_ and integrated with the normal samples from GBM_34-13_ for DR gene pair identification and significant pathway detection. This random experiment was repeated 100 times. The average number of DR gene pairs identified in the resampling experiments was 5,046,700, and the average concordant ratio was 99.91% with the 3,659,102 DR gene pairs identified in GBM_34-13_. The average number of significant pathways detected in the 100 resampling experiments was 374.80, and the average of overlapped pathways were 271.40 with the 324 pathways detected in GBM_34-13_. These results suggest that *DRFunc* could be used in one-sided data to detect the underlying dysregulated and disease associated biological pathways.

The *DRFunc* algorithm was compared with two pathway analysis algorithms, SEA^5^ and GSEA^6^. As no controls were collected in GBM_70-0_, the traditional SEA analysis could not be applied^5^. In GBM_34-13_, 8,731 DEGs were identified (FDR < 5%, Student’s *t*-test). Using SEA, these DEGs were significantly enriched in 41 MSigDB pathways^5^, much fewer than the number of pathways detected by *DRFunc* in GBM_34-13_. Notably, the above mentioned EGR, MTOR, P53, TGF-β and Ras signaling pathways detected by *DRFunc* were not included in these 41 pathways. In contrast, 32 of these 41 pathways were also detected by *DRFunc*. When using GSEA, even with FDR < 25%, no significant pathways were detected in GBM_34-13_. These results suggest that the rank-based tool *DRFunc* could identify much more biologically meaningful pathways than the traditional enrichment analysis.

*DRFunc* can detect pathways with only a few DEGs, since a dysregulated gene with a large change in quantitative expression level may result in many DR gene pairs. For example, the BioCarta EIF4 pathway, which mainly describes the regulation of eIF4E and p70 S6 kinase, contained 24 genes measured in the GBM_34-13_ dataset, among which only nine genes were identified as DEGs using Student’s *t*-test. The percentage of DEGs was only 37.50% in this pathway, while the percentage of DEGs was 47.54% in the background. Thus this pathway was not detected as significant by SEA. In contrast, these 24 genes formed 276 gene pairs, among which 53 were identified as DR gene pairs in GBM_34-13_. Therefore the pathway was detected to be significant by *DRFunc*. It has been reported that the overexpression of eIF4E could cause oncogenic transformation and elevated eIF4E protein levels were found in many human cancers including GBM^32, 33^. Interestingly, PRKCB in this pathway involved in 20 DR gene pairs, and its average expression level was higher than the expression levels of all its 20 partner genes in the normal samples but became lower than the expression levels of all its 20 partner genes in the GBM samples. That is to say that PRKCB was down-regulated greatly in GBM. This was consistent with the expression level changes as observed in GBM_34-13_ and literature results reported for GBM^34^ as well as for other cancer types^35^. Similarly, WIF1 in KEGG WNT signaling pathway was found to be down-regulated greatly in GBM by comparing its expression level with those of 127 partner genes. This was consistent with the result reported previously^36^. These two examples suggest that such strongly dysregulated genes could lead to a high appearance frequency in DR gene pairs and make the associated pathways detectable by *DRFunc*.

### Application of *DRFunc* to preoperative chemotherapy response data of breast cancer

A pathway with only a few DEGs cannot be detected by SEA but it may be significantly enriched with DR gene pairs. This hinted us that *DRFunc* might be able to capture functional disruptions in data with weak expression signals. Breast cancer patients with the pathological complete response (pCR) have a favorable prognosis compared to patients with residual disease (RD) and our previous analysis has shown that expression differences between these two conditions could be weak^37^. Two gene expression datasets were collected for preoperative chemotherapy response of breast cancer (see Table 1) to test whether *DRFunc* could identify such weak expression signals. Using Student’s *t*-test with FDR < 5%, one gene was identified as DEG between 61 RD patients and 19 pCR patients in the dataset BC_61-19_^Response^, indicating that expression differences between these two conditions were small. Because there was only one DEG, it was unable to detect significantly dysregulated pathways using SEA. However, using *DRFunc*, 9,569 DR gene pairs were identified with FDR < 5%, which significantly enriched in 38 MSigDB pathways (Supplementary file). In BC_68-46_^Response^, 321 genes were identified as DEGs between 68 RD patients and 46 pCR patients, significantly enriched in 28 MSigDB pathways as detected by SEA. With FDR < 5%, *DRFunc* identified 90,561 DR gene pairs, which significantly enriched in 84 MSigDB pathways (Supplementary file). When using GSEA, with FDR < 5%, no significant pathways were detected in either of the two datasets. These results suggested that the rank-based algorithm *DRFunc* could identify more biological pathways than the traditional enrichment analysis, especially when the expression differences were not significant.

## Discussion

Gene expression profiling for only one phenotype is frequently seen in experimental design when sampling of normal control tissues is difficult due to the invasive nature of biopsy^9^. For such one-sided data, current functional enrichment analysis tools which focus on quantitative expression differences between two phenotypes have difficulty in finding phenotype-related functional pathways. The within-sample REOs have been found robust against systematic batch effects and transferable among independent datasets which enables the reuse of accumulated samples^19, 21, 38^. In the present work, we proposed an REO-based algorithm *DRFunc*, which could robustly identify the underlying disturbed pathways from such one-sided dataset by integrating control samples of the same tissue measured by other independent experiments.

Our analyses showed that the DR gene pairs identified by *DRFunc* for gastric cancer, lung cancer and ER^−^ breast cancer were highly reproducible among independent datasets and among datasets with case-control samples integrated from different studies. The comparison between microarray-based and sequence-based data for lung cancer and colorectal cancer also suggested the high cross-platform reproducibility of DR gene pairs identified by *DRFunc*. Such consistent DR gene pairs were previously observed among datasets generated by different microarray platforms^39^.

The power of *DRFunc* may be influenced by the sample size in detecting DR gene pairs. For example, with FDR < 5%, 249,379 DR gene pairs were identified from the smaller-size dataset of GC_12-15_, which was less than ten-fold of the number of DR gene pairs (3,060,113) identified from the larger-size dataset of GC_38-31_ (Table 3). The insufficient sample size for any of the datasets will reduce the number of overlapped DR gene pairs^40^. Although the numbers of DR gene pairs in GC_12-31_ and GC_38-15_ were almost the same, the overlapped DR gene pairs between GC_12-31_ and GC_12-15_ was ten-fold less than the number of overlapped DR gene pairs between GC_38-15_ and GC_38-31_ (Table 3). The reduced power of DR gene pair identification will ultimately reduce the power of significant pathway detection. As shown in Fig. 3, only 73 pathways were significantly enriched with the DR gene pairs identified in GC_12-15_, whereas 239 significant pathways were detected in GC_38-31_.

Some DR gene pairs may not overlap between different experiments. This is probably due to the fact that an experiment cannot capture all disease-associated differential signals, thus different experiments for the same disease may capture only partial DEGs each^40^. For example, among the top 100 genes with the highest appearance frequencies in the DR gene pairs identified only in GC_38-31_, not in GC_12-15_, 65 were identified as DEGs in GC_38-31_, not in GC_12-15_ (Student’s *t*-test, FDR < 5%). Non-overlapped DEGs would result in non-overlapped DR gene pairs between different experiments.

Due to the above mentioned reasons, ultimately, some significant pathways cannot overlap between different datasets for the same disease. The problem of pathway overlaps has been discussed, and it has been suggested that the significant pathways could be rather functionally similar by reducing their corresponding statistical significance levels^5, 41^.

It has been reported that many confounding factors such as gender and ethnicity may lead to gene expression differences among individuals^42–45^. Therefore, the two datasets (LC_91-65_ and LC_60-60_) for lung cancer with larger sample sizes were used to evaluate whether heterogeneous gene expression exists among normal samples. Information on the normal samples of the two lung cancer datasets was available in Supplementary file, Table S3. The normal samples in LC_91-65_ were obtained from 41 males, 11 females and 13 samples without gender information. Comparing the gene expression profiles of the 41 males and 11 females, only 0.03% of the background gene pairs could be identified as DR gene pairs. However, when comparing the 11 normal female samples of Caucasian in LC_91-65_ to the 60 normal female samples of Chinese in LC_60-60_, about 3.50% of the background gene pairs were found as DR gene pairs. This result indicates that ethnicity might be a confounding factor, which might introduce some disease-irrelevant DR gene pairs. Consequently, when applying *DRFunc* to detect DR gene pairs for significant pathway detection, some disease-irrelevant pathways may creep in.

In spite of this, comparing to the traditional pathway enrichment analysis methods based on quantitative gene expression levels, which have limited usage with one-sided data, *DRFunc* has superiority in providing candidate pathways. To evaluate whether a significant pathway detected by *DRFunc* have specific biological implications or not, it is required to generate some biological hypotheses for wet lab experiment (such as Q-PCR) validation^5^. In this paper, we firstly showed that, in the one-sided GBM data, 266 of the 363 pathways detected in GBM_70-13_ could be reproducibly detected in the other dataset GBM_34-13_, which shares the same normal samples. Then, in the two application examples, besides the pathways already discussed in the Result section, we have additionally found evidence from published literature for the top 10 most significant pathways to support their association with the corresponding phenotype (Supplementary file). Further, to show that the significant pathways detected by *DRFunc* could not be detected if no phenotype differences exist, we have additionally performed random experiments by randomly reassigning labels to the disease and normal samples. By independently permuting the 70 GBM samples from GBM_70-0_ and 13 normal samples from GBM_34-13_ for 100 times, only 10.70 significant pathways were detected on average. When applying the same randomization procedure to BC_68-46_^Response^ dataset, only 7.02 pathways were detected on average. All these results support the ability of *DRFunc* in providing candidate disease-associated significant pathways using gene expression data even the one-sided data. Finally, if only a limited (or insufficient) number of normal control samples for a tissue were obtained in a study, normal samples from other independent datasets should be integrated for DR gene pair identification to reduce disease-irrelevant DR gene pairs introduced by population variations.

In conclusion, through detection of DR gene pairs between diseased samples and normal controls collected from different experiments, disease-relevant pathways can be identified, which provide functional insights into the disease mechanism. The usage of the DR gene pairs instead of the DEGs enables us to make adequate use of the large one-sided disease samples and the samples with weak expression signals available in public data archives. This may facilitate many downstream analyses such as survival prediction. Our algorithm also provides a new tool for comparing transcriptional expression profiling of genes between two groups of samples from the same or different experiments.

## Electronic supplementary material

Supplementary file
