# Supplementary material for: Identifying disease-associated pathways in one-phenotype data based on reversal gene expression orderings
Source: Sci Rep. 2017 May 2;7:1348. doi: 10.1038/s41598-017-01536-3 (PMC5431047; doi:10.1038/s41598-017-01536-3)
Supplement: Supplementary file 1 — Supplementary file [file 41598_2017_1536_MOESM1_ESM.pdf]

Supplementary Contents

Identifying disease-associated pathways in one-phenotype data based on reversal gene expression orderings

Guini Hong[*,+], Hongdong Li[+], Jiahui Zhang, Qingzhou Guan, Rou Chen, Zheng Guo[*]

Corresponding author: gnhong@fjmu.edu.cn and guoz@ems.hrbmu.edu.cn

Tolerance of *DRFunc* to noise

Supplementary Methods

For each dataset, to test whether the significant pathways detected by DR gene pairs could be able to tolerate noises, we did random experiments by adding particular proportions of background gene pairs into the real DR gene pairs identified between case and control samples for the enrichment analysis of pathways. In each random experiment, for a dataset, the real DR gene pairs were first excluded from the background gene pairs, and a given proportion of random gene pairs were then arbitrarily selected from the remaining background gene pairs and added into the list of real DR gene pairs. Pathway enrichment analysis using *DRFunc* was performed for the DR gene pairs with random gene pairs. The overlaps between the significant pathways detected by the DR gene pairs with random gene pairs and the real significant pathways detected by the DR gene pairs without random gene pairs were calculated. This procedure was repeated 100 times. The average pathway overlaps and the average numbers of pathways detected by the DR gene pairs with random gene pairs were calculated. Their ratio was defined as the reproducibility ratio.

Supplementary Results

To address whether pathways detected by using *DRFunc* is robust, we performed random experiments by adding different proportions of arbitrarily chosen gene pairs from the background into the real DR gene pairs identified between cases and controls in each dataset for gastric, lung and ER- breast cancer for pathway enrichment analysis (see Suplementary Methods). The proportions of random gene pairs to be added to the real DR gene pairs were set as 5%, 10%, 15% and 20%. As shown in Table S4, for each cancer type, at least 97.40% of the significant pathways enriched by DR gene pairs with 5% of random gene pairs were detected by the real DR gene pairs without random gene pairs, reproducing at least 93.62% of the real significant

pathways. Even with 20% of random gene pairs, at least 96.60% of the significant pathways detected in random experiments overlapped with the real significant pathways detected by the DR gene pairs without random pairs. And these significant pathways reproduced at least 88.09% of the real significant pathways. These results suggested that, even REOs of genes in samples from different datasets were affected by a certain amount of random noise, *DRFunc* algorithm could still detect most of significant pathways supposed to be detected.

Supplementary Table S1. The number of genes involved in each DR gene pair list

Original dataset	#DR pair	#Gene	#Background genes
GC_{12-15}	249379	7860	8039
GC_{38-31}	3060133	8011	8039
LC_{60-60}	5035285	8036	8039
LC_{91-65}	7977878	8039	8039
BC_{12-27}^{ER}	2527003	8014	8039
BC_{34-17}^{ER}	3087813	8039	8039
Merged dataset			
GC_{12-31}	3870438	8027	8039
GC_{38-15}	4523783	8039	8039
LC_{60-65}	7387229	8039	8039
LC_{91-60}	8935664	8039	8039
BC_{12-17}^{ER}	2649823	8027	8039
BC_{34-27}^{ER}	6630077	8038	8039

Supplementary Table S2. Concordance of DR gene pairs identified from datasets with the same control samples but different case samples

Dataset	#DR pair	#Overlapped pair	#Concordant pair	Concordant ratio
GC_{38-15}	4523783			
GC_{12-15}	249379	193347	192881	0.9976
GC_{12-31}	3870438			
GC_{38-31}	3060133	1275627	1259675	0.9875
LC_{91-60}	8935664			
LC_{60-60}	5035285	4025404	3943116	0.9796
LC_{60-65}	7387229			
LC_{91-65}	7977878	4811059	4669631	0.9706
BC_{34-27}^{ER}	6630077			
BC_{12-27}^{ER}	2527003	2114920	2111197	0.9982
BC_{12-17}^{ER}	2649823			
BC_{34-17}^{ER}	3087813	1366180	1363250	0.9979

Supplementary Table S3. Information of normal samples for lung

Dataset[a]	Ethnicity	Age	Gender	Paired sample
LC_{91-65}	Caucasian	/	M:F ： U*=41:11: 13	All paired
LC_{60-60}	Chinese	/	F=60	All paired

*M denotes male, F denotes female, U denotes unknown.

Supplementary Table S4. Performance of *DRFunc* for DR gene pairs with different extents of noise

Proportions of random gene pairs	5%	10%	15%	20%
GC_{38-31} （239）*				
Random Detection#1	237.9	233.4	226.7	224.5
Overlaps#2	232.7	227.8	222.2	219.2
Reproducibility ratio	0.9781	0.976	0.9801	0.9764
GC_{12-15} （73）				
Random Detection	73	70.3	69.2	67.9
Overlaps	71.1	69	67.7	66.5
Reproducibility ratio	0.974	0.9815	0.9783	0.9794
BC12-27 （363）				
Random Detection	351.6	342.9	337.2	331.5
Overlaps	346.4	337.5	330.4	324.6
Reproducibility ratio	0.9852	0.9843	0.9798	0.9792
BC_{34-17} （366）				
Random Detection	358.9	353.2	348.9	339.9
Overlaps	350.9	344.5	340	331.2
Reproducibility ratio	0.9777	0.9754	0.9745	0.9744
LC_{91-65} (380)				
Random Detection	369.9	362.4	354.2	347
Overlaps	379.5	373.1	366.7	359.2
Reproducibility ratio	0.9747	0.9713	0.9659	0.966
LC_{60-60} (255)				
Random Detection	253.1	248.8	244.9	238.9
Overlaps	247.2	241.3	237.7	231.6
Reproducibility ratio	0.9767	0.9699	0.9706	0.9694

[*]The number inside the parentheses indicates the number of significant pathways enriched for the real DR gene pairs identified from the dataset indicated outside the parentheses. [#1]denotes the average number of pathways detected by the DR gene pairs with random gene pairs in the random experiments. [#2]denotes the average overlaps between the significant pathways detected by the DR gene pairs with random gene pairs and the significant pathways detected by the real DR gene pairs without random gene pairs.

Pathways enriched in at least one cancer dataset

Pathway Name	$BC_{34\text{-}17}$	$BC_{12\text{-}27}$	$LC_{91\text{-}65}$	$LC_{60\text{-}60}$	$GC_{38\text{-}31}$	$GC_{12\text{-}15}$
KEGG ECM RECEPTOR INTERACTION	0.000	0.000	0.000	0.000	0.000	0.000
KEGG FOCAL ADHESION	0.000	0.000	0.000	0.000	1.39E-06	0.000
	8.84E-12	3.56E-14	3.39E-06	0.000	1.06E-09	1.22E-07
KEGG LEUKOCYTE TRANSENDOTHELIAL MIGRATION						
		1.04E-13		0.000		
KEGG TGF BETA SIGNALING PATHWAY	0.000		0.000		1.43E-09	1.23E-10
			0.000			0.000
NABA CORE MATRISOME	0.000	0.000		0.000	0.000	
	0.000					
NABA ECM GLYCOPROTEINS						
		0.000	0.000	0.000	0.000	0.000
	0.000	0.000	0.000	0.000		
NABA MATRISOME						
					0.000	0.000
	0.000					
PID AVB3 INTEGRIN PATHWAY						
		0.000	7.66E-07	0.000	0.000	0.000
	0.000	1.09E-14	0.000	0.000		
PID INTEGRIN1 PATHWAY						
					0.000	0.000
	3.65E-09					
PID INTEGRIN A9B1 PATHWAY						
		0.000277152	1.34E-07	4.66E-08	1.14E-13	0.000756801
	0.000328418	2.56E-08	0.000	5.19E-06		
PID RB 1PATHWAY						
					4.48E-13	0.01440988
PID SYNDECAN 1 PATHWAY	4.15E-08	8.33E-10	1.27E-09	0.000	0.000	0.000

REACTOME CELL CELL COMMUNICATION	0.000	0.025982092	0.020868885	0.000	8.09E-05	0.000
REACTOME COLLAGEN FORMATION	0.020673888	0.004630379	0.0006244	1.49E-07	1.18E-05	0.000
REACTOME EXTRACELLULAR MATRIX ORGANIZATION	0.000	0.000	0.000	0.000	0.000	0.000
REACTOME GLYCOSAMINOGLYCAN METABOLISM	0.000	0.000	0.000	0.000	5.66E-07	8.19E-07
REACTOME METABOLISM OF CARBOHYDRATES	0.000	0.000	0.042950203	0.000	0.000	0.000215048
BIOCARTA AKAP95 PATHWAY	0.002030526	3.59E-06	0.000959404	0.005048181	2.16E-07	>0.05
BIOCARTA CARDIACEGF PATHWAY	6.86E-06	6.75E-06	6.54E-09	3.15E-09	>0.05	0.025046237
BIOCARTA CELLCYCLE PATHWAY	0.000875737	4.13E-06	1.06E-06	1.83E-07	2.20E-06	>0.05
BIOCARTA FAS PATHWAY	0.000743123	0.001741416	0.000	0.00113374	9.37E-10	>0.05
BIOCARTA G2 PATHWAY	0.034916859	1.21E-08	6.64E-07	0.023794291	5.04E-07	>0.05
KEGG ALPHA LINOLENIC ACID METABOLISM	0.00181339	0.030254922	1.00E-07	0.015012914	0.012102704	>0.05
KEGG BLADDER CANCER	0.000	0.000	0.000	0.000	0.000	>0.05
KEGG CELL CYCLE	0.000	0.000	0.000	0.000	0.000	>0.05
KEGG DNA REPLICATION	1.28E-08	0.000	0.000	0.008128227	1.58E-10	>0.05

KEGG DRUG METABOLISM CYTOCHROME P450	9.83E-10	>0.05	0.000111804	7.41E-08	0.000	0.003424051
KEGG ETHER LIPID METABOLISM	2.14E-09	0.000177036	6.15E-05	0.000583919	7.67E-14	>0.05
KEGG GLUTATHIONE METABOLISM	3.93E-12	2.54E-09	7.41E-12	0.029168171	2.57E-08	>0.05
KEGG P53 SIGNALING PATHWAY	3.18E-08	0.000	0.000	0.000	0.000	>0.05
KEGG PATHWAYS IN CANCER	0.000	0.000	0.000	0.000	9.54E-09	>0.05
KEGG TYROSINE METABOLISM	0.000	0.000	0.000715436	0.040553279	1.40E-11	>0.05
KEGG VASCULAR SMOOTH MUSCLE CONTRACTION	0.000248425	>0.05	0.000	5.56E-09	0.001790786	0.003832122
KEGG WNT SIGNALING PATHWAY	4.41E-15	>0.05	0.000	2.57E-12	3.83E-11	8.63E-06
NABA ECM REGULATORS	>0.05	5.79E-06	1.56E-08	0.000	0.000	0.000
NABA MATRISOME ASSOCIATED	>0.05	9.99E-09	0.000	0.000	1.18E-14	0.000
NABA PROTEOGLYCANS	0.045485658	0.000611312	>0.05	0.024839274	0.004507223	0.000174579
PID ALK1 PATHWAY	>0.05	0.000837332	6.43E-05	0.016318196	0.009533033	0.021532908
PID AP1 PATHWAY	1.96E-09	1.16E-08	0.000	0.000	0.000674533	>0.05
PID ARF6 TRAFFICKING PATHWAY	0.000	5.75E-15	2.10E-05	7.21E-10	0.000745249	>0.05

PID AURORA A PATHWAY	5.60E-10	0.000	1.30E-07	4.62E-07	6.71E-12	>0.05
PID AURORA B PATHWAY	0.000	0.000	0.000	0.000	0.000	>0.05
PID E2F PATHWAY	0.000	0.000	0.000	0.000	0.000	>0.05
PID ENDOTHELIN PATHWAY	2.48E-06	2.81E-09	6.88E-14	0.001794742	0.001831876	>0.05
PID FOXM1 PATHWAY	0.000	0.000	0.000	0.000	0.000	>0.05
PID FRA PATHWAY	3.28E-06	3.42E-06	0.000	0.000	1.58E-10	>0.05
PID HIF1 TFPATHWAY	9.49E-09	9.89E-08	0.005537308	1.19E-08	>0.05	0.000598418
PID INTEGRIN3 PATHWAY	0.000933796	>0.05	2.20E-06	0.000111447	1.50E-07	8.19E-07
PID LYMPH ANGIOGENESIS PATHWAY	0.000	2.44E-11	0.000381283	2.50E-11	>0.05	0.003418997
PID MYC ACTIV PATHWAY	2.44E-06	0.000	1.85E-08	0.000	1.31E-07	>0.05
PID P53 DOWNSTREAM PATHWAY	0.000	0.000	0.000	0.000	0.000	>0.05
PID P73PATHWAY	0.026615868	0.000	0.000	6.03E-15	0.000	>0.05
PID PLK1 PATHWAY	0.000	0.000	0.000	0.000	0.000	>0.05
PID SYNDECAN 4 PATHWAY	5.14E-12	0.000122156	0.025955317	0.021613924	0.003777135	>0.05

PID UPA UPAR PATHWAY	0.007795711	3.00E-10	0.000	2.95E-11	0.000	>0.05
PID WNT NONCANONICAL PATHWAY	1.55E-09	0.007341936	4.92E-14	0.005711317	0.000196687	>0.05
REACTOME APC CDC20 MEDIATED DEGRADATION OF NEK2A	4.50E-12	0.000	5.09E-06	9.79E-10	2.32E-14	>0.05
REACTOME APC C CDC20 MEDIATED DEGRADATION OF CYCLIN	7.57E-11	2.34E-13	5.85E-05	3.91E-08	3.09E-09	>0.05
REACTOME BIOLOGICAL OXIDATIONS	0.000	0.010660459	7.28E-08	0.00484655	0.000	>0.05
REACTOME CELL CYCLE	0.000	0.000	0.000	0.000	0.000	>0.05
REACTOME CELL CYCLE MITOTIC	0.000	0.000	0.000	0.000	0.000	>0.05
REACTOME CELL JUNCTION ORGANIZATION	3.89E-11	>0.05	6.41E-07	0.000	2.35E-09	0.000
REACTOME CHEMOKINE RECEPTORS BIND CHEMOKINES	0.010306388	0.000	3.88E-10	0.000	0.000	>0.05
REACTOME DEGRADATION OF THE EXTRACELLULAR MATRIX	9.59E-12	0.000	1.09E-09	8.50E-09	2.44E-06	>0.05
REACTOME DNA REPLICATION	0.000	0.000	0.000	2.32E-14	0.000	>0.05
REACTOME E2F MEDIATED REGULATION OF DNA REPLICATION	3.66E-12	0.000	0.000	3.11E-13	8.07E-09	>0.05
REACTOME FACTORS INVOLVED IN MEGAKARYOCYTE DEVELOPMENT AND PLATELET PRODUCTION	0.000	0.000	7.37E-10	4.34E-07	6.38E-08	>0.05
REACTOME G1 S SPECIFIC TRANSCRIPTION	0.038357016	0.000	6.56E-11	5.58E-05	0.026495998	>0.05

REACTOME G2 M CHECKPOINTS	2.20E-10	0.000	0.000	0.000634589	0.000	>0.05
REACTOME G2 M DNA DAMAGE CHECKPOINT	0.02291813	7.41E-05	0.000391276	0.004249797	0.000122516	>0.05
REACTOME HYALURO>0.05 METABOLISM	5.37E-07	1.12E-05	6.84E-12	8.05E-09	0.000763171	>0.05
REACTOME HYALURO>0.05 UPTAKE AND DEGRADATION	0.012372435	6.85E-06	0.0006244	0.000155027	0.000371481	>0.05
REACTOME INHIBITION OF THE PROTEOLYTIC ACTIVITY OF APC C REQUIRED FOR THE ONSET OF ANAPHASE BY MITOTIC SPINDLE CHECKPOINT COMPO..."'	1.28E-14	0.000	4.42E-08	7.05E-13	1.18E-14	>0.05
REACTOME INTEGRIN CELL SURFACE INTERACTIONS	0.000	>0.05	0.000	0.000	1.58E-13	0.000
REACTOME KINESINS	1.89E-12	1.08E-13	3.14E-12	6.64E-11	0.000	>0.05
REACTOME MITOTIC G1 G1 S PHASES	0.000	0.000	5.61E-15	0.006350529	7.04E-07	>0.05
REACTOME MITOTIC M M G1 PHASES	0.000	0.000	0.000	0.017229387	0.000	>0.05
REACTOME MITOTIC PROMETAPHASE	0.000	0.000	0.000	0.000	0.000	>0.05
REACTOME NUCLEAR RECEPTOR TRANSCRIPTION PATHWAY	>0.05	0.039132913	0.001017743	0.020145807	0.033436458	1.92E-08
REACTOME PHASE1 FUNCTIONALIZATION OF COMPOUNDS	0.000	0.000783981	0.000	4.50E-07	0.000	>0.05
REACTOME PHOSPHORYLATION OF THE APC C	2.76E-05	1.58E-10	0.001535769	9.29E-06	4.13E-07	>0.05
REACTOME SPHINGOLIPID DE NOVO BIOSYNTHESIS	0.000316274	0.003133761	4.35E-08	8.80E-07	>0.05	0.008516302

BIOCARTA AT1R PATHWAY	0.034683762	>0.05	0.000216382	0.001288041	>0.05	0.012313205
BIOCARTA G1 PATHWAY	>0.05	0.001150573	0.000	4.18E-09	0.000457929	>0.05
BIOCARTA LAIR PATHWAY	0.010646281	4.31E-05	>0.05	0.00029244	3.09E-09	>0.05
BIOCARTA PPARA PATHWAY	1.26E-09	>0.05	0.038661065	0.010503529	1.99E-06	>0.05
BIOCARTA PTC1 PATHWAY	0.00777392	0.004775213	0.015528269	>0.05	3.58E-06	>0.05
BIOCARTA STATHMIN PATHWAY	5.36E-06	3.80E-08	>0.05	4.53E-05	3.91E-05	>0.05
BIOCARTA TNFR1 PATHWAY	0.000316274	0.000329062	3.43E-06	>0.05	1.99E-06	>0.05
KEGG ABC TRANSPORTERS	2.00E-12	0.000	8.65E-06	5.36E-06	>0.05	>0.05
KEGG ADHERENS JUNCTION	0.000	8.92E-11	2.86E-13	2.23E-07	>0.05	>0.05
KEGG ALANINE ASPARTATE AND GLUTAMATE METABOLISM	>0.05	0.036026619	3.33E-08	0.001288041	1.11E-05	>0.05
KEGG AXON GUIDANCE	8.66E-08	0.000104579	0.000	0.000	>0.05	>0.05
KEGG BASAL CELL CARCINOMA	1.49E-05	>0.05	0.000	1.70E-09	4.86E-07	>0.05
KEGG CELL ADHESION MOLECULES CAMS	6.18E-05	>0.05	0.000	5.38E-10	>0.05	1.38E-06
KEGG CHEMOKINE SIGNALING PATHWAY	>0.05	7.38E-15	0.000	0.000	8.02E-11	>0.05

KEGG CYTOKINE CYTOKINE RECEPTOR INTERACTION	>0.05	2.86E-07	0.000311311	0.000	0.000	>0.05
KEGG DILATED CARDIOMYOPATHY	0.000	0.028995714	0.000	3.88E-13	>0.05	>0.05
KEGG FC GAMMA R MEDIATED PHAGOCYTOSIS	0.000	4.00E-06	0.000	0.006714911	>0.05	>0.05
KEGG FRUCTOSE AND MANNOSE METABOLISM	0.000109511	>0.05	1.28E-06	6.78E-05	0.004715604	>0.05
KEGG GLYCEROPHOSPHOLIPID METABOLISM	0.000	0.015289937	0.001477289	>0.05	5.66E-07	>0.05
KEGG GLYCOLYSIS GLUCONEOGENESIS	3.84E-11	5.30E-07	>0.05	>0.05	5.53E-10	0.007282715
KEGG HISTIDINE METABOLISM	0.033845909	>0.05	0.017015885	0.004895952	>0.05	0.026920485
KEGG MELANOGENESIS	3.05E-11	0.016281688	0.000	>0.05	0.000	>0.05
KEGG MTOR SIGNALING PATHWAY	0.000	0.000	0.000139214	0.00207357	>0.05	>0.05
KEGG NITROGEN METABOLISM	1.10E-05	0.007001031	1.06E-06	0.005419428	>0.05	>0.05
KEGG PANCREATIC CANCER	1.96E-09	0.000	0.000	6.78E-05	>0.05	>0.05
KEGG PROGESTERONE MEDIATED OOCYTE MATURATION	0.000	0.000	2.70E-05	>0.05	0.000	>0.05
KEGG PURINE METABOLISM	0.036228571	2.79E-06	>0.05	0.016861318	9.11E-05	>0.05
KEGG PYRIMIDINE METABOLISM	2.29E-12	0.000	2.81E-14	>0.05	0.007240631	>0.05

KEGG RENAL CELL CARCINOMA	2.38E-11	5.36E-08	0.000	2.84E-14	>0.05	>0.05
KEGG RENIN ANGIOTENSIN SYSTEM	3.89E-07	4.75E-07	2.93E-07	4.42E-12	>0.05	>0.05
KEGG SPHINGOLIPID METABOLISM	0.01253275	>0.05	0.024089694	8.40E-06	0.000189793	>0.05
NABA COLLAGENS	>0.05	>0.05	5.49E-11	0.000	3.37E-08	0.000
PID AMB2 NEUTROPHILS PATHWAY	1.00E-05	8.07E-08	9.48E-12	0.000	>0.05	>0.05
PID ANGIOPOIETIN RECEPTOR PATHWAY	0.000	0.000	0.000155189	7.17E-12	>0.05	>0.05
PID ARF6 PATHWAY	6.38E-08	1.84E-10	2.19E-09	0.005419428	>0.05	>0.05
PID CASPASE PATHWAY	0.000	2.91E-11	2.20E-05	1.56E-05	>0.05	>0.05
PID CMYB PATHWAY	0.001508862	0.031769994	3.34E-06	>0.05	5.73E-12	>0.05
PID CXCR4 PATHWAY	0.000	0.000	3.79E-15	3.46E-09	>0.05	>0.05
PID FANCONI PATHWAY	3.37E-08	7.52E-10	2.91E-07	>0.05	0.000	>0.05
PID FCER1 PATHWAY	1.46E-09	0.005286721	0.000	2.33E-06	>0.05	>0.05
PID FGF PATHWAY	0.000	0.000	2.97E-12	2.15E-06	>0.05	>0.05
PID ILK PATHWAY	3.18E-08	2.75E-10	0.000	5.61E-09	>0.05	>0.05

PID INTEGRIN A4B1 PATHWAY	0.030012518	>0.05	>0.05	0.000569841	0.000	0.002167107
PID INTEGRIN CS PATHWAY	4.78E-07	0.022030661	0.000683351	0.016318196	>0.05	>0.05
PID LYSOPHOSPHOLIPID PATHWAY	9.01E-08	0.004825715	0.001564871	>0.05	9.88E-06	>0.05
PID MYC PATHWAY	0.004789185	>0.05	2.43E-13	2.12E-07	0.015632857	>0.05
PID SHP2 PATHWAY	0.000	0.000	8.02E-13	0.000115071	>0.05	>0.05
PID VEGF VEGFR PATHWAY	5.25E-07	6.85E-06	0.012169209	2.10E-06	>0.05	>0.05
REACTOME ABC FAMILY PROTEINS MEDIATED TRANSPORT	2.27E-12	4.74E-11	1.85E-05	0.000234475	>0.05	>0.05
REACTOME ACTIVATION OF ATR IN RESPONSE TO REPLICATION STRESS	2.41E-05	3.89E-15	3.80E-09	>0.05	5.65E-08	>0.05
REACTOME ACTIVATION OF THE PRE REPLICATIVE COMPLEX	8.09E-05	2.27E-13	1.24E-05	>0.05	0.000151148	>0.05
REACTOME ACYL CHAIN REMODELLING OF PC	0.000236404	0.005834429	0.018286656	>0.05	0.000548105	>0.05
REACTOME ACYL CHAIN REMODELLING OF PE	0.002218978	0.005063928	0.001186968	>0.05	0.016832491	>0.05
REACTOME ACYL CHAIN REMODELLING OF PI	0.039807053	0.014176044	0.01246617	>0.05	0.012102704	>0.05
REACTOME ACYL CHAIN REMODELLING OF PS	0.020190808	0.030254922	0.01246617	>0.05	0.012102704	>0.05
REACTOME CD28 DEPENDENT PI3K AKT SIGNALING	0.00777392	4.42E-07	3.50E-10	1.33E-10	>0.05	>0.05

REACTOME CELL CELL JUNCTION ORGANIZATION	9.83E-09	>0.05	>0.05	2.55E-11	1.06E-08	0.000
REACTOME CELL CYCLE CHECKPOINTS	1.90E-05	0.000	5.94E-05	>0.05	0.000	>0.05
REACTOME CELL SURFACE INTERACTIONS AT THE VASCULAR WALL	0.000	0.000	0.000	0.000	>0.05	>0.05
REACTOME CHONDROITIN SULFATE DERMATAN SULFATE METABOLISM	1.09E-14	0.000227309	>0.05	2.07E-05	>0.05	4.74E-11
REACTOME CONVERSION FROM APC C CDC20 TO APC C CDH1 IN LATE ANAPHASE	5.66E-06	4.00E-06	>0.05	0.004237923	0.025745741	>0.05
REACTOME DEVELOPMENTAL BIOLOGY	0.000	0.000	0.009677638	>0.05	>0.05	0.017737613
REACTOME DIABETES PATHWAYS	9.41E-10	0.013747129	4.46E-05	>0.05	2.10E-07	>0.05
REACTOME DNA STRAND ELONGATION	3.62E-06	3.92E-07	7.28E-08	>0.05	1.30E-05	>0.05
REACTOME FATTY ACID TRIACYLGLYCEROL AND KETONE BODY METABOLISM	0.000	0.000	0.000	>0.05	2.38E-13	>0.05
REACTOME FGFR LIGAND BINDING AND ACTIVATION	7.73E-07	4.04E-08	7.87E-06	2.02E-07	>0.05	>0.05
REACTOME G0 AND EARLY G1	>0.05	4.80E-10	2.08E-06	0.021377897	0.000250787	>0.05
REACTOME G1 PHASE	0.017902818	0.001575435	0.0006244	0.005419428	>0.05	>0.05
REACTOME G1 S TRANSITION	6.59E-08	0.000	0.000129643	>0.05	0.002508199	>0.05
REACTOME GLYCEROPHOSPHOLIPID BIOSYNTHESIS	0.000	9.91E-09	3.90E-11	>0.05	6.28E-15	>0.05

REACTOME HEMOSTASIS	0.000	0.000	0.000	0.000	>0.05	>0.05
REACTOME KERATAN SULFATE BIOSYNTHESIS	0.000284421	2.67E-05	0.000683351	2.49E-05	>0.05	>0.05
REACTOME METABOLISM OF LIPIDS AND LIPOPROTEINS	0.000	0.000	8.52E-09	>0.05	2.08E-13	>0.05
REACTOME METABOLISM OF NUCLEOTIDES	0.000	0.000	1.17E-07	>0.05	6.28E-15	>0.05
REACTOME METABOLISM OF VITAMINS AND COFACTORS	3.59E-14	0.001171156	1.75E-07	4.03E-05	>0.05	>0.05
REACTOME OTHER SEMAPHORIN INTERACTIONS	>0.05	0.006212054	0.000194031	4.99E-05	0.012102704	>0.05
REACTOME O LINKED GLYCOSYLATION OF MUCINS	9.83E-09	>0.05	7.13E-13	0.000	>0.05	0.007515997
REACTOME PHOSPHOLIPID METABOLISM	0.000	0.002420192	4.27E-12	>0.05	1.18E-14	>0.05
REACTOME PLATELET SENSITIZATION BY LDL	1.83E-06	1.24E-05	1.22E-12	0.008128227	>0.05	>0.05
REACTOME PPARA ACTIVATES GENE EXPRESSION	0.000	0.000384825	2.90E-06	>0.05	1.49E-08	>0.05
REACTOME RECRUITMENT OF NUMA TO MITOTIC CENTROSOMES	1.47E-05	6.85E-06	>0.05	4.02E-05	3.40E-06	>0.05
REACTOME REGULATION OF SIGNALING BY CBL	2.99E-07	0.000340808	2.65E-07	0.001946999	>0.05	>0.05
REACTOME REVERSIBLE HYDRATION OF CARBON DIOXIDE	1.48E-05	0.000819292	0.000381283	7.45E-06	>0.05	>0.05
REACTOME SIGNALING BY ILS	0.001455501	2.52E-08	0.000	1.49E-10	>0.05	>0.05

REACTOME SIGNALING BY RHO GTPASES	0.000	0.020022942	0.000	0.000	>0.05	>0.05
REACTOME SIGNALING BY SCF KIT	2.10E-11	0.000	0.000	4.27E-06	>0.05	>0.05
REACTOME SMOOTH MUSCLE CONTRACTION	0.000875737	0.007001031	0.039274547	0.021377897	>0.05	>0.05
REACTOME SYNTHESIS AND INTERCONVERSION OF NUCLEOTIDE DI AND TRIPHOSPHATES	1.18E-06	4.75E-07	4.21E-06	0.000637351	>0.05	>0.05
REACTOME SYNTHESIS OF PA	1.27E-12	2.48E-05	0.001293318	>0.05	7.05E-07	>0.05
REACTOME TIE2 SIGNALING	0.010646281	0.037464138	0.000209653	9.29E-06	>0.05	>0.05
REACTOME TRANSCRIPTIONAL ACTIVITY OF SMAD2 SMAD3 SMAD4 HETEROTRIMER	0.008627187	0.038355334	0.004644968	>0.05	0.000332346	>0.05
REACTOME TRANSCRIPTIONAL REGULATION OF WHITE ADIPOCYTE DIFFERENTIATION	0.000	0.000	0.000	8.10E-08	>0.05	>0.05
REACTOME TRIGLYCERIDE BIOSYNTHESIS	8.41E-11	0.000791296	7.08E-06	>0.05	4.05E-05	>0.05
REACTOME VEGF LIGAND RECEPTOR INTERACTIONS	6.95E-05	3.58E-05	0.012169209	2.10E-06	>0.05	>0.05
SIG CHEMOTAXIS	3.18E-08	0.000114689	0.000	4.18E-14	>0.05	>0.05
ST ADRENERGIC	0.002774449	1.54E-07	1.97E-06	0.004042404	>0.05	>0.05
ST INTEGRIN SIGNALING PATHWAY	0.000	7.30E-14	3.88E-10	0.000162403	>0.05	>0.05
WNT SIGNALING	>0.05	>0.05	0.000	0.001458786	0.005830291	1.89E-07

BIOCARTA BCELLSURVIVAL PATHWAY	0.000132156	0.000846407	0.001535769	>0.05	>0.05	>0.05
BIOCARTA CASPASE PATHWAY	0.015469579	>0.05	0.000133674	5.73E-05	>0.05	>0.05
BIOCARTA CFTR PATHWAY	0.014552998	0.00762174	0.022150343	>0.05	>0.05	>0.05
BIOCARTA CXCR4 PATHWAY	0.000445319	0.024242725	>0.05	>0.05	0.003197352	>0.05
BIOCARTA EGFR SMRTE PATHWAY	0.000229128	9.64E-05	>0.05	0.000342063	>0.05	>0.05
BIOCARTA EIF4 PATHWAY	0.000859496	0.000357835	0.044088207	>0.05	>0.05	>0.05
BIOCARTA FIBRINOLYSIS PATHWAY	>0.05	>0.05	1.14E-07	0.002022599	0.000248063	>0.05
BIOCARTA HIVNEF PATHWAY	0.000140537	0.045342531	>0.05	>0.05	3.35E-06	>0.05
BIOCARTA IL2RB PATHWAY	0.04222716	0.018289308	0.001512668	>0.05	>0.05	>0.05
BIOCARTA KERATINOCYTE PATHWAY	0.047603346	>0.05	2.87E-05	0.00484655	>0.05	>0.05
BIOCARTA MONOCYTE PATHWAY	>0.05	0.037206948	3.75E-06	0.000342063	>0.05	>0.05
BIOCARTA SPPA PATHWAY	5.20E-05	1.33E-05	>0.05	>0.05	5.72E-05	>0.05
BIOCARTA SRCRPTP PATHWAY	>0.05	0.013999293	0.031991684	>0.05	1.68E-05	>0.05
KEGG ACUTE MYELOID LEUKEMIA	6.56E-06	8.26E-12	4.43E-08	>0.05	>0.05	>0.05

KEGG ALDOSTERONE REGULATED SODIUM REABSORPTION	1.67E-07	3.12E-07	>0.05	>0.05	2.34E-07	>0.05
KEGG ARACHIDONIC ACID METABOLISM	>0.05	>0.05	1.98E-11	2.43E-06	0.000	>0.05
KEGG ARGININE AND PROLINE METABOLISM	>0.05	0.000902083	>0.05	>0.05	0.009289215	3.90E-06
KEGG COLORECTAL CANCER	0.013586649	0.000	1.01E-06	>0.05	>0.05	>0.05
KEGG FATTY ACID METABOLISM	5.24E-08	7.66E-06	>0.05	>0.05	0.02542856	>0.05
KEGG FC EPSILON RI SIGNALING PATHWAY	1.00E-05	>0.05	0.000	1.80E-05	>0.05	>0.05
KEGG GLYCEROLIPID METABOLISM	0.022785879	>0.05	>0.05	>0.05	4.90E-06	2.06E-06
KEGG GLYCINE SERINE AND THREONINE METABOLISM	1.16E-05	0.037218646	>0.05	0.006542575	>0.05	>0.05
KEGG HEDGEHOG SIGNALING PATHWAY	>0.05	>0.05	8.32E-08	1.05E-05	7.14E-13	>0.05
KEGG HYPERTROPHIC CARDIOMYOPATHY HCM	0.000	>0.05	0.000	8.75E-14	>0.05	>0.05
KEGG LINOLEIC ACID METABOLISM	>0.05	>0.05	5.02E-07	8.02E-10	1.40E-09	>0.05
KEGG MELANOMA	4.76E-06	>0.05	0.016992565	0.008347617	>0.05	>0.05
KEGG MISMATCH REPAIR	>0.05	0.010643388	0.001334047	>0.05	5.72E-05	>0.05
KEGG NICOTINATE AND NICOTINAMIDE METABOLISM	0.022199429	0.008545396	>0.05	0.015981185	>0.05	>0.05

KEGG ONE CARBON POOL BY FOLATE	0.002457247	4.31E-05	>0.05	>0.05	0.026495998	>0.05
KEGG OOCYTE MEIOSIS	1.91E-11	0.000	>0.05	>0.05	0.000	>0.05
KEGG O GLYCAN BIOSYNTHESIS	4.66E-10	>0.05	0.009339687	1.27E-10	>0.05	>0.05
KEGG PPAR SIGNALING PATHWAY	0.000	0.000	0.001796023	>0.05	>0.05	>0.05
KEGG PROSTATE CANCER	1.77E-10	1.08E-12	0.0200565	>0.05	>0.05	>0.05
KEGG RETINOL METABOLISM	0.006335422	>0.05	>0.05	0.029168171	0.000	>0.05
KEGG SMALL CELL LUNG CANCER	1.76E-10	0.001329674	9.68E-10	>0.05	>0.05	>0.05
KEGG SNARE INTERACTIONS IN VESICULAR TRANSPORT	7.76E-07	>0.05	7.62E-05	4.32E-06	>0.05	>0.05
KEGG THYROID CANCER	0.001589944	0.044697504	0.009278378	>0.05	>0.05	>0.05
KEGG TIGHT JUNCTION	0.000	>0.05	>0.05	0.004500921	>0.05	0.000115586
KEGG TRYPTOPHAN METABOLISM	>0.05	7.55E-14	0.001194626	0.03284268	>0.05	>0.05
KEGG VEGF SIGNALING PATHWAY	0.000596356	>0.05	7.53E-09	1.35E-05	>0.05	>0.05
NABA SECRETED FACTORS	>0.05	1.26E-08	0.000	0.000	>0.05	>0.05
PID AJDISS 2PATHWAY	1.68E-13	>0.05	0.000328754	>0.05	6.35E-05	>0.05

PID ATF2 PATHWAY	>0.05	>0.05	1.04E-05	1.08E-06	0.000433918	>0.05
PID ATR PATHWAY	>0.05	0.000169589	0.012865799	>0.05	2.63E-10	>0.05
PID AVB3 OPN PATHWAY	1.25E-09	8.35E-06	>0.05	0.025193939	>0.05	>0.05
PID BMP PATHWAY	>0.05	>0.05	0.000	0.000	0.002098056	>0.05
PID CXCR3 PATHWAY	0.000	0.000	4.64E-14	>0.05	>0.05	>0.05
PID ECADHERIN STABILIZATION PATHWAY	2.66E-12	0.020038406	0.001565905	>0.05	>0.05	>0.05
PID ERBB1 RECEPTOR PROXIMAL PATHWAY	2.11E-09	8.50E-11	0.030458439	>0.05	>0.05	>0.05
PID GLYPICAN 1PATHWAY	0.002844757	0.008291526	0.007478591	>0.05	>0.05	>0.05
PID HES HEY PATHWAY	>0.05	0.0001303	4.71E-08	1.82E-05	>0.05	>0.05
PID HIF2PATHWAY	>0.05	0.002974385	2.44E-11	9.79E-10	>0.05	>0.05
PID IL2 1PATHWAY	0.002531182	0.000	3.78E-06	>0.05	>0.05	>0.05
PID IL2 STAT5 PATHWAY	0.00777392	8.20E-07	0.000598603	>0.05	>0.05	>0.05
PID INTEGRIN2 PATHWAY	>0.05	>0.05	7.63E-14	0.021377897	0.040670878	>0.05
PID MYC REPRESS PATHWAY	>0.05	0.002379323	0.004644968	>0.05	0.000280321	>0.05

PID NOTCH PATHWAY	>0.05	>0.05	3.22E-08	>0.05	1.16E-11	4.86E-06
PID P38 ALPHA BETA DOWNSTREAM PATHWAY	0.006622823	>0.05	0.007509646	>0.05	1.68E-06	>0.05
PID REG GR PATHWAY	>0.05	>0.05	0.000	0.000143366	0.00810293	>0.05
PID S1P META PATHWAY	0.001138761	1.63E-10	1.30E-07	>0.05	>0.05	>0.05
PID S1P S1P1 PATHWAY	0.00777392	8.18E-06	1.94E-05	>0.05	>0.05	>0.05
PID S1P S1P2 PATHWAY	0.004943586	7.62E-06	2.35E-06	>0.05	>0.05	>0.05
PID TAP63 PATHWAY	>0.05	>0.05	0.000	0.000	0.000	>0.05
PID WNT CANONICAL PATHWAY	>0.05	>0.05	5.91E-11	0.043468799	>0.05	0.041673395
PID WNT SIGNALING PATHWAY	>0.05	>0.05	2.49E-14	2.24E-09	0.00066911	>0.05
REACTOME ACTIVATED TLR4 SIGNALLING	8.32E-14	0.000	0.000	>0.05	>0.05	>0.05
REACTOME ACYL CHAIN REMODELLING OF PG	0.009787131	0.045342531	>0.05	>0.05	0.000154559	>0.05
REACTOME ANTIVIRAL MECHANISM BY IFN STIMULATED GENE	0.022751964	>0.05	4.14E-05	>0.05	2.16E-07	>0.05
REACTOME APC C CDC20 MEDIATED DEGRADATION OF MITOTIC PROTEINS	6.03E-05	3.11E-13	>0.05	>0.05	0.012260961	>0.05
REACTOME APOPTOTIC CLEAVAGE OF CELLULAR PROTEINS	8.59E-10	>0.05	1.08E-11	0.004414191	>0.05	>0.05

REACTOME BASIGIN INTERACTIONS	5.89E-06	4.75E-14	0.023048906	>0.05	>0.05	>0.05
REACTOME BOTULINUM NEUROTOXICITY	0.005282408	>0.05	2.12E-10	3.73E-06	>0.05	>0.05
REACTOME CD28 CO STIMULATION	>0.05	4.38E-06	2.61E-08	5.43E-09	>0.05	>0.05
REACTOME CTLA4 INHIBITORY SIGNALING	0.022995986	0.001255563	0.026990725	>0.05	>0.05	>0.05
REACTOME CYCLIN A B1 ASSOCIATED EVENTS DURING G2 M TRANSITION	0.039807053	>0.05	0.021694406	>0.05	0.000113247	>0.05
REACTOME E2F ENABLED INHIBITION OF PRE REPLICATION COMPLEX FORMATION	>0.05	0.00075405	0.001788015	>0.05	0.016331761	>0.05
REACTOME ENOS ACTIVATION AND REGULATION	>0.05	0.008583626	0.006530026	0.000425173	>0.05	>0.05
REACTOME ERKS ARE INACTIVATED	0.034683762	0.020022942	0.011186079	>0.05	>0.05	>0.05
REACTOME ERK MAPK TARGETS	0.00777392	8.18E-06	6.15E-08	>0.05	>0.05	>0.05
REACTOME ETHANOL OXIDATION	0.012372435	0.009765637	>0.05	>0.05	0.001478262	>0.05
REACTOME GAB1 SIGNALOSOME	>0.05	0.020022942	0.013154539	1.08E-06	>0.05	>0.05
REACTOME GLUTATHIONE CONJUGATION	0.000561628	>0.05	0.00190082	>0.05	0.002573695	>0.05
REACTOME G ALPHA I SIGNALLING EVENTS	>0.05	0.000	>0.05	1.36E-06	6.44E-09	>0.05
REACTOME IL 3 5 AND GM CSF SIGNALING	0.037406308	0.0337138	8.65E-06	>0.05	>0.05	>0.05

REACTOME IL 6 SIGNALING	0.034683762	>0.05	0.004880697	0.005419428	>0.05	>0.05
REACTOME KERATAN SULFATE KERATIN METABOLISM	0.00777392	0.00018056	>0.05	0.011511761	>0.05	>0.05
REACTOME MAPK TARGETS NUCLEAR EVENTS MEDIATED BY MAP KINASES	6.86E-06	8.20E-07	1.28E-08	>0.05	>0.05	>0.05
REACTOME MAP KINASE ACTIVATION IN TLR CASCADE	4.64E-05	0.000	4.17E-14	>0.05	>0.05	>0.05
REACTOME MHC CLASS II ANTIGEN PRESENTATION	>0.05	0.037464138	1.09E-08	>0.05	0.000657611	>0.05
REACTOME MITOTIC G2 G2 M PHASES	0.022810956	5.72E-10	>0.05	>0.05	1.41E-10	>0.05
REACTOME MYD88 MAL CASCADE INITIATED ON PLASMA MEMBRANE	1.05E-05	9.30E-13	0.000	>0.05	>0.05	>0.05
REACTOME NFKB AND MAP KINASES ACTIVATION MEDIATED BY TLR4 SIGNALING REPERTOIRE	4.51E-08	2.13E-12	0.000	>0.05	>0.05	>0.05
REACTOME NUCLEAR EVENTS KINASE AND TRANSCRIPTION FACTOR ACTIVATION	0.002778705	8.39E-08	5.07E-09	>0.05	>0.05	>0.05
REACTOME PEPTIDE LIGAND BINDING RECEPTORS	>0.05	1.08E-13	>0.05	0.000	2.34E-07	>0.05
REACTOME PI 3K CASCADE	0.000176873	1.46E-10	>0.05	1.45E-05	>0.05	>0.05
REACTOME PLATELET ACTIVATION SIGNALING AND AGGREGATION	2.66E-09	3.42E-14	1.09E-14	>0.05	>0.05	>0.05
REACTOME PROTEOLYTIC CLEAVAGE OF SNARE COMPLEX PROTEINS	0.00430073	>0.05	2.24E-06	2.33E-06	>0.05	>0.05
REACTOME REGULATION OF MITOTIC CELL CYCLE	0.047739256	0.000	>0.05	>0.05	0.000162734	>0.05

REACTOME RESPONSE TO ELEVATED PLATELET CYTOSOLIC CA	>0.05	8.25E-06	7.38E-15	0.03143179	>0.05	>0.05
REACTOME SHC MEDIATED CASCADE	0.000132156	5.89E-06	>0.05	0.029168171	>0.05	>0.05
REACTOME SIGNALING BY PDGF	>0.05	>0.05	>0.05	5.63E-10	0.000	0.000
REACTOME SIGNALING BY TGF BETA RECEPTOR COMPLEX	9.44E-08	>0.05	9.86E-05	>0.05	0.002086165	>0.05
REACTOME TIGHT JUNCTION INTERACTIONS	>0.05	>0.05	>0.05	4.53E-10	1.42E-10	3.38E-11
REACTOME TOLL RECEPTOR CASCADES	3.44E-06	7.38E-15	0.000	>0.05	>0.05	>0.05
REACTOME TRAF6 MEDIATED INDUCTION OF NFKB AND MAP KINASES UPON TLR7 8 OR 9 ACTIVATION	6.88E-05	2.46E-12	0.000	>0.05	>0.05	>0.05
REACTOME TRANSPORT OF GLUCOSE AND OTHER SUGARS BILE SALTS AND ORGANIC ACIDS METAL IONS AND AMINE COMPOUNDS	>0.05	>0.05	1.79E-05	2.33E-06	0.010257014	>0.05
REACTOME TRIF MEDIATED TLR3 SIGNALING	8.66E-13	0.000	0.000	>0.05	>0.05	>0.05
ST FAS SIGNALING PATHWAY	>0.05	3.39E-05	0.002620407	>0.05	3.20E-12	>0.05
ST GRANULE CELL SURVIVAL PATHWAY	>0.05	5.15E-07	1.43E-06	1.38E-06	>0.05	>0.05
ST MYOCYTE AD PATHWAY	>0.05	0.00231852	1.10E-08	0.001184885	>0.05	>0.05
BIOCARTA ACE2 PATHWAY	>0.05	>0.05	6.52E-05	2.68E-06	>0.05	>0.05
BIOCARTA AGR PATHWAY	0.006099829	0.038355334	>0.05	>0.05	>0.05	>0.05

BIOCARTA AKAPCENTROSOME PATHWAY	>0.05	>0.05	0.036605118	>0.05	0.026263818	>0.05
BIOCARTA AKT PATHWAY	0.047739256	0.010643388	>0.05	>0.05	>0.05	>0.05
BIOCARTA ALK PATHWAY	>0.05	>0.05	0.000788454	0.002648737	>0.05	>0.05
BIOCARTA ARF PATHWAY	>0.05	>0.05	0.000100371	0.000128387	>0.05	>0.05
BIOCARTA ATRBRCA PATHWAY	>0.05	>0.05	0.005064547	>0.05	0.029212858	>0.05
BIOCARTA BIOPEPTIDES PATHWAY	>0.05	0.014945857	0.013173435	>0.05	>0.05	>0.05
BIOCARTA CCR5 PATHWAY	0.002457247	>0.05	0.008789742	>0.05	>0.05	>0.05
BIOCARTA CTCF PATHWAY	>0.05	>0.05	0.019788138	8.71E-07	>0.05	>0.05
BIOCARTA GH PATHWAY	0.03160415	2.80E-06	>0.05	>0.05	>0.05	>0.05
BIOCARTA GRANULOCYTES PATHWAY	>0.05	>0.05	0.033444199	>0.05	8.82E-05	>0.05
BIOCARTA IGF1 PATHWAY	4.38E-06	0.000594464	>0.05	>0.05	>0.05	>0.05
BIOCARTA IL3 PATHWAY	0.039807053	>0.05	0.001796023	>0.05	>0.05	>0.05
BIOCARTA IL7 PATHWAY	0.000191749	4.31E-05	>0.05	>0.05	>0.05	>0.05
BIOCARTA LEPTIN PATHWAY	0.00777392	9.64E-05	>0.05	>0.05	>0.05	>0.05

BIOCARTA LONGEVITY PATHWAY	3.31E-06	3.14E-09	>0.05	>0.05	>0.05	>0.05
BIOCARTA MCM PATHWAY	>0.05	0.00013499	0.03640926	>0.05	>0.05	>0.05
BIOCARTA MPR PATHWAY	0.019166836	0.003440915	>0.05	>0.05	>0.05	>0.05
BIOCARTA P38MAPK PATHWAY	>0.05	0.020994156	>0.05	>0.05	0.038597433	>0.05
BIOCARTA SET PATHWAY	>0.05	>0.05	0.006960476	0.001077033	>0.05	>0.05
BIOCARTA TFF PATHWAY	1.03E-05	0.005063928	>0.05	>0.05	>0.05	>0.05
KEGG ADIPOCYTOKINE SIGNALING PATHWAY	0.000	0.000	>0.05	>0.05	>0.05	>0.05
KEGG APOPTOSIS	>0.05	>0.05	0.001548907	0.039787547	>0.05	>0.05
KEGG ARRHYTHMOGENIC RIGHT VENTRICULAR CARDIOMYOPATHY ARVC	0.000	>0.05	8.28E-08	>0.05	>0.05	>0.05
KEGG CITRATE CYCLE TCA CYCLE	4.66E-10	8.15E-14	>0.05	>0.05	>0.05	>0.05
KEGG COMPLEMENT AND COAGULATION CASCADES	>0.05	>0.05	0.000	8.43E-07	>0.05	>0.05
KEGG ERBB SIGNALING PATHWAY	>0.05	>0.05	0.003567939	0.009338696	>0.05	>0.05
KEGG GLYCOSAMINOGLYCAN BIOSYNTHESIS CHONDROITIN SULFATE	>0.05	0.005834429	>0.05	0.004895952	>0.05	>0.05
KEGG GLYCOSAMINOGLYCAN BIOSYNTHESIS HEPARAN SULFATE	>0.05	>0.05	0.003527012	0.000723816	>0.05	>0.05

KEGG GNRH SIGNALING PATHWAY	0.000458345	>0.05	0.000	>0.05	>0.05	>0.05
KEGG INSULIN SIGNALING PATHWAY	0.000	0.000	>0.05	>0.05	>0.05	>0.05
KEGG METABOLISM OF XENOBIOTICS BY CYTOCHROME P450	2.55E-07	>0.05	>0.05	>0.05	0.000	>0.05
KEGG NEUROTROPHIN SIGNALING PATHWAY	0.018030022	0.026437366	>0.05	>0.05	>0.05	>0.05
KEGG NON SMALL CELL LUNG CANCER	>0.05	>0.05	0.001356572	0.002634136	>0.05	>0.05
KEGG PRION DISEASES	>0.05	>0.05	>0.05	0.032517946	0.040196489	>0.05
KEGG PROPANOATE METABOLISM	0.005288362	0.002974385	>0.05	>0.05	>0.05	>0.05
KEGG PROXIMAL TUBULE BICARBONATE RECLAMATION	1.55E-07	9.56E-06	>0.05	>0.05	>0.05	>0.05
KEGG PYRUVATE METABOLISM	3.05E-12	7.38E-15	>0.05	>0.05	>0.05	>0.05
KEGG STEROID BIOSYNTHESIS	0.019890934	0.000114419	>0.05	>0.05	>0.05	>0.05
KEGG TOLL LIKE RECEPTOR SIGNALING PATHWAY	2.36E-09	7.94E-07	>0.05	>0.05	>0.05	>0.05
KEGG TYPE II DIABETES MELLITUS	0.001756914	0.001371058	>0.05	>0.05	>0.05	>0.05
KEGG VALINE LEUCINE AND ISOLEUCINE DEGRADATION	2.41E-05	2.64E-05	>0.05	>0.05	>0.05	>0.05
KEGG VASOPRESSIN REGULATED WATER REABSORPTION	>0.05	>0.05	>0.05	>0.05	0.022609407	0.001871403

NABA BASEMENT MEMBRANES	>0.05	>0.05	>0.05	>0.05	0.000619727	0.02616183
NABA ECM AFFILIATED	>0.05	>0.05	0.000	0.000	>0.05	>0.05
PID AR NONGENOMIC PATHWAY	0.004091935	1.62E-05	>0.05	>0.05	>0.05	>0.05
PID BCR 5PATHWAY	>0.05	>0.05	2.49E-14	>0.05	0.005228606	>0.05
PID CD8 TCR PATHWAY	>0.05	>0.05	3.73E-05	0.015012914	>0.05	>0.05
PID CERAMIDE PATHWAY	0.00035673	>0.05	>0.05	>0.05	0.02542856	>0.05
PID CIRCADIAN PATHWAY	>0.05	>0.05	0.000427295	>0.05	3.85E-05	>0.05
PID DELTA NP63 PATHWAY	>0.05	>0.05	0.000	5.83E-05	>0.05	>0.05
PID EPHB FWD PATHWAY	0.000273245	0.003169331	>0.05	>0.05	>0.05	>0.05
PID ER NONGENOMIC PATHWAY	2.27E-13	2.34E-08	>0.05	>0.05	>0.05	>0.05
PID FAS PATHWAY	1.92E-06	0.025108099	>0.05	>0.05	>0.05	>0.05
PID FOXO PATHWAY	4.31E-08	0.012073053	>0.05	>0.05	>0.05	>0.05
PID HDAC CLASSIII PATHWAY	>0.05	0.001961091	>0.05	>0.05	0.001792848	>0.05
PID HEDGEHOG 2PATHWAY	>0.05	>0.05	5.29E-07	4.53E-06	>0.05	>0.05

PID HNF3A PATHWAY	>0.05	>0.05	1.64E-10	4.51E-07	>0.05	>0.05
PID IGF1 PATHWAY	5.24E-08	0.001741416	>0.05	>0.05	>0.05	>0.05
PID IL2 PI3K PATHWAY	0.036814686	0.035876497	>0.05	>0.05	>0.05	>0.05
PID IL6 7 PATHWAY	>0.05	>0.05	2.91E-07	0.023794291	>0.05	>0.05
PID IL8 CXCR2 PATHWAY	>0.05	>0.05	3.80E-05	0.003862363	>0.05	>0.05
PID INSULIN PATHWAY	0.000198518	1.98E-07	>0.05	>0.05	>0.05	>0.05
PID INTEGRIN5 PATHWAY	0.010646281	>0.05	>0.05	>0.05	0.001325679	>0.05
PID NEPHRIN NEPH1 PATHWAY	0.009469697	0.001741416	>0.05	>0.05	>0.05	>0.05
PID NFAT TFPATHWAY	>0.05	>0.05	2.66E-05	0.001288041	>0.05	>0.05
PID P38 GAMMA DELTA PATHWAY	0.00777392	0.037206948	>0.05	>0.05	>0.05	>0.05
PID P53 REGULATION PATHWAY	>0.05	>0.05	0.000455746	>0.05	3.45E-10	>0.05
PID PRL SIGNALING EVENTS PATHWAY	0.000875737	>0.05	0.039274547	>0.05	>0.05	>0.05
PID PTP1B PATHWAY	3.54E-05	2.44E-11	>0.05	>0.05	>0.05	>0.05
PID RHOA REG PATHWAY	1.13E-07	>0.05	0.007645644	>0.05	>0.05	>0.05

PID RXR VDR PATHWAY	6.97E-09	1.36E-08	>0.05	>0.05	>0.05	>0.05
PID SMAD2 3NUCLEAR PATHWAY	0.000971656	0.00020531	>0.05	>0.05	>0.05	>0.05
PID SYNDECAN 2 PATHWAY	0.040868741	>0.05	>0.05	>0.05	2.03E-05	>0.05
PID TCPTP PATHWAY	0.00372682	0.010234168	>0.05	>0.05	>0.05	>0.05
PID TCR PATHWAY	>0.05	>0.05	2.45E-07	0.042224335	>0.05	>0.05
PID THROMBIN PAR1 PATHWAY	0.00019919	0.025212606	>0.05	>0.05	>0.05	>0.05
PID TOLL ENDOGENOUS PATHWAY	>0.05	>0.05	0.001645601	>0.05	>0.05	0.004264338
PID TRAIL PATHWAY	>0.05	>0.05	0.013641055	0.029168171	>0.05	>0.05
PID TXA2PATHWAY	>0.05	>0.05	1.26E-08	9.23E-08	>0.05	>0.05
REACTOME ABACAVIR TRANSPORT AND METABOLISM	0.012372435	0.00075405	>0.05	>0.05	>0.05	>0.05
REACTOME ABCA TRANSPORTERS IN LIPID HOMEOSTASIS	0.000340516	0.000320301	>0.05	>0.05	>0.05	>0.05
REACTOME ADHERENS JUNCTIONS INTERACTIONS	0.005182538	>0.05	>0.05	>0.05	>0.05	1.63E-07
REACTOME AMINO ACID TRANSPORT ACROSS THE PLASMA MEMBRANE	0.036814686	>0.05	0.016913251	>0.05	>0.05	>0.05
REACTOME APOPTOTIC CLEAVAGE OF CELL ADHESION PROTEINS	0.012372435	>0.05	0.012169209	>0.05	>0.05	>0.05

REACTOME APOPTOTIC EXECUTION PHASE	3.96E-05	>0.05	9.07E-08	>0.05	>0.05	>0.05
REACTOME AQUAPORIN MEDIATED TRANSPORT	>0.05	>0.05	>0.05	>0.05	5.01E-05	0.003164299
REACTOME CGMP EFFECTS	>0.05	0.016651362	>0.05	0.001619816	>0.05	>0.05
REACTOME CLASS A1 RHODOPSIN LIKE RECEPTORS	>0.05	0.000	>0.05	8.00E-10	>0.05	>0.05
REACTOME COSTIMULATION BY THE CD28 FAMILY	>0.05	0.03010174	0.000305961	>0.05	>0.05	>0.05
REACTOME CTNNB1 PHOSPHORYLATION CASCADE	0.000340516	>0.05	0.027417779	>0.05	>0.05	>0.05
REACTOME CYTOCHROME P450 ARRANGED BY SUBSTRATE TYPE	>0.05	>0.05	9.63E-08	0.036301094	>0.05	>0.05
REACTOME CYTOKINE SIGNALING IN IMMUNE SYSTEM	>0.05	2.52E-08	5.36E-12	>0.05	>0.05	>0.05
REACTOME FRS2 MEDIATED CASCADE	9.75E-06	8.61E-10	>0.05	>0.05	>0.05	>0.05
REACTOME GAP JUNCTION ASSEMBLY	>0.05	>0.05	0.000100371	0.000128387	>0.05	>0.05
REACTOME GAP JUNCTION TRAFFICKING	>0.05	>0.05	2.31E-05	0.001189241	>0.05	>0.05
REACTOME GLUCONEOGENESIS	4.71E-07	0.000109375	>0.05	>0.05	>0.05	>0.05
REACTOME GLUCOSE METABOLISM	0.000	0.000	>0.05	>0.05	>0.05	>0.05
REACTOME GLYCOGEN BREAKDOWN GLYCOGENOLYSIS	4.79E-05	0.000114419	>0.05	>0.05	>0.05	>0.05

REACTOME GROWTH HORMONE RECEPTOR SIGNALING	0.001580712	1.91E-11	>0.05	>0.05	>0.05	>0.05
REACTOME G ALPHA Z SIGNALLING EVENTS	>0.05	0.018289308	0.005211518	>0.05	>0.05	>0.05
REACTOME HEPARAN SULFATE HEPARIN HS GAG METABOLISM	0.004747732	>0.05	>0.05	>0.05	0.017534065	>0.05
REACTOME HORMONE SENSITIVE LIPASE HSL MEDIATED TRIACYLGLYCEROL HYDROLYSIS	1.87E-07	1.15E-10	>0.05	>0.05	>0.05	>0.05
REACTOME HS GAG BIOSYNTHESIS	0.017692442	>0.05	0.001262871	>0.05	>0.05	>0.05
REACTOME IL 2 SIGNALING	>0.05	8.07E-08	3.01E-05	>0.05	>0.05	>0.05
REACTOME IL 7 SIGNALING	>0.05	0.004775213	0.031991684	>0.05	>0.05	>0.05
REACTOME INHIBITION OF INSULIN SECRETION BY ADRENALINE NORADRENALINE	0.047731345	0.006295117	>0.05	>0.05	>0.05	>0.05
REACTOME INHIBITION OF REPLICATION INITIATION OF DAMAGED DNA BY RB1 E2F1	>0.05	0.020022942	0.04214775	>0.05	>0.05	>0.05
REACTOME INSULIN RECEPTOR SIGNALLING CASCADE	4.41E-15	0.000	>0.05	>0.05	>0.05	>0.05
REACTOME ION TRANSPORT BY P TYPE ATPASES	>0.05	>0.05	>0.05	>0.05	3.69E-05	8.25E-11
REACTOME JNK C JUN KINASES PHOSPHORYLATION AND ACTIVATION MEDIATED BY ACTIVATED HUMAN TAK1	0.019890934	0.011181658	>0.05	>0.05	>0.05	>0.05
REACTOME LIPID DIGESTION MOBILIZATION AND TRANSPORT	1.77E-06	0.000175218	>0.05	>0.05	>0.05	>0.05
REACTOME METAL ION SLC TRANSPORTERS	>0.05	>0.05	>0.05	0.020896515	1.57E-10	>0.05

REACTOME NEGATIVE REGULATION OF FGFR SIGNALING	0.001891942	1.92E-05	>0.05	>0.05	>0.05	>0.05
REACTOME NEPHRIN INTERACTIONS	0.045200672	0.016651362	>0.05	>0.05	>0.05	>0.05
REACTOME NRAGE SIGNALS DEATH THROUGH JNK	3.67E-05	>0.05	0.005754293	>0.05	>0.05	>0.05
REACTOME NUCLEOTIDE LIKE PURINERGIC RECEPTORS	>0.05	0.001209388	>0.05	0.015908376	>0.05	>0.05
REACTOME PASSIVE TRANSPORT BY AQUAPORINS	>0.05	>0.05	>0.05	>0.05	0.027545643	0.001838726
REACTOME PI3K AKT ACTIVATION	>0.05	1.68E-08	>0.05	0.015012914	>0.05	>0.05
REACTOME PI3K CASCADE	5.58E-11	0.000	>0.05	>0.05	>0.05	>0.05
REACTOME PKB MEDIATED EVENTS	0.045307177	0.02326948	>0.05	>0.05	>0.05	>0.05
REACTOME PLC BETA MEDIATED EVENTS	>0.05	0.010754795	>0.05	>0.05	0.002098056	>0.05
REACTOME PRE NOTCH EXPRESSION AND PROCESSING	>0.05	>0.05	0.00336499	>0.05	4.53E-08	>0.05
REACTOME PRE NOTCH TRANSCRIPTION AND TRANSLATION	>0.05	>0.05	2.10E-05	>0.05	0.000371481	>0.05
REACTOME RAP1 SIGNALLING	0.009787131	0.045342531	>0.05	>0.05	>0.05	>0.05
REACTOME RECRUITMENT OF MITOTIC CENTROSOME PROTEINS AND COMPLEXES	>0.05	0.000333893	>0.05	>0.05	5.66E-07	>0.05
REACTOME REGULATION OF WATER BALANCE BY RENAL AQUAPORINS	>0.05	>0.05	>0.05	>0.05	0.000419634	0.017481045

REACTOME SEMAPHORIN INTERACTIONS	>0.05	>0.05	4.04E-05	0.000930386	>0.05	>0.05
REACTOME SIGNALING BY BMP	>0.05	>0.05	2.74E-05	0.001679086	>0.05	>0.05
REACTOME SIGNALING BY FGFR1 FUSION MUTANTS	>0.05	0.000320301	0.00081916	>0.05	>0.05	>0.05
REACTOME SIGNALING BY FGFR1 MUTANTS	0.013823822	2.44E-05	>0.05	>0.05	>0.05	>0.05
REACTOME SIGNALING BY INSULIN RECEPTOR	1.23E-06	0.000	>0.05	>0.05	>0.05	>0.05
REACTOME SIGNALING BY NOTCH	>0.05	>0.05	6.78E-12	>0.05	9.93E-09	>0.05
REACTOME SIGNALLING BY NGF	0.022751964	1.89E-08	>0.05	>0.05	>0.05	>0.05
REACTOME SIGNAL ATTENUATION	0.034683762	0.00762174	>0.05	>0.05	>0.05	>0.05
REACTOME SULFUR AMINO ACID METABOLISM	0.002778705	0.00489792	>0.05	>0.05	>0.05	>0.05
REACTOME SYNTHESIS OF PIPS AT THE PLASMA MEMBRANE	>0.05	>0.05	0.000402541	6.52E-05	>0.05	>0.05
REACTOME SYNTHESIS OF SUBSTRATES IN N GLYCAN BIOSYTHESIS	0.022123585	0.019471996	>0.05	>0.05	>0.05	>0.05
REACTOME TERMINATION OF O GLYCAN BIOSYNTHESIS	>0.05	>0.05	0.018396978	1.77E-14	>0.05	>0.05
REACTOME TRANSMEMBRANE TRANSPORT OF SMALL MOLECULES	>0.05	>0.05	>0.05	>0.05	2.06E-08	0.000
REACTOME UNWINDING OF DNA	0.00777392	9.64E-05	>0.05	>0.05	>0.05	>0.05

REACTOME YAP1 AND WWTR1 TAZ STIMULATED GENE EXPRESSION	0.013746839	>0.05	>0.05	>0.05	0.010164196	>0.05
SA PROGRAMMED CELL DEATH	0.002030526	0.000819292	>0.05	>0.05	>0.05	>0.05
SA REG CASCADE OF CYCLIN EXPR	>0.05	>0.05	0.034832973	0.008793765	>0.05	>0.05
SIG CD40PATHWAYMAP	1.20E-08	0.000175218	>0.05	>0.05	>0.05	>0.05
SIG IL4RECEPTOR IN B LYPHOCYTES	0.005182538	0.004075848	>0.05	>0.05	>0.05	>0.05
SIG INSULIN RECEPTOR PATHWAY IN CARDIAC MYOCYTES	0.000582749	0.002702203	>0.05	>0.05	>0.05	>0.05
SIG PIP3 SIGNALING IN B LYMPHOCYTES	>0.05	>0.05	0.000	1.10E-12	>0.05	>0.05
SIG PIP3 SIGNALING IN CARDIAC MYOCTES	0.006826973	0.005669563	>0.05	>0.05	>0.05	>0.05
ST GA12 PATHWAY	>0.05	>0.05	1.34E-05	0.021377897	>0.05	>0.05
ST STAT3 PATHWAY	>0.05	>0.05	0.031991684	0.001077033	>0.05	>0.05
ST T CELL SIGNAL TRANSDUCTION	>0.05	>0.05	1.35E-09	0.009017611	>0.05	>0.05
BIOCARTA ACTINY PATHWAY	0.002856887	>0.05	>0.05	>0.05	>0.05	>0.05
BIOCARTA AMI PATHWAY	>0.05	>0.05	1.34E-10	>0.05	>0.05	>0.05
BIOCARTA BCR PATHWAY	>0.05	>0.05	0.00835466	>0.05	>0.05	>0.05

BIOCARTA CARM1 PATHWAY	>0.05	>0.05	>0.05	>0.05	0.034393839	>0.05
BIOCARTA ECM PATHWAY	2.71E-05	>0.05	>0.05	>0.05	>0.05	>0.05
BIOCARTA EGF PATHWAY	>0.05	>0.05	0.013514476	>0.05	>0.05	>0.05
BIOCARTA ERK5 PATHWAY	>0.05	0.037464138	>0.05	>0.05	>0.05	>0.05
BIOCARTA EXTRINSIC PATHWAY	>0.05	>0.05	8.50E-06	>0.05	>0.05	>0.05
BIOCARTA FCER1 PATHWAY	>0.05	>0.05	6.41E-07	>0.05	>0.05	>0.05
BIOCARTA GCR PATHWAY	>0.05	0.0043616	>0.05	>0.05	>0.05	>0.05
BIOCARTA GLEEVEC PATHWAY	0.039807053	>0.05	>0.05	>0.05	>0.05	>0.05
BIOCARTA GSK3 PATHWAY	>0.05	5.06E-05	>0.05	>0.05	>0.05	>0.05
BIOCARTA HER2 PATHWAY	0.047739256	>0.05	>0.05	>0.05	>0.05	>0.05
BIOCARTA IGF1MTOR PATHWAY	6.81E-07	>0.05	>0.05	>0.05	>0.05	>0.05
BIOCARTA IGF1R PATHWAY	>0.05	0.000508107	>0.05	>0.05	>0.05	>0.05
BIOCARTA IL17 PATHWAY	>0.05	>0.05	>0.05	0.003732902	>0.05	>0.05
BIOCARTA IL6 PATHWAY	>0.05	>0.05	0.003406225	>0.05	>0.05	>0.05

BIOCARTA INTEGRIN PATHWAY	0.001500184	>0.05	>0.05	>0.05	>0.05	>0.05
BIOCARTA INTRINSIC PATHWAY	>0.05	>0.05	1.06E-06	>0.05	>0.05	>0.05
BIOCARTA LECTIN PATHWAY	>0.05	>0.05	>0.05	>0.05	0.046452223	>0.05
BIOCARTA MAL PATHWAY	>0.05	>0.05	>0.05	>0.05	0.033630113	>0.05
BIOCARTA MAPK PATHWAY	>0.05	>0.05	6.46E-06	>0.05	>0.05	>0.05
BIOCARTA MET PATHWAY	0.002895532	>0.05	>0.05	>0.05	>0.05	>0.05
BIOCARTA NFAT PATHWAY	>0.05	>0.05	>0.05	>0.05	>0.05	6.69E-05
BIOCARTA NFKB PATHWAY	>0.05	>0.05	>0.05	0.00877738	>0.05	>0.05
BIOCARTA NO1 PATHWAY	>0.05	0.018461921	>0.05	>0.05	>0.05	>0.05
BIOCARTA NTHI PATHWAY	>0.05	>0.05	>0.05	>0.05	0.001792848	>0.05
BIOCARTA PLCE PATHWAY	>0.05	>0.05	>0.05	>0.05	0.002513318	>0.05
BIOCARTA SARS PATHWAY	>0.05	>0.05	>0.05	>0.05	>0.05	0.008672108
BIOCARTA SHH PATHWAY	>0.05	>0.05	>0.05	>0.05	0.012260961	>0.05
BIOCARTA SPRY PATHWAY	>0.05	0.033386855	>0.05	>0.05	>0.05	>0.05

BIOCARTA TALL1 PATHWAY	>0.05	>0.05	>0.05	>0.05	3.01E-07	>0.05
BIOCARTA TCYTOTOXIC PATHWAY	>0.05	>0.05	0.022150343	>0.05	>0.05	>0.05
BIOCARTA THELPER PATHWAY	>0.05	>0.05	0.04214775	>0.05	>0.05	>0.05
KEGG AMINO SUGAR AND NUCLEOTIDE SUGAR METABOLISM	>0.05	>0.05	>0.05	>0.05	1.69E-07	>0.05
KEGG BASE EXCISION REPAIR	>0.05	0.004939378	>0.05	>0.05	>0.05	>0.05
KEGG CALCIUM SIGNALING PATHWAY	>0.05	>0.05	>0.05	>0.05	>0.05	8.26E-13
KEGG DRUG METABOLISM OTHER ENZYMES	>0.05	>0.05	>0.05	>0.05	5.23E-05	>0.05
KEGG ENDOCYTOSIS	>0.05	>0.05	6.60E-07	>0.05	>0.05	>0.05
KEGG GLIOMA	>0.05	>0.05	7.59E-08	>0.05	>0.05	>0.05
KEGG GLYCOSAMINOGLYCAN BIOSYNTHESIS KERATAN SULFA	>0.05	>0.05	>0.05	6.68E-06	>0.05	>0.05
KEGG GLYCOSPHINGOLIPID BIOSYNTHESIS GLOBO SERIES	>0.05	>0.05	>0.05	0.015908376	>0.05	>0.05
KEGG GLYCOSPHINGOLIPID BIOSYNTHESIS LACTO AND NEOLACTO SERIES	>0.05	>0.05	>0.05	1.12E-10	>0.05	>0.05
KEGG INOSITOL PHOSPHATE METABOLISM	>0.05	>0.05	>0.05	>0.05	>0.05	0.004524731
KEGG LONG TERM DEPRESSION	>0.05	>0.05	0.03603677	>0.05	>0.05	>0.05

KEGG MAPK SIGNALING PATHWAY	>0.05	>0.05	0.000424507	>0.05	>0.05	>0.05
KEGG NUCLEOTIDE EXCISION REPAIR	>0.05	>0.05	0.001366774	>0.05	>0.05	>0.05
KEGG OXIDATIVE PHOSPHORYLATION	>0.05	>0.05	>0.05	>0.05	>0.05	0.000
KEGG PENTOSE PHOSPHATE PATHWAY	>0.05	>0.05	>0.05	>0.05	0.001878993	>0.05
KEGG PHENYLALANINE METABOLISM	0.040563481	>0.05	>0.05	>0.05	>0.05	>0.05
KEGG PORPHYRIN AND CHLOROPHYLL METABOLISM	>0.05	>0.05	>0.05	>0.05	0.002393906	>0.05
KEGG REGULATION OF ACTIN CYTOSKELETON	4.41E-15	>0.05	>0.05	>0.05	>0.05	>0.05
KEGG RIG I LIKE RECEPTOR SIGNALING PATHWAY	0.001088935	>0.05	>0.05	>0.05	>0.05	>0.05
KEGG T CELL RECEPTOR SIGNALING PATHWAY	>0.05	>0.05	9.63E-08	>0.05	>0.05	>0.05
PID A6B1 A6B4 INTEGRIN PATHWAY	0.000141507	>0.05	>0.05	>0.05	>0.05	>0.05
PID ALPHA SYNUCLEIN PATHWAY	>0.05	>0.05	0.001794062	>0.05	>0.05	>0.05
PID AR PATHWAY	>0.05	>0.05	6.88E-09	>0.05	>0.05	>0.05
PID CDC42 REG PATHWAY	9.34E-07	>0.05	>0.05	>0.05	>0.05	>0.05
PID ECADHERIN KERATINOCYTE PATHWAY	1.77E-06	>0.05	>0.05	>0.05	>0.05	>0.05

PID ECADHERIN NASCENT AJ PATHWAY	7.71E-06	>0.05	>0.05	>0.05	>0.05	>0.05
PID EPHA2 FWD PATHWAY	8.32E-05	>0.05	>0.05	>0.05	>0.05	>0.05
PID EPHRINB REV PATHWAY	>0.05	>0.05	0.000	>0.05	>0.05	>0.05
PID EPO PATHWAY	>0.05	>0.05	1.13E-05	>0.05	>0.05	>0.05
PID ERBB2 ERBB3 PATHWAY	0.000131992	>0.05	>0.05	>0.05	>0.05	>0.05
PID ERBB NETWORK PATHWAY	>0.05	>0.05	0.021694406	>0.05	>0.05	>0.05
PID GMCSF PATHWAY	>0.05	>0.05	3.73E-05	>0.05	>0.05	>0.05
PID IL1 PATHWAY	>0.05	>0.05	0.008818927	>0.05	>0.05	>0.05
PID IL8 CXCR1 PATHWAY	>0.05	>0.05	0.002447702	>0.05	>0.05	>0.05
PID LIS1 PATHWAY	0.03160415	>0.05	>0.05	>0.05	>0.05	>0.05
PID NECTIN PATHWAY	4.74E-07	>0.05	>0.05	>0.05	>0.05	>0.05
PID P38 ALPHA BETA PATHWAY	>0.05	>0.05	1.74E-06	>0.05	>0.05	>0.05
PID PDGFRB PATHWAY	4.67E-10	>0.05	>0.05	>0.05	>0.05	>0.05
PID PI3KCI PATHWAY	>0.05	>0.05	>0.05	0.000600335	>0.05	>0.05

PID PS1 PATHWAY	>0.05	>0.05	2.31E-06	>0.05	>0.05	>0.05
PID REELIN PATHWAY	0.002573733	>0.05	>0.05	>0.05	>0.05	>0.05
PID RETINOIC ACID PATHWAY	0.002006705	>0.05	>0.05	>0.05	>0.05	>0.05
PID RET PATHWAY	>0.05	0.015289937	>0.05	>0.05	>0.05	>0.05
PID S1P S1P3 PATHWAY	>0.05	0.005157213	>0.05	>0.05	>0.05	>0.05
PID S1P S1P4 PATHWAY	>0.05	0.019471996	>0.05	>0.05	>0.05	>0.05
PID TGFBR PATHWAY	>0.05	>0.05	6.63E-12	>0.05	>0.05	>0.05
PID VEGFR1 PATHWAY	>0.05	0.044605211	>0.05	>0.05	>0.05	>0.05
REACTOME ACTIVATED AMPK STIMULATES FATTY ACID OXIDATION IN MUSCLE	>0.05	0.037464138	>0.05	>0.05	>0.05	>0.05
REACTOME ACTIVATED NOTCH1 TRANSMITS SIGNAL TO THE NUCLEUS	>0.05	>0.05	8.02E-07	>0.05	>0.05	>0.05
REACTOME ACTIVATION OF RAC	>0.05	>0.05	>0.05	0.00165468	>0.05	>0.05
REACTOME ADVANCED GLYCOSYLATION ENDPRODUCT RECEPTOR SIGNALING	>0.05	>0.05	>0.05	0.005048181	>0.05	>0.05
REACTOME AMINE COMPOUND SLC TRANSPORTERS	>0.05	>0.05	7.43E-06	>0.05	>0.05	>0.05
REACTOME AMINO ACID SYNTHESIS AND INTERCONVERSION TRANSAMINATION	>0.05	>0.05	0.01674994	>0.05	>0.05	>0.05

REACTOME ANTIGEN ACTIVATES B CELL RECEPTOR LEADING TO GENERATION OF SECOND MESSENGERS	>0.05	>0.05	0.002375664	>0.05	>0.05	>0.05
REACTOME APC C CDH1 MEDIATED DEGRADATION OF CDC20 AND OTHER APC C CDH1 TARGETED PROTEINS IN LATE MITOSIS EARLY G1	>0.05	0.037218646	>0.05	>0.05	>0.05	>0.05
REACTOME AXON GUIDANCE	>0.05	>0.05	>0.05	>0.05	>0.05	2.40E-08
REACTOME A TETRASACCHARIDE LINKER SEQUENCE IS REQUIRED FOR GAG SYNTHESIS	4.13E-13	>0.05	>0.05	>0.05	>0.05	>0.05
REACTOME BMAL1 CLOCK NPAS2 ACTIVATES CIRCADIAN EXPRESSION	>0.05	>0.05	>0.05	>0.05	0.002934296	>0.05
REACTOME CA DEPENDENT EVENTS	>0.05	>0.05	>0.05	>0.05	0.01204517	>0.05
REACTOME CDC6 ASSOCIATION WITH THE ORC ORIGIN COMPLEX	>0.05	0.037206948	>0.05	>0.05	>0.05	>0.05
REACTOME CHOLESTEROL BIOSYNTHESIS	>0.05	2.25E-06	>0.05	>0.05	>0.05	>0.05
REACTOME CHONDROITIN SULFATE BIOSYNTHESIS	>0.05	>0.05	>0.05	>0.05	>0.05	0.008672108
REACTOME CHROMOSOME MAINTE>0.05CE	>0.05	0.000	>0.05	>0.05	>0.05	>0.05
REACTOME CHYLOMICRON MEDIATED LIPID TRANSPORT	0.002030526	>0.05	>0.05	>0.05	>0.05	>0.05
REACTOME CLASS B 2 SECRETIN FAMILY RECEPTORS	>0.05	>0.05	1.27E-05	>0.05	>0.05	>0.05
REACTOME CLEAVAGE OF GROWING TRANSCRIPT IN THE TERMINATION REGION	>0.05	>0.05	>0.05	>0.05	0.0026553	>0.05

REACTOME COMMON PATHWAY	>0.05	>0.05	0.001277672	>0.05	>0.05	>0.05
REACTOME CRMPS IN SEMA3A SIGNALING	>0.05	>0.05	0.005308416	>0.05	>0.05	>0.05
REACTOME DARPP 32 EVENTS	>0.05	0.024242725	>0.05	>0.05	>0.05	>0.05
REACTOME DEPOSITION OF NEW CENPA CONTAINING NUCLEOSOMES AT THE CENTROMERE	>0.05	0.001259265	>0.05	>0.05	>0.05	>0.05
REACTOME DOWNSTREAM SIGNALING OF ACTIVATED FGFR	>0.05	1.49E-07	>0.05	>0.05	>0.05	>0.05
REACTOME EGFR DOWNREGULATION	>0.05	0.038435674	>0.05	>0.05	>0.05	>0.05
REACTOME ENERGY DEPENDENT REGULATION OF MTOR BY LKB1 AMPK	>0.05	>0.05	0.014750909	>0.05	>0.05	>0.05
REACTOME EXTENSION OF TELOMERES	>0.05	>0.05	0.004073915	>0.05	>0.05	>0.05
REACTOME FACILITATIVE NA INDEPENDENT GLUCOSE TRANSPORTERS	>0.05	>0.05	>0.05	>0.05	0.027545643	>0.05
REACTOME FGFR4 LIGAND BINDING AND ACTIVATION	>0.05	>0.05	0.022150343	>0.05	>0.05	>0.05
REACTOME FORMATION OF FIBRIN CLOT CLOTTING CASCADE	>0.05	>0.05	0.000665401	>0.05	>0.05	>0.05
REACTOME GENERATION OF SECOND MESSENGER MOLECULES	>0.05	>0.05	0.039274547	>0.05	>0.05	>0.05
REACTOME GLOBAL GENOMIC NER GG NER	>0.05	>0.05	1.63E-08	>0.05	>0.05	>0.05
REACTOME GLUCOSE TRANSPORT	>0.05	>0.05	>0.05	>0.05	3.21E-11	>0.05

REACTOME GLYCOLYSIS	0.044607191	>0.05	>0.05	>0.05	>0.05	>0.05
REACTOME GLYCOPROTEIN HORMONES	>0.05	>0.05	>0.05	>0.05	>0.05	1.07E-05
REACTOME GOLGI ASSOCIATED VESICLE BIOGENESIS	6.18E-07	>0.05	>0.05	>0.05	>0.05	>0.05
REACTOME GRB2 SOS PROVIDES LINKAGE TO MAPK SIGNALING FOR INTERGRINS	>0.05	>0.05	0.000905552	>0.05	>0.05	>0.05
REACTOME G ALPHA1213 SIGNALLING EVENTS	0.018172173	>0.05	>0.05	>0.05	>0.05	>0.05
REACTOME HS GAG DEGRADATION	4.62E-06	>0.05	>0.05	>0.05	>0.05	>0.05
REACTOME IMMUNE SYSTEM	>0.05	>0.05	0.000	>0.05	>0.05	>0.05
REACTOME INSULIN SYNTHESIS AND PROCESSING	>0.05	>0.05	0.003308991	>0.05	>0.05	>0.05
REACTOME INTEGRATION OF ENERGY METABOLISM	>0.05	0.012212152	>0.05	>0.05	>0.05	>0.05
REACTOME INTEGRIN ALPHAIIB BETA3 SIGNALING	>0.05	>0.05	0.002787672	>0.05	>0.05	>0.05
REACTOME INTERACTIONS OF VPR WITH HOST CELLULAR PROTEINS	>0.05	>0.05	>0.05	>0.05	3.75E-05	>0.05
REACTOME INTERACTION BETWEEN L1 AND ANKYRINS	>0.05	>0.05	0.026990725	>0.05	>0.05	>0.05
REACTOME INTRINSIC PATHWAY FOR APOPTOSIS	>0.05	>0.05	>0.05	>0.05	0.040670878	>0.05
REACTOME ION CHANNEL TRANSPORT	>0.05	>0.05	>0.05	>0.05	>0.05	5.00E-05

REACTOME IRAK1 RECRUITS IKK COMPLEX	>0.05	0.023818998	>0.05	>0.05	>0.05	>0.05
REACTOME KERATAN SULFATE DEGRADATION	>0.05	0.004775213	>0.05	>0.05	>0.05	>0.05
REACTOME L1CAM INTERACTIONS	0.001088935	>0.05	>0.05	>0.05	>0.05	>0.05
REACTOME LAGGING STRAND SYNTHESIS	>0.05	>0.05	0.016797108	>0.05	>0.05	>0.05
REACTOME LIPOPROTEIN METABOLISM	0.013823822	>0.05	>0.05	>0.05	>0.05	>0.05
REACTOME LOSS OF NLP FROM MITOTIC CENTROSOMES	>0.05	>0.05	>0.05	>0.05	0.033630113	>0.05
REACTOME MEIOSIS	>0.05	0.013136865	>0.05	>0.05	>0.05	>0.05
REACTOME MEIOTIC SYNAPSIS	>0.05	0.000110548	>0.05	>0.05	>0.05	>0.05
REACTOME MEMBRANE TRAFFICKING	0.010680085	>0.05	>0.05	>0.05	>0.05	>0.05
REACTOME METABOLISM OF AMINO ACIDS AND DERIVATIVES	>0.05	>0.05	>0.05	>0.05	>0.05	0.000
REACTOME METABOLISM OF NON CODING RNA	>0.05	>0.05	>0.05	>0.05	6.24E-07	>0.05
REACTOME METABOLISM OF STEROID HORMONES AND VITAMINS A AND D	>0.05	>0.05	>0.05	>0.05	0.000784398	>0.05
REACTOME MRNA 3 END PROCESSING	>0.05	>0.05	>0.05	>0.05	3.01E-07	>0.05
REACTOME MRNA PROCESSING	>0.05	>0.05	>0.05	>0.05	6.02E-08	>0.05

REACTOME NCAM1 INTERACTIONS	>0.05	>0.05	>0.05	>0.05	>0.05	0.041673395
REACTOME NCAM SIGNALING FOR NEURITE OUT GROWTH	>0.05	>0.05	>0.05	>0.05	>0.05	1.26E-08
REACTOME NEF MEDIATED DOWNREGULATION OF MHC CLASS I COMPLEX CELL SURFACE EXPRESSION	>0.05	>0.05	>0.05	0.034708443	>0.05	>0.05
REACTOME NEGATIVE REGULATORS OF RIG I MDA5 SIGNALING	0.002006705	>0.05	>0.05	>0.05	>0.05	>0.05
REACTOME NEP NS2 INTERACTS WITH THE CELLULAR EXPORT MACHINERY	>0.05	>0.05	>0.05	>0.05	2.99E-08	>0.05
REACTOME NGF SIGNALLING VIA TRKA FROM THE PLASMA MEMBRANE	>0.05	0.000	>0.05	>0.05	>0.05	>0.05
REACTOME NITRIC OXIDE STIMULATES GUANYLATE CYCLASE	>0.05	>0.05	>0.05	0.028041436	>0.05	>0.05
REACTOME NOD1 2 SIGNALING PATHWAY	0.006622823	>0.05	>0.05	>0.05	>0.05	>0.05
REACTOME NOREPINEPHRINE NEUROTRANSMITTER RELEASE CYCLE	>0.05	>0.05	0.027316664	>0.05	>0.05	>0.05
REACTOME NUCLEOTIDE EXCISION REPAIR	>0.05	>0.05	0.018566866	>0.05	>0.05	>0.05
REACTOME OPIOID SIGNALLING	>0.05	0.009466235	>0.05	>0.05	>0.05	>0.05
REACTOME P130CAS LINKAGE TO MAPK SIGNALING FOR INTEGRINS	>0.05	>0.05	0.00356802	>0.05	>0.05	>0.05
REACTOME P2Y RECEPTORS	>0.05	0.037206948	>0.05	>0.05	>0.05	>0.05
REACTOME PACKAGING OF TELOMERE ENDS	>0.05	0.001598609	>0.05	>0.05	>0.05	>0.05

REACTOME PEPTIDE HORMONE BIOSYNTHESIS	>0.05	>0.05	>0.05	>0.05	>0.05	4.48E-07
REACTOME PERK REGULATED GENE EXPRESSION	>0.05	>0.05	>0.05	>0.05	0.024820589	>0.05
REACTOME PEROXISOMAL LIPID METABOLISM	>0.05	>0.05	>0.05	>0.05	0.001739315	>0.05
REACTOME PHASE II CONJUGATION	>0.05	>0.05	>0.05	>0.05	8.16E-06	>0.05
REACTOME PHOSPHOLIPASE C MEDIATED CASCADE	>0.05	0.009071566	>0.05	>0.05	>0.05	>0.05
REACTOME PI3K EVENTS IN ERBB2 SIGNALING	>0.05	>0.05	>0.05	0.000179097	>0.05	>0.05
REACTOME PIP3 ACTIVATES AKT SIGNALING	>0.05	>0.05	>0.05	0.001184885	>0.05	>0.05
REACTOME PKA MEDIATED PHOSPHORYLATION OF CREB	>0.05	>0.05	>0.05	>0.05	0.026495998	>0.05
REACTOME POST TRANSLATIONAL PROTEIN MODIFICATION	>0.05	>0.05	>0.05	1.14E-10	>0.05	>0.05
REACTOME PROCESSING OF CAPPED INTRON CONTAINING PRE MRNA	>0.05	>0.05	>0.05	>0.05	4.47E-06	>0.05
REACTOME PROLACTIN RECEPTOR SIGNALING	>0.05	0.041484073	>0.05	>0.05	>0.05	>0.05
REACTOME PURINE METABOLISM	>0.05	>0.05	>0.05	>0.05	1.34E-07	>0.05
REACTOME PURINE RIBONUCLEOSIDE MONOPHOSPHATE BIOSYNTHESIS	>0.05	>0.05	>0.05	>0.05	0.010257014	>0.05
REACTOME PURINE SALVAGE	>0.05	>0.05	>0.05	>0.05	0.014901823	>0.05

REACTOME PYRIMIDINE METABOLISM	>0.05	0.001399326	>0.05	>0.05	>0.05	>0.05
REACTOME PYRUVATE METABOLISM	0.022763171	>0.05	>0.05	>0.05	>0.05	>0.05
REACTOME PYRUVATE METABOLISM AND CITRIC ACID TCA CYCLE	8.48E-07	>0.05	>0.05	>0.05	>0.05	>0.05
REACTOME RAF MAP KINASE CASCADE	>0.05	>0.05	>0.05	>0.05	0.016331761	>0.05
REACTOME REGULATION OF AMPK ACTIVITY VIA LKB1	>0.05	>0.05	0.01894335	>0.05	>0.05	>0.05
REACTOME REGULATION OF COMPLEMENT CASCADE	>0.05	>0.05	0.04214775	>0.05	>0.05	>0.05
REACTOME REGULATION OF GLUCOKINASE BY GLUCOKINASE REGULATORY PROTEIN	>0.05	>0.05	>0.05	>0.05	1.26E-13	>0.05
REACTOME REGULATION OF HYPOXIA INDUCIBLE FACTOR HIF BY OXYGEN	>0.05	>0.05	>0.05	>0.05	>0.05	4.31E-06
REACTOME REGULATION OF INSULIN LIKE GROWTH FACTOR IGF ACTIVITY BY INSULIN LIKE GROWTH FACTOR BINDING PROTEINS IGFBPS	0.00181339	>0.05	>0.05	>0.05	>0.05	>0.05
REACTOME REGULATION OF PYRUVATE DEHYDROGENASE PDH COMPLEX	0.000202361	>0.05	>0.05	>0.05	>0.05	>0.05
REACTOME REPAIR SYNTHESIS FOR GAP FILLING BY DNA POL IN TC NER	>0.05	>0.05	0.005308416	>0.05	>0.05	>0.05
REACTOME RNA POL I RNA POL III AND MITOCHONDRIAL TRANSCRIPTION	>0.05	1.28E-08	>0.05	>0.05	>0.05	>0.05
REACTOME RNA POL I TRANSCRIPTION	>0.05	1.27E-06	>0.05	>0.05	>0.05	>0.05
REACTOME RNA POL I TRANSCRIPTION TERMINATION	>0.05	>0.05	0.026990725	>0.05	>0.05	>0.05

REACTOME SEMA4D IN SEMAPHORIN SIGNALING	0.047603346	>0.05	>0.05	>0.05	>0.05	>0.05
REACTOME SIGNALING BY EGFR IN CANCER	>0.05	0.019471996	>0.05	>0.05	>0.05	>0.05
REACTOME SIGNALING BY ERBB4	>0.05	0.043008617	>0.05	>0.05	>0.05	>0.05
REACTOME SIGNALING BY FGFR	>0.05	8.65E-07	>0.05	>0.05	>0.05	>0.05
REACTOME SIGNALING BY FGFR3 MUTANTS	0.020988144	>0.05	>0.05	>0.05	>0.05	>0.05
REACTOME SIGNALING BY FGFR IN DISEASE	>0.05	1.91E-10	>0.05	>0.05	>0.05	>0.05
REACTOME SIGNALING BY FGFR MUTANTS	>0.05	0.007766328	>0.05	>0.05	>0.05	>0.05
REACTOME SIGNALING BY NODAL	>0.05	0.045342531	>0.05	>0.05	>0.05	>0.05
REACTOME SIGNALING BY NOTCH1	>0.05	>0.05	0.000132336	>0.05	>0.05	>0.05
REACTOME SIGNALING BY NOTCH3	>0.05	>0.05	0.04214775	>0.05	>0.05	>0.05
REACTOME SIGNAL AMPLIFICATION	>0.05	0.037218646	>0.05	>0.05	>0.05	>0.05
REACTOME SLC MEDIATED TRANSMEMBRANE TRANSPORT	>0.05	>0.05	>0.05	>0.05	0.000	>0.05
REACTOME SMAD2 SMAD3 SMAD4 HETEROTRIMER REGULATES TRANSCRIPTION	0.032098056	>0.05	>0.05	>0.05	>0.05	>0.05
REACTOME SOS MEDIATED SIGNALLING	>0.05	1.12E-05	>0.05	>0.05	>0.05	>0.05

REACTOME SPHINGOLIPID METABOLISM	>0.05	>0.05	>0.05	>0.05	0.010752282	>0.05
REACTOME SYNTHESIS OF GLYCOSYLPHOSPHATIDYLINOSITOL GPI	>0.05	>0.05	0.005064547	>0.05	>0.05	>0.05
REACTOME SYNTHESIS SECRETION AND DEACYLATION OF GHRELIN	>0.05	0.008291526	>0.05	>0.05	>0.05	>0.05
REACTOME S PHASE	>0.05	0.047017927	>0.05	>0.05	>0.05	>0.05
REACTOME TAK1 ACTIVATES NFKB BY PHOSPHORYLATION ANI	>0.05	>0.05	>0.05	0.031116765	>0.05	>0.05
REACTOME TCR SIGNALING	>0.05	>0.05	0.000398825	>0.05	>0.05	>0.05
REACTOME TELOMERE MAINTE>0.05CE	>0.05	9.79E-11	>0.05	>0.05	>0.05	>0.05
REACTOME THE ROLE OF NEF IN HIV1 REPLICATION AND DISEASE PATHOGENESIS	>0.05	>0.05	>0.05	0.021490337	>0.05	>0.05
REACTOME TRANSPORT OF MATURE MRNA DERIVED FROM AN INTRONLESS TRANSCRIPT	>0.05	>0.05	>0.05	>0.05	4.86E-13	>0.05
REACTOME TRANSPORT OF MATURE TRANSCRIPT TO CYTOPLASM	>0.05	>0.05	>0.05	>0.05	0.000	>0.05
REACTOME TRANSPORT OF RIBONUCLEOPROTEINS INTO THE HOST NUCLEUS	>0.05	>0.05	>0.05	>0.05	2.99E-08	>0.05
REACTOME TRANS GOLGI NETWORK VESICLE BUDDING	2.36E-09	>0.05	>0.05	>0.05	>0.05	>0.05
REACTOME XENOBIOTICS	>0.05	>0.05	>0.05	0.003732902	>0.05	>0.05
REACTOME ZINC TRANSPORTERS	>0.05	>0.05	>0.05	>0.05	0.005345082	>0.05

SA B CELL RECEPTOR COMPLEXES	0.022199429	>0.05	>0.05	>0.05	>0.05	>0.05
SA CASPASE CASCADE	>0.05	>0.05	>0.05	0.003043475	>0.05	>0.05
SIG BCR SIGNALING PATHWAY	>0.05	>0.05	3.23E-06	>0.05	>0.05	>0.05
ST B CELL ANTIGEN RECEPTOR	>0.05	>0.05	0.000505113	>0.05	>0.05	>0.05
ST DIFFERENTIATION PATHWAY IN PC12 CELLS	>0.05	0.007243334	>0.05	>0.05	>0.05	>0.05
ST ERK1 ERK2 MAPK PATHWAY	>0.05	>0.05	7.61E-06	>0.05	>0.05	>0.05
ST GAQ PATHWAY	>0.05	>0.05	0.007154764	>0.05	>0.05	>0.05
ST G ALPHA I PATHWAY	>0.05	>0.05	7.35E-05	>0.05	>0.05	>0.05
ST WNT BETA CATENIN PATHWAY	>0.05	>0.05	0.001883976	>0.05	>0.05	>0.05

Note: Pathways that are enriched in many datasets are listed at the beginning, whereas the disease-specific pathways are listed at the end. Pathways enriched by one, two, three, four, five and six datasets are colored differently.

MSigDB pathways enriched with DR gene pairs identified from $GBM_{70\text{-}13}$

Pathway Name	p value	q value	k	n	x	m	PMID
KEGG MAPK SIGNALING PATHWAY	0.000	0.000	5756553	32308741	8319	34191	26794430
KEGG ERBB SIGNALING PATHWAY	0.000	0.000	5756553	32308741	927	3655	25313012
KEGG CALCIUM SIGNALING PATHWAY	0.000	0.000	5756553	32308741	4052	15225	26826650
KEGG CELL CYCLE	0.000	0.000	5756553	32308741	1633	7503	26983952
KEGG P53 SIGNALING PATHWAY	0.000	0.000	5756553	32308741	565	2278	26986934
KEGG WNT SIGNALING PATHWAY	0.000	0.000	5756553	32308741	2594	10878	26979081
KEGG VEGF SIGNALING PATHWAY	0.000	0.000	5756553	32308741	613	2485	26893618
KEGG FOCAL ADHESION	0.000	0.000	5756553	32308741	4406	19503	25504636
KEGG ECM RECEPTOR INTERACTION	0.000	0.000	5756553	32308741	830	3486	26502847
KEGG GAP JUNCTION	0.000	0.000	5756553	32308741	893	3828	18286483
KEGG NEUROACTIVE LIGAND RECEPTOR INTERACTION	0.000	0.000	5756553	32308741	9417	35778	
KEGG ENDOCYTOSIS	0.000	0.000	5756553	32308741	3364	16110	
KEGG AXON GUIDANCE	0.000	0.000	5756553	32308741	2136	8256	
KEGG LONG TERM POTENTIATION	0.000	0.000	5756553	32308741	622	2415	
KEGG LONG TERM DEPRESSION	0.000	0.000	5756553	32308741	579	2145	
KEGG AMYOTROPHIC LATERAL SCLEROSIS ALS	0.000	0.000	5756553	32308741	379	1378	
KEGG PATHWAYS IN CANCER	0.000	0.000	5756553	32308741	10613	52326	
KEGG PANCREATIC CANCER	0.000	0.000	5756553	32308741	602	2415	
KEGG ACUTE MYELOID LEUKEMIA	0.000	0.000	5756553	32308741	476	1596	
KEGG HYPERTROPHIC CARDIOMYOPATHY HCM	0.000	0.000	5756553	32308741	994	3403	
KEGG DILATED CARDIOMYOPATHY	0.000	0.000	5756553	32308741	1146	4005	
BIOCARTA KERATINOCYTE PATHWAY	0.000	0.000	5756553	32308741	307	1035	
BIOCARTA MAPK PATHWAY	0.000	0.000	5756553	32308741	963	3655	
BIOCARTA P38MAPK PATHWAY	0.000	0.000	5756553	32308741	234	741	
ST ADRENERGIC	0.000	0.000	5756553	32308741	193	595	
ST INTEGRIN SIGNALING PATHWAY	0.000	0.000	5756553	32308741	878	3321	
PID AURORA B PATHWAY	0.000	0.000	5756553	32308741	228	741	
PID WNT NONCANONICAL PATHWAY	0.000	0.000	5756553	32308741	180	496	

PID E2F PATHWAY	0.000	0.000	5756553	32308741	655	2701
PID TXA2PATHWAY	0.000	0.000	5756553	32308741	419	1596
PID PLK1 PATHWAY	0.000	0.000	5756553	32308741	305	1035
PID TRKR PATHWAY	0.000	0.000	5756553	32308741	485	1830
PID VEGFR1 2 PATHWAY	0.000	0.000	5756553	32308741	581	2346
REACTOME SIGNALING BY RHO GTPASES	0.000	0.000	5756553	32308741	1361	5886
REACTOME SIGNALLING BY NGF	0.000	0.000	5756553	32308741	4836	21945
REACTOME DEVELOPMENTAL BIOLOGY	0.000	0.000	5756553	32308741	15713	72010
REACTOME CELL CELL COMMUNICATION	0.000	0.000	5756553	32308741	1480	6786
REACTOME SIGNALING BY EGFR IN CANCER	0.000	0.000	5756553	32308741	1235	5460
REACTOME NGF SIGNALLING VIA TRKA FROM THE PLASMA MEMBRANE	0.000	0.000	5756553	32308741	1935	8646
REACTOME HS GAG BIOSYNTHESIS	0.000	0.000	5756553	32308741	164	435
REACTOME HEPARAN SULFATE HEPARIN HS GAG METABOLISM	0.000	0.000	5756553	32308741	457	1275
REACTOME GLYCOSAMINOGLYCAN METABOLISM	0.000	0.000	5756553	32308741	1648	5778
REACTOME MHC CLASS II ANTIGEN PRESENTATION	0.000	0.000	5756553	32308741	942	3741
REACTOME TRANSMISSION ACROSS CHEMICAL SYNAPSES	0.000	0.000	5756553	32308741	5038	16471
REACTOME NEURONAL SYSTEM	0.000	0.000	5756553	32308741	10652	36856
REACTOME SIGNALING BY GPCR	0.000	0.000	5756553	32308741	38639	2E+05
REACTOME PEPTIDE LIGAND BINDING RECEPTORS	0.000	0.000	5756553	32308741	3298	14878
REACTOME CLASS A1 RHODOPSIN LIKE RECEPTORS	0.000	0.000	5756553	32308741	8446	39060
REACTOME CELL CYCLE MITOTIC	0.000	0.000	5756553	32308741	9469	46971
REACTOME OPIOID SIGNALLING	0.000	0.000	5756553	32308741	752	2926
REACTOME NEUROTRANSMITTER RECEPTOR BINDING AND DOWNSTREAM TRANSMISSION IN THE POSTSYNAPTIC CELL	0.000	0.000	5756553	32308741	2901	8778
REACTOME TRANSMEMBRANE TRANSPORT OF SMALL MOLECULES	0.000	0.000	5756553	32308741	16120	81003
REACTOME SIGNALING BY PDGF	0.000	0.000	5756553	32308741	1660	6903
REACTOME AXON GUIDANCE	0.000	0.000	5756553	32308741	6955	28680
REACTOME CLASS B 2 SECRETIN FAMILY RECEPTORS	0.000	0.000	5756553	32308741	863	3655
REACTOME GPCR DOWNSTREAM SIGNALING	0.000	0.000	5756553	32308741	26752	1E+05
REACTOME G ALPHA Z SIGNALLING EVENTS	0.000	0.000	5756553	32308741	294	946

REACTOME ACTIVATION OF NMDA RECEPTOR UPON GLUTAMATE BINDING AND POSTSYNAPTIC EVENTS	0.000	0.000	5756553	32308741	222	666
REACTOME POST NMDA RECEPTOR ACTIVATION EVENTS	0.000	0.000	5756553	32308741	185	528
REACTOME GPCR LIGAND BINDING	0.000	0.000	5756553	32308741	15917	71631
REACTOME L1CAM INTERACTIONS	0.000	0.000	5756553	32308741	958	3403
REACTOME GABA B RECEPTOR ACTIVATION	0.000	0.000	5756553	32308741	220	703
REACTOME GABA RECEPTOR ACTIVATION	0.000	0.000	5756553	32308741	506	1326
REACTOME ION CHANNEL TRANSPORT	0.000	0.000	5756553	32308741	481	1431
REACTOME MITOTIC PROMETAPHASE	0.000	0.000	5756553	32308741	847	3570
REACTOME POTASSIUM CHANNELS	0.000	0.000	5756553	32308741	1207	4656
NABA ECM GLYCOPROTEINS	0.000	0.000	5756553	32308741	4312	17766
NABA SECRETED FACTORS	0.000	0.000	5756553	32308741	9952	50721
NABA CORE MATRISOME	0.000	0.000	5756553	32308741	8087	35778
NABA MATRISOME	0.000	0.000	5756553	32308741	90357	5E+05
KEGG MELANOGENESIS	4.44E-16	8.20E-15	5756553	32308741	1106	4950
REACTOME LIGAND GATED ION CHANNEL TRANSPORT	4.44E-16	8.20E-15	5756553	32308741	88	210
REACTOME SIGNALING BY NOTCH	8.88E-16	1.62E-14	5756553	32308741	1103	4950
NABA ECM AFFILIATED	1.11E-15	2.00E-14	5756553	32308741	2233	10731
REACTOME G ALPHA I SIGNALLING EVENTS	1.33E-15	2.36E-14	5756553	32308741	3434	17020
BIOCARTA CREB PATHWAY	1.67E-15	2.91E-14	5756553	32308741	125	351
KEGG GNRH SIGNALING PATHWAY	2.22E-15	3.84E-14	5756553	32308741	1041	4656
NABA MATRISOME ASSOCIATED	4.44E-15	7.57E-14	5756553	32308741	44806	2E+05
KEGG ARRHYTHMOGENIC RIGHT VENTRICULAR CARDIOMYOPATHY ARVC	6.22E-15	1.05E-13	5756553	32308741	641	2701
PID ENDOTHELIN PATHWAY	8.33E-15	1.38E-13	5756553	32308741	484	1953
REACTOME CREB PHOSPHORYLATION THROUGH THE ACTIVATION OF RAS	1.07E-14	1.75E-13	5756553	32308741	123	351
PID NFAT 3PATHWAY	1.52E-14	2.47E-13	5756553	32308741	371	1431
REACTOME CHONDROITIN SULFATE DERMATAN SULFATE METABOLISM	1.69E-14	2.70E-13	5756553	32308741	294	1081
PID THROMBIN PAR1 PATHWAY	3.10E-14	4.90E-13	5756553	32308741	253	903

REACTOME CELL JUNCTION ORGANIZATION	4.63E-14	7.24E-13	5756553	32308741	666	2850
PID FOXM1 PATHWAY	5.64E-14	8.72E-13	5756553	32308741	224	780
REACTOME TRAFFICKING OF AMPA RECEPTORS	6.55E-14	1.00E-12	5756553	32308741	121	351
REACTOME E2F MEDIATED REGULATION OF DNA REPLICATION	1.11E-13	1.68E-12	5756553	32308741	164	528
REACTOME PLC BETA MEDIATED EVENTS	2.46E-13	3.67E-12	5756553	32308741	240	861
BIOCARTA G2 PATHWAY	7.73E-13	1.14E-11	5756553	32308741	99	276
REACTOME SYNTHESIS AND INTERCONVERSION OF NUCLEOTIDE DI AND TRIPHOSPHATES	1.19E-12	1.74E-11	5756553	32308741	60	136
PID TGFBR PATHWAY	1.63E-12	2.36E-11	5756553	32308741	361	1431
ST G ALPHA I PATHWAY	1.67E-12	2.39E-11	5756553	32308741	176	595
REACTOME A TETRASACCHARIDE LINKER SEQUENCE IS REQUIRED FOR GAG SYNTHESIS	2.37E-12	3.31E-11	5756553	32308741	104	300
REACTOME LYSOSOME VESICLE BIOGENESIS	2.35E-12	3.31E-11	5756553	32308741	86	231
BIOCARTA FAS PATHWAY	3.55E-12	4.92E-11	5756553	32308741	137	435
REACTOME ADHERENS JUNCTIONS INTERACTIONS	4.97E-12	6.81E-11	5756553	32308741	116	351
REACTOME HEMOSTASIS	8.73E-12	1.18E-10	5756553	32308741	18092	97020
REACTOME G ALPHA Q SIGNALLING EVENTS	1.01E-11	1.35E-10	5756553	32308741	2872	14365
PID NOTCH PATHWAY	1.43E-11	1.90E-10	5756553	32308741	415	1711
BIOCARTA EGF PATHWAY	1.79E-11	2.36E-10	5756553	32308741	142	465
PID LIS1 PATHWAY	1.89E-11	2.47E-10	5756553	32308741	121	378
PID MET PATHWAY	3.52E-11	4.54E-10	5756553	32308741	708	3160
KEGG GLYCOSAMINOGLYCAN BIOSYNTHESIS HEPARAN SULFATE	4.13E-11	5.28E-10	5756553	32308741	107	325
BIOCARTA AGR PATHWAY	4.36E-11	5.52E-10	5756553	32308741	179	630
REACTOME BOTULINUM NEUROTOXICITY	5.24E-11	6.58E-10	5756553	32308741	57	136
REACTOME G1 S SPECIFIC TRANSCRIPTION	1.74E-10	2.17E-09	5756553	32308741	56	136
BIOCARTA G1 PATHWAY	4.01E-10	4.90E-09	5756553	32308741	117	378
REACTOME NCAM SIGNALING FOR NEURITE OUT GROWTH	3.98E-10	4.90E-09	5756553	32308741	469	2016
BIOCARTA BIOPEPTIDES PATHWAY	5.16E-10	6.24E-09	5756553	32308741	226	861
ST MYOCYTE AD PATHWAY	5.91E-10	7.08E-09	5756553	32308741	110	351
REACTOME SIGNALING BY ROBO RECEPTOR	6.46E-10	7.67E-09	5756553	32308741	123	406

REACTOME GASTRIN CREB SIGNALLING PATHWAY VIA PKC AND MAPK	1.29E-09	1.52E-08	5756553	32308741	3544	18145
PID RAS PATHWAY	1.30E-09	1.52E-08	5756553	32308741	122	406
PID CXCR4 PATHWAY	1.41E-09	1.63E-08	5756553	32308741	1085	5151
ST JNK MAPK PATHWAY	1.67E-09	1.91E-08	5756553	32308741	206	780
PID RHOA PATHWAY	2.05E-09	2.34E-08	5756553	32308741	251	990
REACTOME UNBLOCKING OF NMDA RECEPTOR GLUTAMATE BINDING AND ACTIVATION	2.25E-09	2.54E-08	5756553	32308741	45	105
BIOCARTA AT1R PATHWAY	2.32E-09	2.59E-08	5756553	32308741	142	496
REACTOME DAG AND IP3 SIGNALING	4.08E-09	4.48E-08	5756553	32308741	134	465
REACTOME METABOLISM OF CARBOHYDRATES	4.06E-09	4.48E-08	5756553	32308741	5054	26335
REACTOME POST TRANSLATIONAL PROTEIN MODIFICATION	7.17E-09	7.81E-08	5756553	32308741	3116	15931
REACTOME CREB PHOSPHORYLATION THROUGH THE ACTIVATION OF CAMKII	7.78E-09	8.41E-08	5756553	32308741	44	105
ST P38 MAPK PATHWAY	7.90E-09	8.47E-08	5756553	32308741	178	666
BIOCARTA EIF4 PATHWAY	1.15E-08	1.22E-07	5756553	32308741	88	276
REACTOME SLC MEDIATED TRANSMEMBRANE TRANSPORT	1.25E-08	1.32E-07	5756553	32308741	5256	27495
KEGG NOTCH SIGNALING PATHWAY	1.37E-08	1.43E-07	5756553	32308741	266	1081
KEGG DNA REPLICATION	1.39E-08	1.45E-07	5756553	32308741	169	630
REACTOME KINESINS	1.56E-08	1.60E-07	5756553	32308741	82	253
BIOCARTA CCR5 PATHWAY	1.59E-08	1.63E-07	5756553	32308741	52	136
REACTOME TRANSPORT OF INORGANIC CATIONS ANIONS AND AMINO ACIDS OLIGOPEPTIDES	1.82E-08	1.85E-07	5756553	32308741	868	4095
PID REELIN PATHWAY	1.94E-08	1.95E-07	5756553	32308741	118	406
ST FAS SIGNALING PATHWAY	2.00E-08	1.98E-07	5756553	32308741	457	2016
REACTOME CELL CELL JUNCTION ORGANIZATION	1.99E-08	1.98E-07	5756553	32308741	349	1485
PID S1P S1P4 PATHWAY	2.55E-08	2.52E-07	5756553	32308741	39	91
KEGG GLYCOSPHINGOLIPID BIOSYNTHESIS GANGLIO SERIES	2.59E-08	2.53E-07	5756553	32308741	43	105
BIOCARTA CACAM PATHWAY	3.17E-08	3.08E-07	5756553	32308741	35	78
PID IFNG PATHWAY	3.27E-08	3.15E-07	5756553	32308741	200	780
BIOCARTA TNFR1 PATHWAY	3.69E-08	3.53E-07	5756553	32308741	117	406

PID ERBB1 INTERNALIZATION PATHWAY	4.30E-08	4.09E-07	5756553	32308741	208	820
REACTOME RAS ACTIVATION UOPN CA2 INFUX THROUGH NMDA RECEPTOR	4.56E-08	4.30E-07	5756553	32308741	51	136
BIOCARTA CXCR4 PATHWAY	5.36E-08	5.02E-07	5756553	32308741	86	276
BIOCARTA STATHMIN PATHWAY	5.40E-08	5.02E-07	5756553	32308741	60	171
PID INSULIN PATHWAY	1.04E-07	9.57E-07	5756553	32308741	242	990
PID WNT CANONICAL PATHWAY	1.35E-07	1.24E-06	5756553	32308741	59	171
PID MAPK TRK PATHWAY	3.05E-07	2.77E-06	5756553	32308741	148	561
REACTOME ACTIVATED TLR4 SIGNALLING	3.12E-07	2.82E-06	5756553	32308741	803	3828
PID P75 NTR PATHWAY	3.38E-07	3.04E-06	5756553	32308741	513	2346
REACTOME INHIBITION OF INSULIN SECRETION BY ADRENALINE NORADRENALINE	3.67E-07	3.27E-06	5756553	32308741	89	300
REACTOME CA DEPENDENT EVENTS	4.35E-07	3.86E-06	5756553	32308741	113	406
REACTOME SIGNALING BY NOTCH1	5.08E-07	4.48E-06	5756553	32308741	498	2278
KEGG RENAL CELL CARCINOMA	5.16E-07	4.49E-06	5756553	32308741	525	2415
PID ILK PATHWAY	5.16E-07	4.49E-06	5756553	32308741	238	990
PID S1P S1P1 PATHWAY	5.69E-07	4.91E-06	5756553	32308741	67	210
REACTOME RECYCLING PATHWAY OF L1	6.21E-07	5.33E-06	5756553	32308741	94	325
REACTOME AMINE LIGAND BINDING RECEPTORS	8.02E-07	6.84E-06	5756553	32308741	161	630
BIOCARTA ERYTH PATHWAY	9.22E-07	7.77E-06	5756553	32308741	36	91
REACTOME MYD88 MAL CASCADE INITIATED ON PLASMA MEMBRANE	9.23E-07	7.77E-06	5756553	32308741	653	3081
REACTOME REGULATION OF GLUCOKINASE BY GLUCOKINASE REGULATORY PROTEIN	1.12E-06	9.38E-06	5756553	32308741	99	351
KEGG BASAL CELL CARCINOMA	1.16E-06	9.64E-06	5756553	32308741	326	1431
BIOCARTA PYK2 PATHWAY	1.19E-06	9.83E-06	5756553	32308741	105	378
PID HIF2PATHWAY	1.29E-06	1.05E-05	5756553	32308741	131	496
PID IL1 PATHWAY	1.29E-06	1.05E-05	5756553	32308741	131	496
PID LYSOPHOSPHOLIPID PATHWAY	1.35E-06	1.09E-05	5756553	32308741	468	2145
REACTOME INSULIN SYNTHESIS AND PROCESSING	1.40E-06	1.13E-05	5756553	32308741	61	190
REACTOME ACTIVATION OF RAC	1.42E-06	1.14E-05	5756553	32308741	32	78

REACTOME BMAL1 CLOCK NPAS2 ACTIVATES CIRCADIAN EXPRESSION	1.62E-06	1.29E-05	5756553	32308741	152	595
REACTOME ION TRANSPORT BY P TYPE ATPASES	2.11E-06	1.67E-05	5756553	32308741	137	528
REACTOME TANDEM PORE DOMAIN POTASSIUM CHANNELS	2.12E-06	1.67E-05	5756553	32308741	25	55
KEGG NON SMALL CELL LUNG CANCER	2.18E-06	1.70E-05	5756553	32308741	324	1431
PID RAC1 REG PATHWAY	2.54E-06	1.98E-05	5756553	32308741	174	703
REACTOME GABA A RECEPTOR ACTIVATION	2.88E-06	2.23E-05	5756553	32308741	28	66
PID WNT SIGNALING PATHWAY	3.72E-06	2.86E-05	5756553	32308741	97	351
PID IL8 CXCR1 PATHWAY	3.79E-06	2.89E-05	5756553	32308741	103	378
PID KIT PATHWAY	3.87E-06	2.94E-05	5756553	32308741	301	1326
BIOCARTA STRESS PATHWAY	5.00E-06	3.78E-05	5756553	32308741	85	300
KEGG BLADDER CANCER	5.73E-06	4.31E-05	5756553	32308741	205	861
BIOCARTA PDGF PATHWAY	5.90E-06	4.38E-05	5756553	32308741	128	496
PID NETRIN PATHWAY	5.90E-06	4.38E-05	5756553	32308741	128	496
REACTOME NOD1 2 SIGNALING PATHWAY	7.27E-06	5.37E-05	5756553	32308741	108	406
REACTOME VOLTAGE GATED POTASSIUM CHANNELS	7.36E-06	5.41E-05	5756553	32308741	213	903
KEGG GLYCOSPHINGOLIPID BIOSYNTHESIS LACTO AND NEOLACTO SERIES	7.40E-06	5.41E-05	5756553	32308741	90	325
REACTOME DOWNSTREAM SIGNAL TRANSDUCTION	7.44E-06	5.41E-05	5756553	32308741	838	4095
PID CMYB PATHWAY	7.86E-06	5.68E-05	5756553	32308741	721	3486
REACTOME G ALPHA1213 SIGNALLING EVENTS	8.34E-06	6.00E-05	5756553	32308741	555	2628
REACTOME INHIBITION OF VOLTAGE GATED CA2 CHANNELS VIA GBETA GAMMA SUBUNITS	9.25E-06	6.61E-05	5756553	32308741	84	300
PID SHP2 PATHWAY	1.04E-05	7.37E-05	5756553	32308741	363	1653
REACTOME PLATELET HOMEOSTASIS	1.04E-05	7.37E-05	5756553	32308741	597	2850
PID INTEGRIN5 PATHWAY	1.33E-05	9.36E-05	5756553	32308741	45	136
KEGG CELL ADHESION MOLECULES CAMS	1.39E-05	9.72E-05	5756553	32308741	1595	8128
ST WNT CA2 CYCLIC GMP PATHWAY	1.40E-05	9.74E-05	5756553	32308741	58	190
PID RHOA REG PATHWAY	1.47E-05	0.00010208	5756553	32308741	238	1035
REACTOME PROTEOLYTIC CLEAVAGE OF SNARE COMPLEX PROTEINS	1.56E-05	0.00010771	5756553	32308741	37	105
PID P73PATHWAY	1.76E-05	0.00012034	5756553	32308741	639	3081

PID RAC1 PATHWAY	1.99E-05	0.00013582	5756553	32308741	306	1378
REACTOME CHONDROITIN SULFATE BIOSYNTHESIS	2.00E-05	0.00013582	5756553	32308741	53	171
PID FAS PATHWAY	2.01E-05	0.0001359	5756553	32308741	169	703
BIOCARTA GSK3 PATHWAY	2.03E-05	0.00013643	5756553	32308741	94	351
BIOCARTA CHEMICAL PATHWAY	2.07E-05	0.00013863	5756553	32308741	67	231
BIOCARTA CELLCYCLE PATHWAY	2.12E-05	0.00014051	5756553	32308741	72	253
REACTOME RNA POL I TRANSCRIPTION INITIATION	2.12E-05	0.00014051	5756553	32308741	72	253
REACTOME MAP KINASE ACTIVATION IN TLR CASCADE	2.37E-05	0.00015577	5756553	32308741	265	1176
REACTOME DARPP 32 EVENTS	2.42E-05	0.00015828	5756553	32308741	77	276
KEGG GLIOMA	2.48E-05	0.00016168	5756553	32308741	431	2016
REACTOME INTEGRIN CELL SURFACE INTERACTIONS	2.59E-05	0.00016834	5756553	32308741	637	3081
PID P38 GAMMA DELTA PATHWAY	2.86E-05	0.00018491	5756553	32308741	23	55
REACTOME TRANS GOLGI NETWORK VESICLE BUDDING	3.00E-05	0.00019264	5756553	32308741	359	1653
REACTOME NRAGE SIGNALS DEATH THROUGH JNK	3.06E-05	0.00019564	5756553	32308741	209	903
REACTOME P75 NTR RECEPTOR MEDIATED SIGNALLING	3.15E-05	0.00020018	5756553	32308741	636	3081
REACTOME INHIBITION OF THE PROTEOLYTIC ACTIVITY OF APC C REQUIRED FOR THE ONSET OF ANAPHASE BY MITOTIC SPINDLE CHECKPOINT COMPONENTS	3.39E-05	0.00021443	5756553	32308741	48	153
REACTOME MITOTIC G2 G2 M PHASES	3.83E-05	0.0002414	5756553	32308741	620	3003
REACTOME ADENYLATE CYCLASE INHIBITORY PATHWAY	4.03E-05	0.00025311	5756553	32308741	29	78
REACTOME CGMP EFFECTS	4.21E-05	0.00026271	5756553	32308741	52	171
REACTOME RNA POL I TRANSCRIPTION TERMINATION	4.48E-05	0.00027851	5756553	32308741	61	210
KEGG COLORECTAL CANCER	4.57E-05	0.00028258	5756553	32308741	404	1891
KEGG PHOSPHATIDYLINOSITOL SIGNALING SYSTEM	5.23E-05	0.00032222	5756553	32308741	589	2850
REACTOME NITRIC OXIDE STIMULATES GUANYLATE CYCLASE	5.31E-05	0.00032556	5756553	32308741	81	300
KEGG TYPE II DIABETES MELLITUS	5.39E-05	0.00032885	5756553	32308741	234	1035
PID S1P S1P3 PATHWAY	5.58E-05	0.00033882	5756553	32308741	104	406
REACTOME INTERACTIONS OF VPR WITH HOST CELLULAR PROTEINS	6.05E-05	0.00036574	5756553	32308741	123	496
PID CDC42 REG PATHWAY	6.18E-05	0.00037162	5756553	32308741	110	435
REACTOME G2 M CHECKPOINTS	6.29E-05	0.00037705	5756553	32308741	190	820
SIG PIP3 SIGNALING IN CARDIAC MYOCTES	7.55E-05	0.00045054	5756553	32308741	464	2211

REACTOME G ALPHA S SIGNALLING EVENTS	7.76E-05	0.00046071	5756553	32308741	1287	6555
KEGG PEROXISOME	8.53E-05	0.00050442	5756553	32308741	601	2926
PID LYMPH ANGIOGENESIS PATHWAY	9.22E-05	0.00054231	5756553	32308741	80	300
REACTOME MTORC1 MEDIATED SIGNALLING	9.48E-05	0.00055536	5756553	32308741	22	55
SIG CHEMOTAXIS	9.91E-05	0.00057819	5756553	32308741	223	990
PID TNF PATHWAY	9.99E-05	0.00058027	5756553	32308741	232	1035
BIOCARTA TOLL PATHWAY	0.0001068	0.0006178	5756553	32308741	157	666
PID TCR JNK PATHWAY	0.000111	0.00063932	5756553	32308741	28	78
BIOCARTA GABA PATHWAY	0.0001192	0.00067925	5756553	32308741	19	45
BIOCARTA GPCR PATHWAY	0.0001195	0.00067925	5756553	32308741	135	561
REACTOME RECRUITMENT OF NUMA TO MITOTIC CENTROSOMES	0.0001192	0.00067925	5756553	32308741	19	45
KEGG ADHERENS JUNCTION	0.0001218	0.00068795	5756553	32308741	542	2628
SIG REGULATION OF THE ACTIN CYTOSKELETON BY RHO GTPASES	0.0001221	0.00068795	5756553	32308741	142	595
KEGG VASCULAR SMOOTH MUSCLE CONTRACTION	0.0001324	0.00074285	5756553	32308741	1178	5995
BIOCARTA NTHI PATHWAY	0.0001367	0.00076384	5756553	32308741	74	276
BIOCARTA IL10 PATHWAY	0.0001489	0.00082848	5756553	32308741	42	136
REACTOME PTM GAMMA CARBOXYLATION HYPUSINE FORMATION AND ARYLSULFATASE ACTIVATION	0.0001573	0.00087184	5756553	32308741	79	300
PID RET PATHWAY	0.0001724	0.00095123	5756553	32308741	171	741
REACTOME METABOLISM OF NUCLEOTIDES	0.0001833	0.00100754	5756553	32308741	499	2415
PID FRA PATHWAY	0.0001934	0.00105855	5756553	32308741	148	630
BIOCARTA WNT PATHWAY	0.0001958	0.00106729	5756553	32308741	84	325
REACTOME AMINO ACID AND OLIGOPEPTIDE SLC TRANSPORTERS	0.0002087	0.0011331	5756553	32308741	248	1128
PID SYNDECAN 4 PATHWAY	0.0002164	0.00117024	5756553	32308741	120	496
PID CDC42 PATHWAY	0.0002226	0.00119837	5756553	32308741	498	2415
PID LPA4 PATHWAY	0.0002261	0.00121231	5756553	32308741	34	105
REACTOME TOLL RECEPTOR CASCADES	0.0002345	0.00125246	5756553	32308741	1194	6105
REACTOME NCAM1 INTERACTIONS	0.0002423	0.00128888	5756553	32308741	170	741
PID IL8 CXCR2 PATHWAY	0.0002614	0.001385	5756553	32308741	133	561
PID AURORA A PATHWAY	0.0002694	0.00142159	5756553	32308741	113	465
PID MYC ACTIV PATHWAY	0.0002818	0.00148131	5756553	32308741	609	3003

BIOCARTA RB PATHWAY	0.0002901	0.00151885	5756553	32308741	27	78
REACTOME REGULATION OF WATER BALANCE BY RENAL AQUAPORINS	0.0002951	0.00153908	5756553	32308741	202	903
REACTOME GAP JUNCTION TRAFFICKING	0.0003208	0.00166674	5756553	32308741	83	325
KEGG THYROID CANCER	0.0003521	0.00182209	5756553	32308741	100	406
REACTOME HYALURONAN METABOLISM	0.0003635	0.0018664	5756553	32308741	30	91
REACTOME HIV LIFE CYCLE	0.0003625	0.0018664	5756553	32308741	1253	6441
REACTOME PLATELET CALCIUM HOMEOSTASIS	0.000368	0.00188245	5756553	32308741	37	120
REACTOME TRAF6 MEDIATED INDUCTION OF NFKB AND MAP KINASES UPON TLR7 8 OR 9 ACTIVATION	0.0003706	0.0018885	5756553	32308741	536	2628
REACTOME ADENYLATE CYCLASE ACTIVATING PATHWAY	0.0004002	0.00203139	5756553	32308741	18	45
ST GRANULE CELL SURVIVAL PATHWAY	0.0004162	0.00210491	5756553	32308741	88	351
REACTOME KERATAN SULFATE BIOSYNTHESIS	0.0005178	0.00260845	5756553	32308741	82	325
REACTOME INTERACTION BETWEEN L1 AND ANKYRINS	0.0005333	0.00267667	5756553	32308741	57	210
SIG CD40PATHWAYMAP	0.000551	0.00275491	5756553	32308741	131	561
KEGG TASTE TRANSDUCTION	0.0005767	0.00287274	5756553	32308741	217	990
BIOCARTA CARDIACEGF PATHWAY	0.0005971	0.00296311	5756553	32308741	44	153
REACTOME INWARDLY RECTIFYING K CHANNELS	0.0006059	0.00299594	5756553	32308741	111	465
BIOCARTA NO2IL12 PATHWAY	0.0006344	0.00310221	5756553	32308741	40	136
REACTOME APC C CDC20 MEDIATED DEGRADATION OF CYCLIN B	0.0006316	0.00310221	5756553	32308741	48	171
REACTOME SYNTHESIS OF GLYCOSYLPHOSPHATIDYLINOSITOL GPI	0.0006344	0.00310221	5756553	32308741	40	136
PID ECADHERIN STABILIZATION PATHWAY	0.0006439	0.00313714	5756553	32308741	191	861
REACTOME G2 M DNA DAMAGE CHECKPOINT	0.000721	0.0034997	5756553	32308741	15	36
BIOCARTA RACCYCD PATHWAY	0.0008231	0.00395963	5756553	32308741	81	325
ST T CELL SIGNAL TRANSDUCTION	0.0008247	0.00395963	5756553	32308741	207	946
PID ER NONGENOMIC PATHWAY	0.0008228	0.00395963	5756553	32308741	182	820
BIOCARTA NO1 PATHWAY	0.0008283	0.00396275	5756553	32308741	104	435
REACTOME TRANSPORT OF MATURE MRNA DERIVED FROM AN INTRONLESS TRANSCRIPT	0.0008682	0.00413865	5756553	32308741	123	528
REACTOME APC CDC20 MEDIATED DEGRADATION OF NEK2A	0.0009371	0.00445111	5756553	32308741	56	210
REACTOME GLUTAMATE NEUROTRANSMITTER RELEASE CYCLE	0.0010961	0.00518801	5756553	32308741	32	105

KEGG NITROGEN METABOLISM	0.0011189	0.00523973	5756553	32308741	65	253
PID CONE PATHWAY	0.0011189	0.00523973	5756553	32308741	65	253
PID TOLL ENDOGENOUS PATHWAY	0.0011189	0.00523973	5756553	32308741	65	253
BIOCARTA TNFR2 PATHWAY	0.0011371	0.00526935	5756553	32308741	43	153
PID VEGFR1 PATHWAY	0.0011369	0.00526935	5756553	32308741	75	300
REACTOME SMAD2 SMAD3 SMAD4 HETEROTRIMER REGULATES TRANSCRIPTION	0.0011369	0.00526935	5756553	32308741	75	300
REACTOME KERATAN SULFATE KERATIN METABOLISM	0.0012258	0.00566084	5756553	32308741	103	435
PID CD40 PATHWAY	0.0013045	0.00596236	5756553	32308741	109	465
PID P38 ALPHA BETA PATHWAY	0.0013045	0.00596236	5756553	32308741	109	465
PID CERAMIDE PATHWAY	0.0013033	0.00596236	5756553	32308741	241	1128
REACTOME HS GAG DEGRADATION	0.001313	0.00598065	5756553	32308741	51	190
PID P38 ALPHA BETA DOWNSTREAM PATHWAY	0.0013523	0.00611758	5756553	32308741	157	703
REACTOME NETRIN1 SIGNALING	0.0013523	0.00611758	5756553	32308741	157	703
PID ERBB1 RECEPTOR PROXIMAL PATHWAY	0.0015018	0.00677105	5756553	32308741	135	595
REACTOME ADP SIGNALLING THROUGH P2RY12	0.0016099	0.00723373	5756553	32308741	55	210
BIOCARTA VDR PATHWAY	0.0017801	0.00794491	5756553	32308741	22	66
REACTOME REVERSIBLE HYDRATION OF CARBON DIOXIDE	0.0017801	0.00794491	5756553	32308741	22	66
BIOCARTA NUCLEARRS PATHWAY	0.0018492	0.00817095	5756553	32308741	28	91
REACTOME TGF BETA RECEPTOR SIGNALING IN EMT EPITHELIAL TO MESENCHYMAL TRANSITION	0.0018492	0.00817095	5756553	32308741	28	91
REACTOME LATE PHASE OF HIV LIFE CYCLE	0.0018461	0.00817095	5756553	32308741	980	5050
BIOCARTA SPPA PATHWAY	0.002113	0.0093057	5756553	32308741	59	231
KEGG SNARE INTERACTIONS IN VESICULAR TRANSPORT	0.0022069	0.00965507	5756553	32308741	148	666
ST GA13 PATHWAY	0.0022069	0.00965507	5756553	32308741	148	666
REACTOME DIABETES PATHWAYS	0.0022321	0.00973341	5756553	32308741	1455	7626
BIOCARTA TALL1 PATHWAY	0.002269	0.00986209	5756553	32308741	31	105
REACTOME MYOGENESIS	0.0023521	0.01015678	5756553	32308741	84	351
REACTOME TRANSPORT OF RIBONUCLEOPROTEINS INTO THE HOST NUCLEUS	0.0023521	0.01015678	5756553	32308741	84	351
BIOCARTA CK1 PATHWAY	0.0023715	0.0102075	5756553	32308741	38	136

PID PS1 PATHWAY	0.0026058	0.01117957	5756553	32308741	220	1035
PID S1P META PATHWAY	0.0027041	0.01156413	5756553	32308741	54	210
PID RHODOPSIN PATHWAY	0.0027353	0.01162263	5756553	32308741	68	276
PID S1P S1P2 PATHWAY	0.0027353	0.01162263	5756553	32308741	68	276
KEGG RNA DEGRADATION	0.0027494	0.01164537	5756553	32308741	328	1596
REACTOME SIGNALING BY ERBB2	0.0028343	0.0119672	5756553	32308741	903	4656
REACTOME G0 AND EARLY G1	0.0029293	0.01232884	5756553	32308741	63	253
REACTOME SIGNALING BY SCF KIT	0.003001	0.01259074	5756553	32308741	551	2775
REACTOME ACTIVATION OF BH3 ONLY PROTEINS	0.0030425	0.01272493	5756553	32308741	34	120
REACTOME SIGNALING BY BMP	0.0034334	0.01431497	5756553	32308741	58	231
REACTOME MAPK TARGETS NUCLEAR EVENTS MEDIATED BY MAP KINASES	0.0037027	0.01538934	5756553	32308741	100	435
PID AVB3 OPN PATHWAY	0.0037933	0.01571668	5756553	32308741	106	465
REACTOME DEADENYLATION DEPENDENT MRNA DECAY	0.0038905	0.01606955	5756553	32308741	201	946
PID ATF2 PATHWAY	0.0039314	0.01618809	5756553	32308741	337	1653
KEGG PROGESTERONE MEDIATED OOCYTE MATURATION	0.0040825	0.01675843	5756553	32308741	682	3486
BIOCARTA BCR PATHWAY	0.0041304	0.01690276	5756553	32308741	125	561
PID INTEGRIN A9B1 PATHWAY	0.0042201	0.01721684	5756553	32308741	72	300
REACTOME GAP JUNCTION ASSEMBLY	0.0043624	0.01774299	5756553	32308741	37	136
BIOCARTA CD40 PATHWAY	0.0045052	0.01818425	5756553	32308741	30	105
PID GLYPICAN 1PATHWAY	0.0045119	0.01818425	5756553	32308741	77	325
PID RXR VDR PATHWAY	0.0045119	0.01818425	5756553	32308741	77	325
BIOCARTA MTOR PATHWAY	0.004605	0.01850363	5756553	32308741	62	253
PID BETA CATENIN NUC PATHWAY	0.0046958	0.01881164	5756553	32308741	620	3160
BIOCARTA EGFR SMRTE PATHWAY	0.0055548	0.02218575	5756553	32308741	18	55
PID TRAIL PATHWAY	0.0061578	0.02452073	5756553	32308741	87	378
KEGG PURINE METABOLISM	0.0061809	0.02453906	5756553	32308741	2204	11781
SA G2 AND M PHASES	0.0062018	0.02454893	5756553	32308741	11	28
BIOCARTA IL1R PATHWAY	0.0063821	0.02518744	5756553	32308741	117	528
KEGG NOD LIKE RECEPTOR SIGNALING PATHWAY	0.0068186	0.02675149	5756553	32308741	379	1891
REACTOME O LINKED GLYCOSYLATION OF MUCINS	0.0068035	0.02675149	5756553	32308741	302	1485

KEGG FC GAMMA R MEDIATED PHAGOCYTOSIS	0.0068584	0.02682846	5756553	32308741	791	4095
BIOCARTA PAR1 PATHWAY	0.0068955	0.02689433	5756553	32308741	144	666
PID ANGIOPOIETIN RECEPTOR PATHWAY	0.0072755	0.02820147	5756553	32308741	252	1225
PID RETINOIC ACID PATHWAY	0.0072942	0.02820147	5756553	32308741	98	435
REACTOME ACTIVATION OF CHAPERONE GENES BY XBP1S	0.007271	0.02820147	5756553	32308741	190	903
REACTOME NEP NS2 INTERACTS WITH THE CELLULAR EXPORT MACHINERY	0.0074199	0.02860425	5756553	32308741	81	351
PID EPHB FWD PATHWAY	0.0074522	0.02864564	5756553	32308741	166	780
REACTOME NEUROTRANSMITTER RELEASE CYCLE	0.0075023	0.02875504	5756553	32308741	123	561
REACTOME NFKB AND MAP KINASES ACTIVATION MEDIATED BY TLR4 SIGNALING REPERTOIRE	0.0078546	0.03001917	5756553	32308741	451	2278
BIOCARTA ARENRF2 PATHWAY	0.0079307	0.03022283	5756553	32308741	23	78
REACTOME MITOTIC G1 G1 S PHASES	0.0082423	0.03132074	5756553	32308741	1579	8385
REACTOME CYCLIN A B1 ASSOCIATED EVENTS DURING G2 M TRANSITION	0.0085766	0.0324984	5756553	32308741	29	105
REACTOME SIGNALLING TO ERKS	0.009156	0.034595	5756553	32308741	129	595
REACTOME SIGNALING BY NOTCH3	0.0092736	0.03494013	5756553	32308741	20	66
REACTOME ACTIVATED NOTCH1 TRANSMITS SIGNAL TO THE NUCLEUS	0.0096331	0.03619215	5756553	32308741	75	325
REACTOME FACTORS INVOLVED IN MEGAKARYOCYTE DEVELOPMENT AND PLATELET PRODUCTION	0.0102754	0.03849665	5756553	32308741	1348	7140
BIOCARTA CDMAC PATHWAY	0.0103318	0.03859896	5756553	32308741	32	120
REACTOME FATTY ACYL COA BIOSYNTHESIS	0.0110845	0.04129504	5756553	32308741	39	153
PID FGF PATHWAY	0.0111173	0.04130159	5756553	32308741	289	1431
REACTOME CIRCADIAN CLOCK	0.0118582	0.0439315	5756553	32308741	259	1275
REACTOME DNA REPLICATION	0.0122578	0.04528591	5756553	32308741	3213	17391
REACTOME SIGNAL TRANSDUCTION BY L1	0.0131262	0.04835981	5756553	32308741	121	561
PID ECADHERIN NASCENT AJ PATHWAY	0.0133791	0.04915516	5756553	32308741	156	741
PID AR NONGENOMIC PATHWAY	0.0134764	0.04937628	5756553	32308741	102	465

Note:

p value: hypergeometric p value

q value: FDR adjusted p value

n: number of background gene pairs

k: number of interesting gene pairs

m: number of background gene pairs in a pathway

x： number of interesting gene pairs in a pathway

PMID: PubMed ID number.

Pathways are ranked according to q values, with smaller q values listed at the beginning, bigger q values listed at the end.

MSigDB pathways enriched with DR gene pairs identified from $GBM_{34\text{-}13}$

Pathway Name	p value	q value	k	n	x	m	PMID
KEGG PURINE METABOLISM	0.000	0.000	3659102	32308741	1696	11781	2154328
KEGG MAPK SIGNALING PATHWAY	0.000	0.000	3659102	32308741	5994	34191	26794430
KEGG ERBB SIGNALING PATHWAY	0.000	0.000	3659102	32308741	679	3655	25313012
KEGG CALCIUM SIGNALING PATHWAY	0.000	0.000	3659102	32308741	2821	15225	26826650
KEGG P53 SIGNALING PATHWAY	0.000	0.000	3659102	32308741	477	2278	26986934
KEGG CELL CYCLE	0.000	0.000	3659102	32308741	1412	7503	26983952
KEGG WNT SIGNALING PATHWAY	0.000	0.000	3659102	32308741	1708	10878	26979081
KEGG FOCAL ADHESION	0.000	0.000	3659102	32308741	2803	19503	25504636
KEGG GAP JUNCTION	0.000	0.000	3659102	32308741	681	3828	18286483
KEGG GNRH SIGNALING PATHWAY	0.000	0.000	3659102	32308741	775	4656	19360304
KEGG NEUROACTIVE LIGAND RECEPTOR INTERACTION	0.000	0.000	3659102	32308741	5811	35778	
KEGG OOCYTE MEIOSIS	0.000	0.000	3659102	32308741	1009	6105	
KEGG ENDOCYTOSIS	0.000	0.000	3659102	32308741	2261	16110	
KEGG AXON GUIDANCE	0.000	0.000	3659102	32308741	1371	8256	
KEGG LONG TERM POTENTIATION	0.000	0.000	3659102	32308741	516	2415	
KEGG LONG TERM DEPRESSION	0.000	0.000	3659102	32308741	450	2145	
KEGG PROGESTERONE MEDIATED OOCYTE MATURATION	0.000	0.000	3659102	32308741	606	3486	
KEGG MELANOGENESIS	0.000	0.000	3659102	32308741	760	4950	
KEGG AMYOTROPHIC LATERAL SCLEROSIS ALS	0.000	0.000	3659102	32308741	318	1378	
KEGG PATHWAYS IN CANCER	0.000	0.000	3659102	32308741	6547	52326	
KEGG GLIOMA	0.000	0.000	3659102	32308741	364	2016	
KEGG HYPERTROPHIC CARDIOMYOPATHY HCM	0.000	0.000	3659102	32308741	706	3403	
KEGG ARRHYTHMOGENIC RIGHT VENTRICULAR CARDIOMYOPATHY ARVC	0.000	0.000	3659102	32308741	490	2701	
KEGG DILATED CARDIOMYOPATHY	0.000	0.000	3659102	32308741	884	4005	
BIOCARTA MAPK PATHWAY	0.000	0.000	3659102	32308741	585	3655	
ST INTEGRIN SIGNALING PATHWAY	0.000	0.000	3659102	32308741	664	3321	
PID ENDOTHELIN PATHWAY	0.000	0.000	3659102	32308741	399	1953	

PID AURORA B PATHWAY	0.000	0.000	3659102	32308741	214	741
PID NOTCH PATHWAY	0.000	0.000	3659102	32308741	325	1711
PID P73PATHWAY	0.000	0.000	3659102	32308741	516	3081
PID WNT NONCANONICAL PATHWAY	0.000	0.000	3659102	32308741	152	496
PID E2F PATHWAY	0.000	0.000	3659102	32308741	543	2701
PID PLK1 PATHWAY	0.000	0.000	3659102	32308741	310	1035
PID FOXM1 PATHWAY	0.000	0.000	3659102	32308741	222	780
PID TRKR PATHWAY	0.000	0.000	3659102	32308741	355	1830
PID AURORA A PATHWAY	0.000	0.000	3659102	32308741	120	465
REACTOME SIGNALING BY RHO GTPASES	0.000	0.000	3659102	32308741	959	5886
REACTOME SIGNALLING BY NGF	0.000	0.000	3659102	32308741	3448	21945
REACTOME DEVELOPMENTAL BIOLOGY	0.000	0.000	3659102	32308741	10843	72010
REACTOME CELL CYCLE	0.000	0.000	3659102	32308741	11840	75466
REACTOME SIGNALING BY EGFR IN CANCER	0.000	0.000	3659102	32308741	834	5460
REACTOME NGF SIGNALLING VIA TRKA FROM THE PLASMA MEMBRANE	0.000	0.000	3659102	32308741	1424	8646
REACTOME HS GAG BIOSYNTHESIS	0.000	0.000	3659102	32308741	127	435
REACTOME HEPARAN SULFATE HEPARIN HS GAG METABOLISM	0.000	0.000	3659102	32308741	328	1275
REACTOME GLYCOSAMINOGLYCAN METABOLISM	0.000	0.000	3659102	32308741	1167	5778
REACTOME MHC CLASS II ANTIGEN PRESENTATION	0.000	0.000	3659102	32308741	662	3741
REACTOME TRANSMISSION ACROSS CHEMICAL SYNAPSES	0.000	0.000	3659102	32308741	4100	16471
REACTOME NEURONAL SYSTEM	0.000	0.000	3659102	32308741	8347	36856
REACTOME CELL CYCLE MITOTIC	0.000	0.000	3659102	32308741	8287	46971
REACTOME OPIOID SIGNALLING	0.000	0.000	3659102	32308741	546	2926
REACTOME NEUROTRANSMITTER RECEPTOR BINDING AND DOWNSTREAM TRANSMISSION IN THE POSTSYNAPTIC CELL	0.000	0.000	3659102	32308741	2377	8778
REACTOME PLC BETA MEDIATED EVENTS	0.000	0.000	3659102	32308741	187	861
REACTOME TRANSMEMBRANE TRANSPORT OF SMALL MOLECULES	0.000	0.000	3659102	32308741	10780	81003
REACTOME SIGNALING BY PDGF	0.000	0.000	3659102	32308741	1326	6903
REACTOME G1 S TRANSITION	0.000	0.000	3659102	32308741	856	5565
REACTOME AXON GUIDANCE	0.000	0.000	3659102	32308741	5221	28680

REACTOME NCAM SIGNALING FOR NEURITE OUT GROWTH	0.000	0.000	3659102	32308741	429	2016
REACTOME G ALPHA Z SIGNALLING EVENTS	0.000	0.000	3659102	32308741	239	946
REACTOME ACTIVATION OF NMDA RECEPTOR UPON GLUTAMATE BINDING AND POSTSYNAPTIC EVENTS	0.000	0.000	3659102	32308741	187	666
REACTOME CREB PHOSPHORYLATION THROUGH THE ACTIVATION OF RAS	0.000	0.000	3659102	32308741	103	351
REACTOME POST NMDA RECEPTOR ACTIVATION EVENTS	0.000	0.000	3659102	32308741	156	528
REACTOME MITOTIC G1 G1 S PHASES	0.000	0.000	3659102	32308741	1367	8385
REACTOME MITOTIC M M G1 PHASES	0.000	0.000	3659102	32308741	2333	13861
REACTOME L1CAM INTERACTIONS	0.000	0.000	3659102	32308741	711	3403
REACTOME GABA B RECEPTOR ACTIVATION	0.000	0.000	3659102	32308741	189	703
REACTOME GABA RECEPTOR ACTIVATION	0.000	0.000	3659102	32308741	459	1326
REACTOME ION CHANNEL TRANSPORT	0.000	0.000	3659102	32308741	357	1431
REACTOME LIGAND GATED ION CHANNEL TRANSPORT	0.000	0.000	3659102	32308741	71	210
REACTOME DNA REPLICATION	0.000	0.000	3659102	32308741	2962	17391
REACTOME E2F MEDIATED REGULATION OF DNA REPLICATION	0.000	0.000	3659102	32308741	149	528
REACTOME METABOLISM OF CARBOHYDRATES	0.000	0.000	3659102	32308741	3441	26335
REACTOME HEMOSTASIS	0.000	0.000	3659102	32308741	12515	97020
REACTOME MITOTIC PROMETAPHASE	0.000	0.000	3659102	32308741	872	3570
REACTOME G1 S SPECIFIC TRANSCRIPTION	0.000	0.000	3659102	32308741	54	136
REACTOME POTASSIUM CHANNELS	0.000	0.000	3659102	32308741	844	4656
NABA ECM GLYCOPROTEINS	0.000	0.000	3659102	32308741	2621	17766
NABA CORE MATRISOME	0.000	0.000	3659102	32308741	4953	35778
PID THROMBIN PAR1 PATHWAY	1.11E-16	1.87E-15	3659102	32308741	189	903
REACTOME NCAM1 INTERACTIONS	1.11E-16	1.87E-15	3659102	32308741	163	741
REACTOME TRAFFICKING OF AMPA RECEPTORS	2.22E-16	3.69E-15	3659102	32308741	96	351
KEGG ACUTE MYELOID LEUKEMIA	4.44E-16	7.29E-15	3659102	32308741	291	1596
BIOCARTA STATHMIN PATHWAY	1.33E-15	2.13E-14	3659102	32308741	59	171
BIOCARTA CREB PATHWAY	1.33E-15	2.13E-14	3659102	32308741	94	351
REACTOME G2 M CHECKPOINTS	1.55E-15	2.46E-14	3659102	32308741	172	820
REACTOME SIGNALING BY ROBO RECEPTOR	7.88E-15	1.23E-13	3659102	32308741	102	406

REACTOME CELL CELL COMMUNICATION	1.28E-14	1.97E-13	3659102	32308741	975	6786
KEGG VASCULAR SMOOTH MUSCLE CONTRACTION	1.47E-14	2.24E-13	3659102	32308741	873	5995
KEGG DNA REPLICATION	2.84E-14	4.30E-13	3659102	32308741	138	630
REACTOME INHIBITION OF THE PROTEOLYTIC ACTIVITY OF APC C REQUIRED FOR THE ONSET OF ANAPHASE BY MITOTIC SPINDLE CHECKPOINT COMPONENTS	2.90E-14	4.33E-13	3659102	32308741	53	153
BIOCARTA G2 PATHWAY	4.27E-14	6.32E-13	3659102	32308741	77	276
BIOCARTA TNFR1 PATHWAY	5.43E-14	7.93E-13	3659102	32308741	100	406
PID LIS1 PATHWAY	6.20E-14	8.96E-13	3659102	32308741	95	378
REACTOME TOLL RECEPTOR CASCADES	1.98E-13	2.83E-12	3659102	32308741	878	6105
REACTOME SYNTHESIS AND INTERCONVERSION OF NUCLEOTIDE DI AND TRIPHOSPHATES	2.06E-13	2.92E-12	3659102	32308741	48	136
ST JNK MAPK PATHWAY	2.10E-13	2.95E-12	3659102	32308741	159	780
KEGG ECM RECEPTOR INTERACTION	4.71E-13	6.53E-12	3659102	32308741	535	3486
PID NFAT 3PATHWAY	5.02E-13	6.89E-12	3659102	32308741	254	1431
REACTOME KINESINS	9.01E-13	1.22E-11	3659102	32308741	70	253
SIG CHEMOTAXIS	1.35E-12	1.82E-11	3659102	32308741	188	990
ST FAS SIGNALING PATHWAY	1.42E-12	1.89E-11	3659102	32308741	334	2016
REACTOME ACTIVATED TLR4 SIGNALLING	1.90E-12	2.51E-11	3659102	32308741	576	3828
REACTOME APC CDC20 MEDIATED DEGRADATION OF NEK2A	2.60E-12	3.39E-11	3659102	32308741	61	210
BIOCARTA KERATINOCYTE PATHWAY	3.69E-12	4.76E-11	3659102	32308741	193	1035
REACTOME ADHERENS JUNCTIONS INTERACTIONS	3.95E-12	5.05E-11	3659102	32308741	86	351
KEGG NEUROTROPHIN SIGNALING PATHWAY	4.71E-12	5.97E-11	3659102	32308741	1074	7750
REACTOME UNBLOCKING OF NMDA RECEPTOR GLUTAMATE BINDING AND ACTIVATION	5.66E-12	7.10E-11	3659102	32308741	39	105
PID IFNG PATHWAY	6.49E-12	8.07E-11	3659102	32308741	154	780
REACTOME SIGNALING BY FGFR IN DISEASE	8.39E-12	1.03E-10	3659102	32308741	1010	7260
REACTOME TRANSPORT OF INORGANIC CATIONS ANIONS AND AMINO ACIDS OLIGOPEPTIDES	9.08E-12	1.11E-10	3659102	32308741	606	4095
BIOCARTA G1 PATHWAY	1.71E-11	2.06E-10	3659102	32308741	89	378
BIOCARTA FAS PATHWAY	2.52E-11	3.02E-10	3659102	32308741	98	435

ST ADRENERGIC	3.80E-11	4.47E-10	3659102	32308741	123	595
REACTOME MYD88 MAL CASCADE INITIATED ON PLASMA MEMBRANE	3.80E-11	4.47E-10	3659102	32308741	469	3081
BIOCARTA BIOPEPTIDES PATHWAY	4.91E-11	5.73E-10	3659102	32308741	163	861
REACTOME INWARDLY RECTIFYING K CHANNELS	5.17E-11	5.97E-10	3659102	32308741	102	465
REACTOME CA DEPENDENT EVENTS	7.16E-11	8.21E-10	3659102	32308741	92	406
ST G ALPHA I PATHWAY	7.81E-11	8.88E-10	3659102	32308741	122	595
PID TXA2PATHWAY	8.16E-11	9.20E-10	3659102	32308741	267	1596
REACTOME INHIBITION OF VOLTAGE GATED CA2 CHANNELS VIA GBETA GAMMA SUBUNITS	8.37E-11	9.35E-10	3659102	32308741	74	300
REACTOME CELL CYCLE CHECKPOINTS	1.09E-10	1.20E-09	3659102	32308741	854	6105
REACTOME G ALPHA I SIGNALLING EVENTS	1.16E-10	1.28E-09	3659102	32308741	2195	17020
REACTOME METABOLISM OF NUCLEOTIDES	1.41E-10	1.53E-09	3659102	32308741	377	2415
ST MYOCYTE AD PATHWAY	1.54E-10	1.66E-09	3659102	32308741	82	351
PID RAS PATHWAY	1.66E-10	1.78E-09	3659102	32308741	91	406
PID TNF PATHWAY	2.03E-10	2.16E-09	3659102	32308741	186	1035
BIOCARTA CCR5 PATHWAY	2.11E-10	2.22E-09	3659102	32308741	43	136
REACTOME AMINO ACID AND OLIGOPEPTIDE SLC TRANSPORTERS	2.44E-10	2.55E-09	3659102	32308741	199	1128
REACTOME CHONDROITIN SULFATE DERMATAN SULFATE METABOLISM	2.83E-10	2.94E-09	3659102	32308741	192	1081
REACTOME DOWNSTREAM SIGNAL TRANSDUCTION	4.54E-10	4.68E-09	3659102	32308741	593	4095
REACTOME PLATELET HOMEOSTASIS	7.25E-10	7.42E-09	3659102	32308741	430	2850
REACTOME CELL JUNCTION ORGANIZATION	1.02E-09	1.03E-08	3659102	32308741	429	2850
BIOCARTA CELLCYCLE PATHWAY	1.32E-09	1.32E-08	3659102	32308741	63	253
PID LYSOPHOSPHOLIPID PATHWAY	1.32E-09	1.32E-08	3659102	32308741	335	2145
REACTOME GPCR LIGAND BINDING	1.71E-09	1.70E-08	3659102	32308741	8618	71631
REACTOME CREB PHOSPHORYLATION THROUGH THE ACTIVATION OF CAMKII	2.00E-09	1.97E-08	3659102	32308741	35	105
KEGG NOTCH SIGNALING PATHWAY	2.30E-09	2.25E-08	3659102	32308741	188	1081
REACTOME BOTULINUM NEUROTOXICITY	2.68E-09	2.60E-08	3659102	32308741	41	136
PID IL8 CXCR1 PATHWAY	2.80E-09	2.70E-08	3659102	32308741	83	378

REACTOME G0 AND EARLY G1	3.46E-09	3.31E-08	3659102	32308741	62	253
SIG PIP3 SIGNALING IN CARDIAC MYOCTES	3.74E-09	3.55E-08	3659102	32308741	341	2211
PID P53 DOWNSTREAM PATHWAY	4.27E-09	4.03E-08	3659102	32308741	1219	9180
BIOCARTA AGR PATHWAY	5.61E-09	5.26E-08	3659102	32308741	121	630
KEGG TYPE II DIABETES MELLITUS	8.16E-09	7.59E-08	3659102	32308741	179	1035
PID P38 GAMMA DELTA PATHWAY	8.39E-09	7.75E-08	3659102	32308741	23	55
PID IL1 PATHWAY	8.94E-09	8.20E-08	3659102	32308741	100	496
REACTOME HYALURONAN METABOLISM	9.04E-09	8.21E-08	3659102	32308741	31	91
REACTOME RAS ACTIVATION UOPN CA2 INFUX THROUGH NMDA RECEPTOR	9.08E-09	8.21E-08	3659102	32308741	40	136
REACTOME DAG AND IP3 SIGNALING	1.04E-08	9.38E-08	3659102	32308741	95	465
BIOCARTA GSK3 PATHWAY	1.06E-08	9.42E-08	3659102	32308741	77	351
BIOCARTA RB PATHWAY	1.25E-08	1.11E-07	3659102	32308741	28	78
KEGG PYRIMIDINE METABOLISM	1.63E-08	1.44E-07	3659102	32308741	615	4371
PID MET PATHWAY	1.89E-08	1.65E-07	3659102	32308741	460	3160
REACTOME RECRUITMENT OF NUMA TO MITOTIC CENTROSOMES	2.23E-08	1.94E-07	3659102	32308741	20	45
REACTOME CELL CELL JUNCTION ORGANIZATION	3.41E-08	2.94E-07	3659102	32308741	238	1485
PID S1P S1P4 PATHWAY	3.65E-08	3.13E-07	3659102	32308741	30	91
PID MYC PATHWAY	4.38E-08	3.74E-07	3659102	32308741	67	300
REACTOME APC C CDC20 MEDIATED DEGRADATION OF CYCLIN B	4.78E-08	4.05E-07	3659102	32308741	45	171
BIOCARTA CACAM PATHWAY	5.45E-08	4.56E-07	3659102	32308741	27	78
REACTOME ADENYLATE CYCLASE INHIBITORY PATHWAY	5.45E-08	4.56E-07	3659102	32308741	27	78
BIOCARTA P38MAPK PATHWAY	7.11E-08	5.91E-07	3659102	32308741	133	741
PID TGFBR PATHWAY	1.03E-07	8.49E-07	3659102	32308741	228	1431
REACTOME MAP KINASE ACTIVATION IN TLR CASCADE	1.16E-07	9.56E-07	3659102	32308741	193	1176
PID RAC1 REG PATHWAY	1.65E-07	1.35E-06	3659102	32308741	126	703
REACTOME SIGNALING BY ERBB2	2.24E-07	1.82E-06	3659102	32308741	640	4656
REACTOME RECYCLING PATHWAY OF L1	4.66E-07	3.75E-06	3659102	32308741	68	325
PID NETRIN PATHWAY	4.70E-07	3.77E-06	3659102	32308741	94	496
ST WNT CA2 CYCLIC GMP PATHWAY	4.80E-07	3.83E-06	3659102	32308741	46	190

REACTOME A TETRASACCHARIDE LINKER SEQUENCE IS REQUIRED FOR GAG SYNTHESIS	4.87E-07	3.84E-06	3659102	32308741	64	300
REACTOME INHIBITION OF INSULIN SECRETION BY ADRENALINE NORADRENALINE	4.87E-07	3.84E-06	3659102	32308741	64	300
PID PI3K PLC TRK PATHWAY	6.56E-07	5.14E-06	3659102	32308741	113	630
REACTOME PEPTIDE LIGAND BINDING RECEPTORS	6.81E-07	5.30E-06	3659102	32308741	1875	14878
REACTOME MITOTIC G2 G2 M PHASES	7.30E-07	5.65E-06	3659102	32308741	427	3003
REACTOME ADENYLATE CYCLASE ACTIVATING PATHWAY	7.64E-07	5.88E-06	3659102	32308741	18	45
KEGG CELL ADHESION MOLECULES CAMS	7.84E-07	5.99E-06	3659102	32308741	1061	8128
REACTOME ACTIVATION OF THE PRE REPLICATIVE COMPLEX	8.35E-07	6.35E-06	3659102	32308741	84	435
PID ER NONGENOMIC PATHWAY	1.05E-06	7.95E-06	3659102	32308741	139	820
BIOCARTA PYK2 PATHWAY	1.08E-06	8.15E-06	3659102	32308741	75	378
KEGG MELANOMA	1.23E-06	9.16E-06	3659102	32308741	359	2485
PID REELIN PATHWAY	1.27E-06	9.45E-06	3659102	32308741	79	406
REACTOME INTERACTION BETWEEN L1 AND ANKYRINS	1.63E-06	1.20E-05	3659102	32308741	48	210
REACTOME GABA A RECEPTOR ACTIVATION	1.87E-06	1.37E-05	3659102	32308741	22	66
BIOCARTA MCM PATHWAY	2.34E-06	1.70E-05	3659102	32308741	38	153
REACTOME LYSOSOME VESICLE BIOGENESIS	2.33E-06	1.70E-05	3659102	32308741	51	231
SA PTEN PATHWAY	2.41E-06	1.74E-05	3659102	32308741	35	136
PID WNT CANONICAL PATHWAY	2.52E-06	1.81E-05	3659102	32308741	41	171
PID VEGFR1 2 PATHWAY	3.07E-06	2.20E-05	3659102	32308741	338	2346
PID FGF PATHWAY	3.24E-06	2.31E-05	3659102	32308741	219	1431
KEGG CHEMOKINE SIGNALING PATHWAY	5.43E-06	3.84E-05	3659102	32308741	1983	15931
REACTOME DOWNSTREAM SIGNALING OF ACTIVATED FGFR	5.70E-06	3.99E-05	3659102	32308741	625	4656
REACTOME ACTIVATION OF ATR IN RESPONSE TO REPLICATION STRESS	5.69E-06	3.99E-05	3659102	32308741	104	595
PID IL8 CXCR2 PATHWAY	6.20E-06	4.32E-05	3659102	32308741	99	561
KEGG NON SMALL CELL LUNG CANCER	6.60E-06	4.57E-05	3659102	32308741	217	1431
REACTOME FACTORS INVOLVED IN MEGAKARYOCYTE DEVELOPMENT AND PLATELET PRODUCTION	7.84E-06	5.40E-05	3659102	32308741	927	7140

KEGG GLYCOSPHINGOLIPID BIOSYNTHESIS LACTO AND NEOLACTO SERIES	8.08E-06	5.51E-05	3659102	32308741	64	325
BIOCARTA WNT PATHWAY	8.08E-06	5.51E-05	3659102	32308741	64	325
PID RHOA PATHWAY	1.10E-05	7.48E-05	3659102	32308741	157	990
PID LPA4 PATHWAY	1.14E-05	7.58E-05	3659102	32308741	28	105
PID PS1 PATHWAY	1.12E-05	7.58E-05	3659102	32308741	163	1035
REACTOME CYCLIN A B1 ASSOCIATED EVENTS DURING G2 M TRANSITION	1.14E-05	7.58E-05	3659102	32308741	28	105
REACTOME ACTIVATION OF RAC	1.14E-05	7.58E-05	3659102	32308741	23	78
PID WNT SIGNALING PATHWAY	1.46E-05	9.68E-05	3659102	32308741	67	351
REACTOME TRAF6 MEDIATED INDUCTION OF NFKB AND MAP KINASES UPON TLR7 8 OR 9 ACTIVATION	1.49E-05	9.81E-05	3659102	32308741	368	2628
KEGG GLYCOSAMINOGLYCAN BIOSYNTHESIS HEPARAN SULFATE	1.58E-05	0.0001032	3659102	32308741	63	325
SIG REGULATION OF THE ACTIN CYTOSKELETON BY RHO GTPASES	1.59E-05	0.00010373	3659102	32308741	102	595
REACTOME INSULIN SYNTHESIS AND PROCESSING	1.66E-05	0.00010757	3659102	32308741	42	190
REACTOME NEUROTRANSMITTER RELEASE CYCLE	1.78E-05	0.00011465	3659102	32308741	97	561
REACTOME SIGNALING BY FGFR	1.94E-05	0.00012449	3659102	32308741	756	5778
ST T CELL SIGNAL TRANSDUCTION	2.55E-05	0.00016311	3659102	32308741	149	946
REACTOME SIGNALING BY NOTCH	2.72E-05	0.00017332	3659102	32308741	653	4950
PID S1P S1P3 PATHWAY	2.82E-05	0.0001788	3659102	32308741	74	406
REACTOME PROTEOLYTIC CLEAVAGE OF SNARE COMPLEX PROTEINS	3.28E-05	0.00020689	3659102	32308741	27	105
KEGG PANCREATIC CANCER	3.30E-05	0.00020732	3659102	32308741	338	2415
KEGG ONE CARBON POOL BY FOLATE	4.45E-05	0.00027511	3659102	32308741	32	136
PID INTEGRIN5 PATHWAY	4.45E-05	0.00027511	3659102	32308741	32	136
REACTOME BRANCHED CHAIN AMINO ACID CATABOLISM	4.45E-05	0.00027511	3659102	32308741	32	136
BIOCARTA AT1R PATHWAY	4.57E-05	0.00028112	3659102	32308741	86	496
BIOCARTA EGF PATHWAY	6.31E-05	0.00038646	3659102	32308741	81	465
REACTOME P75 NTR RECEPTOR MEDIATED SIGNALLING	7.17E-05	0.00043726	3659102	32308741	418	3081
BIOCARTA GABA PATHWAY	7.90E-05	0.00047996	3659102	32308741	15	45
ST DIFFERENTIATION PATHWAY IN PC12 CELLS	8.16E-05	0.00049329	3659102	32308741	146	946
PID CDC42 PATHWAY	8.92E-05	0.00053664	3659102	32308741	334	2415

BIOCARTA CXCR4 PATHWAY	9.03E-05	0.00053842	3659102	32308741	53	276
BIOCARTA EIF4 PATHWAY	9.03E-05	0.00053842	3659102	32308741	53	276
BIOCARTA EDG1 PATHWAY	9.17E-05	0.00054475	3659102	32308741	64	351
REACTOME G2 M DNA DAMAGE CHECKPOINT	9.23E-05	0.00054551	3659102	32308741	13	36
BIOCARTA PAR1 PATHWAY	9.56E-05	0.00056239	3659102	32308741	108	666
PID P75 NTR PATHWAY	9.70E-05	0.00056841	3659102	32308741	325	2346
PID FRA PATHWAY	9.92E-05	0.00057893	3659102	32308741	103	630
PID P38 ALPHA BETA PATHWAY	0.000106	0.00061563	3659102	32308741	80	465
REACTOME NFKB AND MAP KINASES ACTIVATION MEDIATED BY TLR4 SIGNALING REPERTOIRE	0.0001087	0.00062878	3659102	32308741	316	2278
PID ERBB1 INTERNALIZATION PATHWAY	0.0001308	0.00075292	3659102	32308741	128	820
REACTOME INTEGRATION OF ENERGY METABOLISM	0.0001498	0.00085871	3659102	32308741	851	6670
PID TRAIL PATHWAY	0.0001518	0.00086632	3659102	32308741	67	378
BIOCARTA NTHI PATHWAY	0.0001713	0.00097351	3659102	32308741	52	276
REACTOME CHONDROITIN SULFATE BIOSYNTHESIS	0.0001799	0.0010182	3659102	32308741	36	171
PID S1P S1P1 PATHWAY	0.0001839	0.00103612	3659102	32308741	42	210
BIOCARTA PDGF PATHWAY	0.0002045	0.00114264	3659102	32308741	83	496
BIOCARTA TNFR2 PATHWAY	0.0002037	0.00114264	3659102	32308741	33	153
BIOCARTA CHEMICAL PATHWAY	0.000208	0.00115772	3659102	32308741	45	231
KEGG COLORECTAL CANCER	0.0002508	0.00138964	3659102	32308741	264	1891
REACTOME PHOSPHORYLATION OF THE APC C	0.0002564	0.0014148	3659102	32308741	30	136
BIOCARTA SRCRPTP PATHWAY	0.0002804	0.00154118	3659102	32308741	16	55
PID RAC1 PATHWAY	0.0003241	0.00177413	3659102	32308741	198	1378
PID SYNDECAN 4 PATHWAY	0.0003281	0.00178837	3659102	32308741	82	496
BIOCARTA RACCYCD PATHWAY	0.0003342	0.0018143	3659102	32308741	58	325
KEGG SNARE INTERACTIONS IN VESICULAR TRANSPORT	0.0003393	0.00183332	3659102	32308741	105	666
REACTOME METABOLISM OF PORPHYRINS	0.0003405	0.00183332	3659102	32308741	20	78
REACTOME S PHASE	0.0004186	0.00224479	3659102	32308741	711	5565
PID BETA CATENIN NUC PATHWAY	0.0004315	0.00230507	3659102	32308741	419	3160
PID ILK PATHWAY	0.0004437	0.00236064	3659102	32308741	147	990
REACTOME ANTIVIRAL MECHANISM BY IFN STIMULATED GENES	0.0004829	0.00255892	3659102	32308741	285	2080

BIOCARTA TOLL PATHWAY	0.0005069	0.00267509	3659102	32308741	104	666
PID ATF2 PATHWAY	0.0005439	0.00285943	3659102	32308741	231	1653
BIOCARTA IL10 PATHWAY	0.0005801	0.00303769	3659102	32308741	29	136
REACTOME G1 PHASE	0.0006189	0.003228	3659102	32308741	94	595
KEGG BLADDER CANCER	0.0006641	0.00345023	3659102	32308741	129	861
PID CMYB PATHWAY	0.0007294	0.00377494	3659102	32308741	456	3486
REACTOME PRE NOTCH TRANSCRIPTION AND TRANSLATION	0.0008251	0.00425347	3659102	32308741	60	351
BIOCARTA ARENRF2 PATHWAY	0.0009349	0.00476385	3659102	32308741	19	78
SA REG CASCADE OF CYCLIN EXPR	0.0009349	0.00476385	3659102	32308741	19	78
REACTOME EXTRINSIC PATHWAY FOR APOPTOSIS	0.0009349	0.00476385	3659102	32308741	19	78
PID FAS PATHWAY	0.001048	0.0053199	3659102	32308741	107	703
PID MYC ACTIV PATHWAY	0.0012791	0.00644382	3659102	32308741	394	3003
REACTOME NITRIC OXIDE STIMULATES GUANYLATE CYCLASE	0.0012773	0.00644382	3659102	32308741	52	300
REACTOME SIGNALING BY NOTCH1	0.0013136	0.00659279	3659102	32308741	305	2278
PID CXCR4 PATHWAY	0.0013709	0.00685465	3659102	32308741	653	5151
BIOCARTA CD40 PATHWAY	0.0013961	0.00692844	3659102	32308741	23	105
REACTOME GLUTAMATE NEUROTRANSMITTER RELEASE CYCLE	0.0013961	0.00692844	3659102	32308741	23	105
PID ATR PATHWAY	0.001489	0.0073619	3659102	32308741	111	741
PID EPHB FWD PATHWAY	0.0015319	0.00754614	3659102	32308741	116	780
REACTOME ACTIVATED NOTCH1 TRANSMITS SIGNAL TO THE NUCLEUS	0.001656	0.00812722	3659102	32308741	55	325
BIOCARTA NO1 PATHWAY	0.0017306	0.00840021	3659102	32308741	70	435
PID RETINOIC ACID PATHWAY	0.0017306	0.00840021	3659102	32308741	70	435
REACTOME NEGATIVE REGULATORS OF RIG I MDA5 SIGNALING	0.0017306	0.00840021	3659102	32308741	70	435
REACTOME G ALPHA1213 SIGNALLING EVENTS	0.0019272	0.00932062	3659102	32308741	346	2628
PID CXCR3 PATHWAY	0.0020463	0.0098311	3659102	32308741	131	903
REACTOME REGULATION OF MITOTIC CELL CYCLE	0.0020475	0.0098311	3659102	32308741	382	2926
PID TCR JNK PATHWAY	0.0024086	0.01144081	3659102	32308741	18	78
REACTOME PLATELET ADHESION TO EXPOSED COLLAGEN	0.0024084	0.01144081	3659102	32308741	16	66
REACTOME CLASS C 3 METABOTROPIC GLUTAMATE PHEROMONE RECEPTORS	0.0024086	0.01144081	3659102	32308741	18	78

BIOCARTA PGC1A PATHWAY	0.0024937	0.01180283	3659102	32308741	41	231
REACTOME INTERACTIONS OF VPR WITH HOST CELLULAR PROTEINS	0.0028416	0.01340191	3659102	32308741	77	496
PID INSULIN PATHWAY	0.0028563	0.01342371	3659102	32308741	141	990
REACTOME CGMP EFFECTS	0.0030366	0.01422065	3659102	32308741	32	171
PID CIRCADIAN PATHWAY	0.0031485	0.01469304	3659102	32308741	22	105
REACTOME CELL SURFACE INTERACTIONS AT THE VASCULAR WALL	0.0032645	0.01518105	3659102	32308741	447	3486
ST GRANULE CELL SURVIVAL PATHWAY	0.0035296	0.01630011	3659102	32308741	57	351
REACTOME E2F ENABLED INHIBITION OF PRE REPLICATION COMPLEX FORMATION	0.0035205	0.01630011	3659102	32308741	12	45
REACTOME JNK C JUN KINASES PHOSPHORYLATION AND ACTIVATION MEDIATED BY ACTIVATED HUMAN TAK1	0.003975	0.01829338	3659102	32308741	24	120
REACTOME SIGNALING BY TGF BETA RECEPTOR COMPLEX	0.0040611	0.01862491	3659102	32308741	237	1770
REACTOME NOD1 2 SIGNALING PATHWAY	0.0042523	0.01943469	3659102	32308741	64	406
BIOCARTA CERAMIDE PATHWAY	0.0043414	0.01977401	3659102	32308741	40	231
PID S1P META PATHWAY	0.0044174	0.01998326	3659102	32308741	37	210
REACTOME ADP SIGNALLING THROUGH P2RY12	0.0044174	0.01998326	3659102	32308741	37	210
KEGG FC GAMMA R MEDIATED PHAGOCYTOSIS	0.0044866	0.02022766	3659102	32308741	518	4095
ST GA13 PATHWAY	0.0045049	0.02024177	3659102	32308741	98	666
REACTOME GLUCAGON SIGNALING IN METABOLIC REGULATION	0.0045477	0.0203652	3659102	32308741	80	528
BIOCARTA CK1 PATHWAY	0.005261	0.02348047	3659102	32308741	26	136
REACTOME CLASS B 2 SECRETIN FAMILY RECEPTORS	0.0053789	0.02392637	3659102	32308741	464	3655
REACTOME REGULATION OF GLUCOKINASE BY GLUCOKINASE REGULATORY PROTEIN	0.0055178	0.02446211	3659102	32308741	56	351
PID VEGFR1 PATHWAY	0.0057323	0.02532865	3659102	32308741	49	300
REACTOME TGF BETA RECEPTOR SIGNALING IN EMT EPITHELIAL TO MESENCHYMAL TRANSITION	0.0060188	0.02641916	3659102	32308741	19	91
REACTOME SYNTHESIS OF VERY LONG CHAIN FATTY ACYL COAS	0.0060188	0.02641916	3659102	32308741	19	91
BIOCARTA AKAP95 PATHWAY	0.0061868	0.02706709	3659102	32308741	15	66
ST P38 MAPK PATHWAY	0.0062443	0.02722905	3659102	32308741	97	666
KEGG GLYCOSPHINGOLIPID BIOSYNTHESIS GANGLIO SERIES	0.0067421	0.02920824	3659102	32308741	21	105
REACTOME SIGNALLING TO P38 VIA RIT AND RIN	0.0067421	0.02920824	3659102	32308741	21	105

REACTOME TRANS GOLGI NETWORK VESICLE BUDDING	0.0069974	0.03021588	3659102	32308741	220	1653
REACTOME FATTY ACYL COA BIOSYNTHESIS	0.0072148	0.03105398	3659102	32308741	28	153
BIOCARTA CTCF PATHWAY	0.007503	0.03219011	3659102	32308741	42	253
PID S1P S1P2 PATHWAY	0.0080591	0.03413585	3659102	32308741	45	276
REACTOME BASIGIN INTERACTIONS	0.0080591	0.03413585	3659102	32308741	45	276
REACTOME PLATELET CALCIUM HOMEOSTASIS	0.0080443	0.03413585	3659102	32308741	23	120
REACTOME CONVERSION FROM APC C CDC20 TO APC C CDH1 IN LATE ANAPHASE	0.0080443	0.03413585	3659102	32308741	23	120
BIOCARTA STRESS PATHWAY	0.0090529	0.03810227	3659102	32308741	48	300
PID LYMPH ANGIOGENESIS PATHWAY	0.0090529	0.03810227	3659102	32308741	48	300
REACTOME TRANSCRIPTIONAL ACTIVITY OF SMAD2 SMAD3 SMAD4 HETEROTRIMER	0.0096787	0.04060792	3659102	32308741	91	630
REACTOME PKA MEDIATED PHOSPHORYLATION OF CREB	0.0100865	0.04205351	3659102	32308741	25	136
REACTOME GABA SYNTHESIS RELEASE REUPTAKE AND DEGRADATION	0.0100865	0.04205351	3659102	32308741	25	136
REACTOME HYALURONAN UPTAKE AND DEGRADATION	0.0102234	0.04249096	3659102	32308741	11	45
SA G2 AND M PHASES	0.0104156	0.04315508	3659102	32308741	8	28
REACTOME NETRIN1 SIGNALING	0.0106331	0.04391942	3659102	32308741	100	703
REACTOME PHOSPHOLIPASE C MEDIATED CASCADE	0.0110867	0.04565126	3659102	32308741	184	1378
BIOCARTA SPPA PATHWAY	0.0121238	0.04976755	3659102	32308741	38	231

Note:

p value: hypergeometric p value

q value: FDR adjusted p value

n: number of background gene pairs

k: number of interesting gene pairs

m: number of background gene pairs in a pathway

x: number of interesting gene pairs in a pathway

PMID: PubMed ID number.

Pathways are ranked according to q values, with smaller q values listed at the beginning, bigger q values listed at the end.

MSigDB pathways enriched with DR gene pairs identified from $BC_{68\text{-}46}^{Response}$

Pathway Name	p	q	k	n	x	m	PMID
KEGG GLUTATHIONE METABOLISM	0.000	0.000	90561	23286900	32	820	25897982
PID NOTCH PATHWAY	0.000	0.000	90561	23286900	34	1275	26916284
PID TELOMERASE PATHWAY	0.000	0.000	90561	23286900	45	2016	26833480
REACTOME SIGNALING BY NOTCH1	0.000	0.000	90561	23286900	51	1653	26550436
REACTOME RNA POL III TRANSCRIPTION	0.000	0.000	90561	23286900	22	435	19361418
REACTOME SIGNALING BY NOTCH	0.000	0.000	90561	23286900	86	3655	26916284
REACTOME ANTIGEN PROCESSING UBIQUITINATION PROTEASOME DEGRADATION	0.000	0.000	90561	23286900	132	13203	12559039
KEGG PATHWAYS IN CANCER	0.000	0.000	90561	23286900	363	48205	21385051
REACTOME TRANSCRIPTION	5.55E-16	8.20E-14	90561	23286900	133	15576	16557279
REACTOME SIGNALLING BY NGF	2.89E-14	3.84E-12	90561	23286900	148	19110	20569463
REACTOME METABOLISM OF AMINO ACIDS AND DERIVATIVES	5.48E-13	5.61E-11	90561	23286900	122	15225	26838061
REACTOME DNA REPLICATION	2.61E-13	2.89E-11	90561	23286900	123	15225	
REACTOME RNA POL I RNA POL III AND MITOCHONDRIAL TRANSCRIPTION	8.97E-14	1.08E-11	90561	23286900	57	4560	
REACTOME CLASS I MHC MEDIATED ANTIGEN PROCESSING PRESENTATION	7.32E-12	6.08E-10	90561	23286900	143	19701	
PID HDAC CLASSI PATHWAY	1.11E-12	1.05E-10	90561	23286900	32	1711	
REACTOME CELL CYCLE MITOTIC	1.72E-12	1.52E-10	90561	23286900	251	40186	
REACTOME HIV INFECTION	4.75E-10	3.72E-08	90561	23286900	123	17205	
KEGG SMALL CELL LUNG CANCER	1.33E-09	9.34E-08	90561	23286900	41	3486	
REACTOME CELL CYCLE	1.32E-09	9.34E-08	90561	23286900	353	64980	
KEGG CHRONIC MYELOID LEUKEMIA	2.95E-09	1.96E-07	90561	23286900	33	2485	
PID AVB3 INTEGRIN PATHWAY	3.26E-09	2.07E-07	90561	23286900	34	2628	
KEGG NOTCH SIGNALING PATHWAY	3.68E-09	2.22E-07	90561	23286900	18	780	
REACTOME ACTIVATED NOTCH1 TRANSMITS SIGNAL TO THE NUCLEUS	5.56E-09	3.22E-07	90561	23286900	10	190	

REACTOME ADAPTIVE IMMUNE SYSTEM	2.96E-08	1.64E-06	90561	23286900	499	99681
PID HNF3A PATHWAY	6.51E-08	3.47E-06	90561	23286900	16	741
PID MYC REPRESS PATHWAY	7.19E-08	3.68E-06	90561	23286900	25	1770
REACTOME DOWNSTREAM SIGNALING EVENTS OF B CELL RECEPTOR BCR	7.58E-08	3.73E-06	90561	23286900	39	3741
BIOCARTA PTEN PATHWAY	7.91E-08	3.76E-06	90561	23286900	8	136
PID BETA CATENIN NUC PATHWAY	1.14E-07	5.23E-06	90561	23286900	30	2485
PID P53 DOWNSTREAM PATHWAY	2.32E-07	1.03E-05	90561	23286900	62	7750
REACTOME SIGNALING BY PDGF	2.57E-07	1.10E-05	90561	23286900	53	6216
PID RHOA PATHWAY	3.37E-07	1.40E-05	90561	23286900	17	946
PID SMAD2 3NUCLEAR PATHWAY	4.00E-07	1.61E-05	90561	23286900	33	3081
REACTOME SIGNALING BY THE B CELL RECEPTOR BCR	4.36E-07	1.70E-05	90561	23286900	53	6328
KEGG ERBB SIGNALING PATHWAY	7.32E-07	2.78E-05	90561	23286900	34	3321
KEGG MTOR SIGNALING PATHWAY	8.41E-07	3.11E-05	90561	23286900	18	1128
REACTOME RNA POL III TRANSCRIPTION INITIATION FROM TYPE 3 PROMOTER	8.80E-07	3.16E-05	90561	23286900	9	253
REACTOME HOST INTERACTIONS OF HIV FACTORS	9.31E-07	3.26E-05	90561	23286900	54	6670
REACTOME SLC MEDIATED TRANSMEMBRANE TRANSPORT	1.13E-06	3.86E-05	90561	23286900	115	18336
REACTOME DOWNSTREAM SIGNAL TRANSDUCTION	1.33E-06	4.42E-05	90561	23286900	35	3570
KEGG PENTOSE PHOSPHATE PATHWAY	1.80E-06	5.83E-05	90561	23286900	9	276
REACTOME REGULATION OF ORNITHINE DECARBOXYLASE ODC	2.03E-06	6.42E-05	90561	23286900	17	1081
REACTOME NOTCH1 INTRACELLULAR DOMAIN REGULATES TRANSCRIPTION	3.58E-06	0.0001108	90561	23286900	14	780
REACTOME P75 NTR RECEPTOR MEDIATED SIGNALLING	5.46E-06	0.000165	90561	23286900	27	2556
REACTOME NGF SIGNALLING VIA TRKA FROM THE PLASMA MEMBRANE	7.98E-06	0.0002359	90561	23286900	57	7750
PID REG GR PATHWAY	8.20E-06	0.000237	90561	23286900	31	3240
REACTOME PI3K AKT ACTIVATION	9.86E-06	0.0002791	90561	23286900	11	528

REACTOME FACTORS INVOLVED IN MEGAKARYOCYTE DEVELOPMENT AND PLATELET PRODUCTION	1.14E-05	0.0003155	90561	23286900	45	5671
PID HES HEY PATHWAY	1.82E-05	0.0004941	90561	23286900	14	903
REACTOME MITOTIC M M G1 PHASES	2.37E-05	0.0006313	90561	23286900	77	11935
REACTOME S PHASE	2.83E-05	0.0007371	90561	23286900	42	5356
ST INTEGRIN SIGNALING PATHWAY	3.38E-05	0.0008486	90561	23286900	28	3003
REACTOME SYNTHESIS OF DNA	3.33E-05	0.0008486	90561	23286900	33	3828
KEGG MISMATCH REPAIR	4.00E-05	0.0009856	90561	23286900	7	231
REACTOME SIGNALING BY SCF KIT	4.09E-05	0.0009887	90561	23286900	25	2556
REACTOME G1 S TRANSITION	8.75E-05	0.0020782	90561	23286900	41	5460
REACTOME SIGNALING BY ERBB4	0.0001158	0.0026814	90561	23286900	29	3403
REACTOME RNA POL III TRANSCRIPTION TERMINATION	0.0001169	0.0026814	90561	23286900	5	120
KEGG COLORECTAL CANCER	0.0001536	0.0034629	90561	23286900	19	1830
REACTOME GAB1 SIGNALOSOME	0.0001744	0.0038668	90561	23286900	9	496
REACTOME ACTIVATION OF NF KAPPAB IN B CELLS	0.0001928	0.0042027	90561	23286900	18	1711
REACTOME SIGNALING BY EGFR IN CANCER	0.0002666	0.0057186	90561	23286900	35	4656
SIG PIP3 SIGNALING IN CARDIAC MYOCTES	0.0002809	0.0059303	90561	23286900	20	2080
REACTOME CELL CYCLE CHECKPOINTS	0.0003132	0.0065097	90561	23286900	38	5253
REACTOME SIGNALING BY ERBB2	0.0005114	0.010465	90561	23286900	31	4095
REACTOME REGULATION OF APOPTOSIS	0.0006531	0.0131607	90561	23286900	15	1431
PID HEDGEHOG GLI PATHWAY	0.0006947	0.0135881	90561	23286900	11	861
REACTOME METABOLISM OF NON CODING RNA	0.0006947	0.0135881	90561	23286900	11	861
PID INTEGRIN CS PATHWAY	0.0008235	0.0158729	90561	23286900	6	276
KEGG CELL CYCLE	0.0009395	0.017851	90561	23286900	43	6555
REACTOME APOPTOSIS	0.0009825	0.0184051	90561	23286900	56	9180
PID E2F PATHWAY	0.0010305	0.0187757	90561	23286900	21	2485
REACTOME LATE PHASE OF HIV LIFE CYCLE	0.0010287	0.0187757	90561	23286900	33	4656
REACTOME DOWNSTREAM SIGNALING OF ACTIVATED FGFR	0.0010504	0.0188784	90561	23286900	29	3916
KEGG PROSTATE CANCER	0.0010857	0.0192529	90561	23286900	28	3741

REACTOME REGULATION OF MRNA STABILITY BY PROTEINS THAT BIND AU RICH ELEMENTS	0.001119	0.0195829	90561	23286900	23	2850
REACTOME TRANSPORT OF MATURE MRNA DERIVED FROM AN INTRONLESS TRANSCRIPT	0.0012013	0.0207503	90561	23286900	7	406
PID PI3KCI AKT PATHWAY	0.0012551	0.0214011	90561	23286900	8	528
BIOCARTA ETS PATHWAY	0.0013125	0.022097	90561	23286900	4	120
PID CERAMIDE PATHWAY	0.0013485	0.0224183	90561	23286900	12	1081
REACTOME CYCLIN E ASSOCIATED EVENTS DURING G1 S TRANSITION	0.0015645	0.0256882	90561	23286900	17	1891
PID RHOA REG PATHWAY	0.0016747	0.0271621	90561	23286900	10	820
BIOCARTA RACCYCD PATHWAY	0.0018865	0.0302291	90561	23286900	6	325
REACTOME MITOTIC G1 G1 S PHASES	0.0021609	0.0342141	90561	23286900	48	7875

Note:

p value: hypergeometric p value

q value: FDR adjusted p value

n: number of background gene pairs

k: number of interesting gene pairs

m: number of background gene pairs in a pathway

x： number of interesting gene pairs in a pathway

PMID: PubMed ID number.

Pathways are ranked according to the q values, with smaller q values listed at the beginning, bigger q values listed at the end.

MSigDB pathways enriched with DR gene pairs identified from $BC_{61\text{-}19}^{Response}$

Pathway Name	p	q	k	n	x	m	PMID
KEGG CELL ADHESION MOLECULES CAMS	0.000	0.000	9569	23286900	49	7021	21499686
KEGG ANTIGEN PROCESSING AND PRESENTATION	0.000	0.000	9569	23286900	28	3160	25120762
KEGG NATURAL KILLER CELL MEDIATED CYTOTOXICITY	0.000	0.000	9569	23286900	44	7140	19220837
PID CD8 TCR DOWNSTREAM PATHWAY	0.000	0.000	9569	23286900	20	1891	21110320
REACTOME INTERFERON GAMMA SIGNALING	0.000	0.000	9569	23286900	16	1326	16267208
REACTOME INTERFERON SIGNALING	0.000	0.000	9569	23286900	53	10296	19451644
REACTOME IMMUNE SYSTEM	0.000	0.000	9569	23286900	347	296065	26910901
REACTOME ADAPTIVE IMMUNE SYSTEM	0.000	0.000	9569	23286900	153	99681	26884646
REACTOME CYTOKINE SIGNALING IN IMMUNE SYSTEM	0.000	0.000	9569	23286900	95	31125	26975198
NABA ECM AFFILIATED	0.000	0.000	9569	23286900	48	5778	26068592
KEGG INTESTINAL IMMUNE NETWORK FOR IGA PRODUCTION	0.000	0.000	9569	23286900	14	861	
KEGG LEISHMANIA INFECTION	0.000	0.000	9569	23286900	32	2145	
KEGG AUTOIMMUNE THYROID DISEASE	0.000	0.000	9569	23286900	15	1128	
KEGG SYSTEMIC LUPUS ERYTHEMATOSUS	0.000	0.000	9569	23286900	31	5356	
KEGG GRAFT VERSUS HOST DISEASE	0.000	0.000	9569	23286900	20	630	
NABA MATRISOME ASSOCIATED	0.000	0.000	9569	23286900	290	162165	
NABA MATRISOME	0.000	0.000	9569	23286900	401	299151	
REACTOME COSTIMULATION BY THE CD28 FAMILY	2.22E-16	1.64E-14	9569	23286900	15	1485	
REACTOME TCR SIGNALING	8.88E-16	6.22E-14	9569	23286900	13	990	
REACTOME MHC CLASS II ANTIGEN PRESENTATION	4.47E-14	2.98E-12	9569	23286900	17	3081	
REACTOME DOWNSTREAM TCR SIGNALING	4.02E-13	2.55E-11	9569	23286900	9	435	
PID IL12 2PATHWAY	3.29E-11	1.99E-09	9569	23286900	12	1830	
KEGG TYPE I DIABETES MELLITUS	7.04E-11	4.07E-09	9569	23286900	9	780	
REACTOME FATTY ACID TRIACYLGLYCEROL AND KETONE BODY METABOLISM	5.58E-10	3.09E-08	9569	23286900	22	10011	
KEGG VIRAL MYOCARDITIS	3.71E-09	1.97E-07	9569	23286900	11	2211	
KEGG PRION DISEASES	5.41E-09	2.77E-07	9569	23286900	7	561	

KEGG ASTHMA	1.06E-08	5.22E-07	9569	23286900	6	351
REACTOME TRANSCRIPTIONAL REGULATION OF WHITE ADIPOCYTE DIFFERENTIATION	1.94E-08	9.19E-07	9569	23286900	10	2016
REACTOME APOPTOSIS	5.08E-07	2.33E-05	9569	23286900	17	9180
KEGG COMPLEMENT AND COAGULATION CASCADES	4.09E-06	0.0001814	9569	23286900	8	2145
REACTOME GENERATION OF SECOND MESSENGER MOLECULES	4.38E-06	0.0001878	9569	23286900	4	253
REACTOME COMPLEMENT CASCADE	1.17E-05	0.0004864	9569	23286900	4	325
PID CASPASE PATHWAY	1.82E-05	0.0007323	9569	23286900	6	1275
NABA SECRETED FACTORS	2.69E-05	0.0010521	9569	23286900	33	35778
REACTOME PPARA ACTIVATES GENE EXPRESSION	5.57E-05	0.0021158	9569	23286900	9	4005
REACTOME METABOLISM OF LIPIDS AND LIPOPROTEINS	0.0003274	0.0120974	9569	23286900	54	79003
REACTOME PD1 SIGNALING	0.0006748	0.0242547	9569	23286900	2	91
REACTOME INNATE IMMUNE SYSTEM	0.0007145	0.0250068	9569	23286900	21	22791

Note:

p value: hypergeometric p value

q value: FDR adjusted p value

n: number of background gene pairs

k: number of interesting gene pairs

m: number of background gene pairs in a pathway

x： number of interesting gene pairs in a pathway

PMID: PubMed ID number.

Pathways are ranked according to the q values, with smaller q values listed at the beginning, bigger q values listed at the end.
